# Exploring a diverse world of effector domains and amyloid signaling motifs in fungal NLR proteins

**DOI:** 10.1371/journal.pcbi.1010787

**Published:** 2022-12-21

**Authors:** Jakub W. Wojciechowski, Emirhan Tekoglu, Marlena Gąsior-Głogowska, Virginie Coustou, Natalia Szulc, Monika Szefczyk, Marta Kopaczyńska, Sven J. Saupe, Witold Dyrka

**Affiliations:** 1 Katedra Inżynierii Biomedycznej, Wydział Podstawowych Problemów Techniki, Politechnika Wrocławska, Wrocław, Poland; 2 Biyomühendislik Bölümü, Yıldız Teknik Üniversitesi, İstanbul, Turkey; 3 Wydział Chemiczny, Politechnika Wrocławska, Poland; 4 Institut de Biochimie et de Génétique Cellulaire, UMR 5095 CNRS, Université de Bordeaux, Bordeaux, France; 5 Katedra Chemii Bioorganicznej, Wydział Chemiczny, Politechnika Wrocławska, Wrocław, Poland; University of Washington, UNITED STATES

## Abstract

NLR proteins are intracellular receptors constituting a conserved component of the innate immune system of cellular organisms. In fungi, NLRs are characterized by high diversity of architectures and presence of amyloid signaling. Here, we explore the diverse world of effector and signaling domains of fungal NLRs using state-of-the-art bioinformatic methods including MMseqs2 for fast clustering, probabilistic context-free grammars for sequence analysis, and AlphaFold2 deep neural networks for structure prediction. In addition to substantially improving the overall annotation, especially in basidiomycetes, the study identifies novel domains and reveals the structural similarity of MLKL-related HeLo- and Goodbye-like domains forming the most abundant superfamily of fungal NLR effectors. Moreover, compared to previous studies, we found several times more amyloid motif instances, including novel families, and validated aggregating and prion-forming properties of the most abundant of them *in vitro* and *in vivo*. Also, through an extensive *in silico* search, the NLR-associated amyloid signaling was identified in basidiomycetes. The emerging picture highlights similarities and differences in the NLR architectures and amyloid signaling in ascomycetes, basidiomycetes and other branches of life.

## Author summary

All living organisms possess an immune system allowing them to cope with pathogens and, more broadly, to manage interactions with other organisms. One of its conserved components are the so-called NLR proteins, which are found in bacteria, plants, animals and fungi. NLRs are intracellular sensors that trigger a host response upon the detection of non-self markers, which is typically performed by effector domains of NLRs. We investigate the repertoire of these domains in almost 500 fungal strains. We identify several major effector classes, most of which are involved in regulated cell death. Some NLRs do not have built-in effector domains but instead activate separate effector proteins via prion-like signal propagation. This activation is triggered by passing the amyloid fold from a short signaling domain on the NLR to its counterpart on the effector. Using innovative computational approaches, we identify new amyloid signaling motifs and find them overall several times more common in fungal NLRs than previously reported, including the evidence of amyloid signaling in basidiomycetes. Our results describe the global ensemble of NLRs effector domains in fungi and thus enhance our comparative view of this nearly-universally conserved immune receptor family.

## Background

### NLR proteins

All living organisms possess an immune system allowing them to cope with viral or cellular pathogens. Among the central and conserved components of the innate immune system in animals and plants are the NLR proteins. NLRs are intracellular immune receptors that induces various host responses including regulated cell death upon the detection of non-self cues [1–3]. A typical NLR protein functions following a ligand-induced oligomerization and activation process. Its tripartite domain architecture displays 1) a central Nucleotide-binding and Oligomerization Domain (NOD), 2) a C-terminal domain composed of superstructure forming repeats that is typically involved in detection of non-self cues in the form of DAMPs or MAMPs (Damage- or Microbe-Associated Molecular Patterns) and 3) a N-terminal effector domain whose activation induces various downstream host responses including regulation of the infected cell death [4–8]. While historically, NLRs were mostly studied within the animal and plant kingdoms (as Nod-Like Receptors and NBS-LRR Receptors respectively) [9, 10], their homologs were identified in bacteria and fungi [4, 11–13].

In fungi, homologs of NLR proteins were initially identified in the context of the study of a non-self recognition process termed heterokaryon incompatibility [14]. This reaction occurs in filamentous fungi in the event of the fusion (anastasmosis) of the hyphæ of genetically incompatible individuals, resulting in the death of mixed fusion cells [15, 16]. Incompatibility prevents in particular the transmission of mycoviruses between isolates during the anastomosis events. In *Podospora anserina*, HET-E, one of the proteins controlling heterokaryon incompatibility is a homolog of NLR proteins (although its N- and C-terminal domains differ from those known in animals and plants, a situation typical for NLR architecture proteins outside of the plant and animal kingdom [4, 11, 17]). Its central NOD domain is one of the original founding members used to define the NACHT domain (Pfam PF05729) common in animal NLRs (the H in the NACHT acronym stands for HET-E) [10, 18]. The C-terminal domain of HET-E protein, built of hypervariable WD40 repeats recognizes a non-self cue, here polymorphic variants of a host protein termed HET-C, a glycolipid transfer protein universally conserved in eukaryotes that could represent a pathogen effector target [19]. In such event, the N-terminal HET domain of the HET-E protein is activated which ultimately leads to regulated cell death [19]. The HET domain (PF06985) [18] is a cell death inducing domain with a remote homology to TIR domains [20, 21], including conservation of a functionally relevant glutamate [11, 22]. Several other fungal cell death inducing incompatibility pathways in *Podospora* and other species are controlled by NLR proteins [5, 23]. Yet, apparently only a small fraction of the existing fungal NLRs are involved in heterokaryon incompatibility and it is proposed that these proteins have more general functions in immune defense and establishment of symbiotic interactions in fungi [5, 24]. Indeed, NLR proteins are abundant in multicellular filamentous fungi (no NLR protein was found in unicellular yeasts). In a recent study, a total of about 36 000 NLR proteins have been found in around 880 strains of over 560 species of fungi with on average 57 NLRs per genome and numerous species displaying hundreds of NLR genes [5, 11].

In terms of domain annotation fungal NLRs differ from their typical animal and plant counterparts. Unlike more homogenous NLR proteins in animals and plants, the central domain of fungal NLRs can be either of the NACHT [10] or the NB-ARC type (PF00931) [9]. Then fungal NLRs display ankyrin repeats (ANK, Pfam CL0465), tetratricopeptide repeats (TPR, CL0020) and beta-propellers of the WD40 meta-family (CL0186) in place of the LRR repeats found in most animal and plant NLRs. The NBS-TPR architecture was proposed to correspond to the ancestral architecture whilst NLR proteins in multicellular bacteria also typically display TPR, ANK or WD repeats [4, 11, 12, 17]. Consistent with a role in immune defense C-terminal repeated domains of fungal NLRs display marks of positive selection and are highly variable [11, 23, 25]. In addition, the C-terminal domains show original modes of functional diversification. First, about 1/6 of these C-terminal repeat domains consist of highly similar repeats with only a few highly variable positions under positive selection [11, 26]. These repeats arrays with high internal similarity are hypervariable loci in which individual repeats are exchanged and reshuffled resulting in functional diversification [25, 26]. High internal similarity of repeats is both a cause and a result of an unequal crossing over mechanism, a process which is 5–6 orders of magnitude faster than the point mutation [27]. Then, in the truffle *Tuber melanosporum* a superfamily of NACHT-ANK NLR encoding genes displays dozens of 3 bp mini-exons whose alternative splicing can considerably diversify the repertoire of potential C-terminal recognition domain [28]. These striking modes of recognition domain diversification are consistent with the proposed role of NLR proteins in the immune response, as capability of quickly adapting to evolving pathogens is a condition of success in the constant arms race against them [25].

For about 50% of fungal NLR proteins, N-terminal domain annotations could be determined with the Pfam [29] and similar HMM profiles [11], which make up for 12–13 major meta-families [5, 11]. Functionally, the characterized N-terminal domains belong to three basic types: enzymatic, signaling, and regulated cell death induction [30]. The four largest families of fungal NLR effectors are the Alpha/Beta hydrolases [31], the purine and uridine phosphorylases [32, 33], both associated with enzymatic functions, pore-forming domains homologous to HeLo [34–38], and functionally and structurally uncharted Goodbye homologs [11, 37]. The first three families are widespread in various branches of life. For example, the HeLo domain is a fungal homolog of human MLKL, plant RPW8 and bacterial Bell domains [12, 37, 38]. It is understood that upon oligomerization, these domains, whose central part is a four-helix bundle, expel a N-terminal alpha-helix to form a pore targeting the membrane and thus induce cell death [39, 40]. Out of 72 theoritically possible NLR architectures made with the most common domain families (12 types of N-terminal domains, 2 types of central domains and 3 clans of C-terminal domains), as many as 32 were identified in fungal proteomes [11]. Interestingly, in about 20 cases, the closest orthologs of the central domain sequences were bound to different N-terminal domains (including in two different strains of the same species). Moreover, the maximum-likelihood phylogenetic trees generated separately for the N-terminal and central domains were mutually incompatible, and distribution of the N-terminal domains over the branches of central domains trees generated for selected species was scattered. Together with a relatively high number of NLRs without ortholog in other strains of the same species, these findings indicate high plasticity of the architecture of NLR proteins and the occurrence of the *death-and-birth evolution* process [5, 11].

### Amyloid signaling motifs

Another notable feature of fungal NLRs is the occurrence of amyloid-forming motifs at their N-termini [30]. A series of studies derived from the characterization of the *Podospora anserina* [Het-s] prion protein, which controls regulated cell death in the context of heterokaryon incompatibility, has revealed that a fraction of the fungal NLRs employ amyloid signaling to activate downstream cell death effector domains [30, 41]. The paradigmatic example of such amyloid NLR signalosomes is the HET-S/NWD2 two-component system of *P. anserina*. HET-S encodes a cell death execution protein with a globular N-terminal HeLo domain (PF14479) and a C-terminal amyloid forming prion domain composed of two elementary repeats r1 and r2 which are able adopt a specific *β*-solenoid amyloid fold [36, 42–44]. Amyloid transconformation of the C-terminal domain induces activation of the HeLo domain, which turns into a pore-forming toxin. NWD2 is a NLR, encoded by the gene immediately adjacent to *het-S*, and displays at its N-terminus a motif termed r0 which is homologous to the elementary r1 and r2 repeats [37, 41]. When activated by their cognate ligand, engineered variants of NWD2 are capable of triggering transconformation of HET-S and to induce its toxicity. In this system, activation of the NLR leads to amyloid folding of its N-terminus which then serves as template to activate a cognate cell death execution protein [30]. Throughout this paper the term amyloid signaling refers to passing information from one protein to another by transmitting the amyloid fold due to the compatibility of amyloid motifs [30].

The r0, r1, and r2 motifs, collectively referred to as the HET-s *motif*, represent one of the best studied examples of an amyloid signaling motif (ASM). Homologs of the HET-s motif can be grouped in 5 subclasses (collectively denoted as HET-s Related Amyloid Motifs or HRAM) [45], which co-occur in N-termini of fungal NLR proteins and in C-termini of HeLo [34–36] and HeLo-like (PF17111) proteins [11, 37] encoded by genes adjacent to NLR-encoding genes in the genome. In some organisms, two or three subclasses of HRAMs exist simultaneously, which allows for maintaining distinct signaling pathways [45, 46].

There are two other families of fungal ASMs with similar functionality in the NLR protein system, namely *σ* (named after the *σ* prion, which contains this motif [47]) and PP (pseudopalindromic due to the amino acid pattern NxGxQxGxN at its core) [37]. The PP motif bears significant resemblance to the mammalian RHIM motif [38, 48, 49] with remote homologs also in multicellular bacteria [12].

Still, this repertoire of already described fungal ASMs is significantly smaller in comparison to bacterial amyloid signaling motifs. A recent *in silico* analysis of over 100,000 available bacterial genomes in search of sequence motifs repeated in adjacent genes encoding the Bell (bacterial homolog of fungal HeLo) and NLR proteins revealed ten families of Bacterial Amyloid Signal Sequences (BASS) widespread in multicellular Actinomycetes, Cyanobacteria and in Archaea [12]. Despite their sequence-level diversity, at least some if not all known bacterial and fungal ASMs are believed to share the beta-arch fold [50–52].

While it is not fully understood why the NLR/effector pairs involving amyloid signaling are generally encoded by clustered genes, the same situation has been recently reported in regulated cell death pathways involving protease/gasdermine clustered gene pairs [53, 54]. The most likely explanation for this genomic clustering relates to genetic inheritability of such clusters. Genetic association of the genes encoding the receptor and effector moiety of the cell death pathway favors both its vertical (meiotic) and horizontal (transposition driven) inheritance of the pathway as a whole. There is evidence that NLRs in fungi can be preferentially associated with and carried by transposons [55].

When compared to the NLR proteins in plant and animal kingdoms, the fungal NLR proteins display larger diversity of architectures. In addition, NLR-associated amyloid signaling appears specific to fungal and bacterial kingdoms although amyloid motifs also occur in immune pathways in animals [56, 57]. The dominant view, until recently, was that the architecture and immunological function of NLR proteins in plants and animals resulted from the convergent evolution [17]. However, higher diversity of NLRs in fungi than in animals and plants, as well as presence of NLRs in prokaryotes [4, 12, 13] suggest the early evolutionary origins of the architecture and the immune function of NLR proteins [5, 30]. Exploration of the diversity of fungal NLRs is an important asset for deciphering of the potential roles of these immune receptors in fungal biology in addition to their documented role in cell death related to incompatibility. In addition, comparative studies of NLRs in the different kingdoms can provide a more global view of the long term evolution of these central components of immunity in both microbes and macro-organisms. The aim of the current study is to improve the annotation and characterization of the vast ensemble of N-terminal domain of fungal NLRs with particular emphasis on short domains (shorter than 150 amino acids) and amyloid-like motifs.

## Results

### Overview of N-terminal domains of fungal NLRs

**Fig 1:**
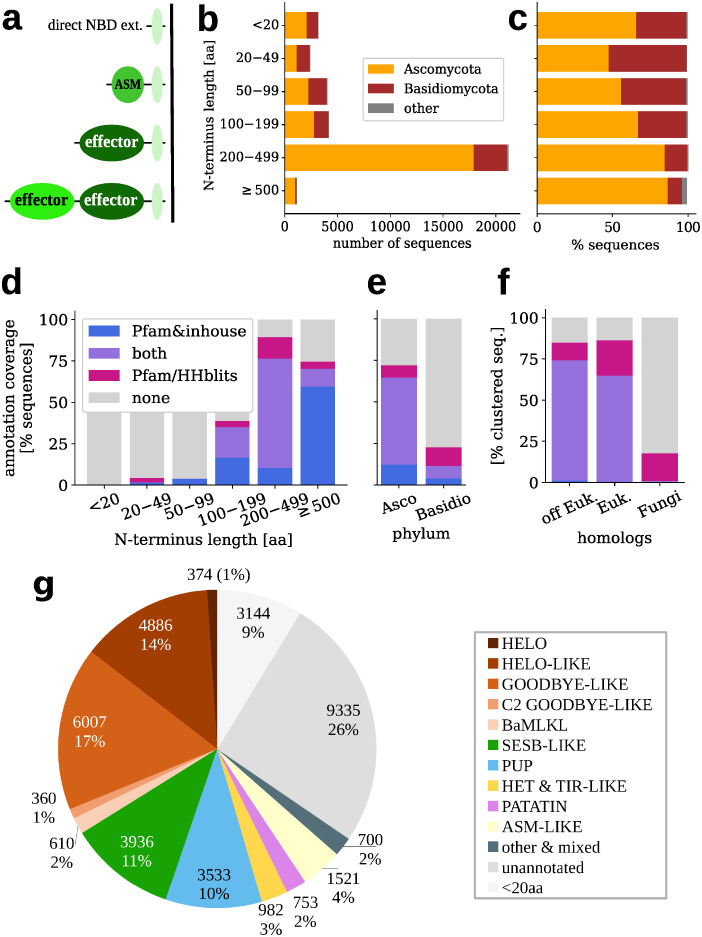
Fungal NLR N-termini. **a**) Major general architectures; **b**) N-termini length distribution with taxonomic division, **c**) Same data scaled to 100% for each length range; Annotation coverage with regard to **d**) N-terminus length, **e**) taxonomic division; **f**) Annotation coverage of the MMSeqs clustered N-termini with regard to presence of taxonomically distant homologs in UniRef top hits (see Results). Euk. denotes *Eukaryota*, off Euk. category includes *Bacteria*, *Archaea* and *Viruses*. Colored bars indicate fraction of Pfam & inhouse annotated sequences (blue: only direct Pfam hits, violet: direct *and* with clustering & HHblits, rose: only with clustering & HHblits). Inhouse profiles were used only for direct Pfam searches. **g**) Distribution of domain families. Additional non-Pfam annotations included, see Results and Methods. N-termini shorter than 20 amino acids are distinguished, as unlikely to contain functional domains.

In roughly 36 000 fungal NLRs identified in a previous study [12], over 90% proteins had N-terminal extension to the NOD domain at least 20 amino-acids long and therefore capable to accommodating a functional domain (Fig 1a). Only 57% of them was previously annotated using the Pfam [29] or inhouse profiles [11]. To improve the Pfam annotation coverage, we clustered the set of N-termini with MMseqs2 [58] and then, for each cluster with at least 20 members, searched for homologs in UniRef30 [59, 60] and subsequently in Pfam using HHblits [61] (see Computational methods for details). The procedure resulted in assigning the Pfam-based annotations to 3003 additional N-termini, thus increasing the annotation coverage to 66%.

#### N-terminal annotations of fungal NLRs are not evenly distributed

The length distribution of N-termini varied significantly with regard to the fungal phylum (Fig 1b and Fig A in S1 Text): while Basidiomycota were over-represented among short N-termini (below 100 amino acids), Ascomycota made up for 85% of termini longer than 200 amino acids. The Pfam annotation coverage was also not evenly distributed. While almost 90% of longer N-terminal domains (200 aa or more) were at least partially annotated, the figure was below 40% for the middle range, and—not surprisingly—a few percent for domains shorter than 100 amino acids, which constituted 1/4 of all NLR N-termini (Fig 1c). The Pfam annotation coverage also strongly depended on taxonomic scope: N-termini from Ascomycota were more completely annotated (72%) than N-termini from Basidiomycota (23%), even though our new clustering-based annotation scheme increased coverage of the latter phylum roughly twice (Fig 1d). This inequality holded as well when N-termini in the same length ranges (above 100 aa) were compared in both branches. In the clustering-based approach, Pfam annotations were found for more than 80% N-termini with the UniRef homologs outside the Fungi kingdom, but only for around 20% sequences with fungal-only homologs (Fig 1e). While better coverage of more universally spread domains is not surprising, taken together, our results highlight the fact that the NLRs of fungi, and especially Basidiomycota, are still not sufficiently represented in Pfam.

#### Novel annotations include the ubiquitin, TIR, and purine nucleoside phosphorylase domains

The updated annotations of fungal NLR N-termini were summarized in Fig 1f and in Fig B in S1 Text. Vast majority of newly added annotations belonged to domain families already described as fungal NLR effectors (Table A in S1 Text). The exceptions were the Crinkler domain of the Ubiquitin clan only recently included in Pfam [62–66], and the Sterile Alpha Motif family SAM_Ste50p [67]. SAMs are involved in homologous and heterologous protein-protein interactions [68], notably they are present in SARM1 protein of the Toll-Interleukin-1 Receptor (TIR) family [69–71]. Moreover, the new scheme increased the number of the Purine and Uridine Phosphorylase (PUP) superfamily annotations, mostly due to the matches to the purine NUcleoside Permease (NUP) profile [72]. In addition, dozens of Pezizomycotina species contained NLR N-termini comprising of C-terminal part of the PNP_UDP_1 fold (cf. pdb:6po4B, residues 176–234). A large number of agaricomycetal N-termini displayed the double domain C2 Goodbye-like architecture [11, 37], the architecture which was specific to Agaricomycetes. The Goodbye-like domain was found also in other double domain architectures of NLR N-termini (Table A in S1 Text). Please refer to S1 Text for additional notes on the updated annotations.

#### Some effector domains are absent in basidiomycetal NLRs

Overall, several most abundant domain classes including the Goodbye-, HeLo-, SesB-like and PUP families, accounted for majority of fungal NLR N-termini (Fig 1f). The two latter superfamilies were common in ascomycetal NLRs (13–14% each) but were almost (SesB-like) or completely (PUP) missing from basidiomycotal NLRs (Fig B in S1 Text). The complete lack of PUP (and HET) domains in basidiomycetal NLRs contrasted with the presence of these domains in other (non-NLR) domain architectures in this division.

### Relation between HeLo-, Goodbye- and basidiomycotal MLKL-likes

#### HeLo- and Goodbye-like annotations overlap in basidiomycetal homologs of human MLKL

Notably, we found clusters with apparently overlapping HeLo/HeLo-like and HeLo-like/Goodbye-like domain annotations. The latter situation was found in Basidiomycota and mostly involved sequences annotated as MLKL_NTD according to Conserved Domain Database (CDD) [73]. Moreover, there were additional basidiomycotal clusters with CDD MLKL_NTD annotation and/or with Pfam HeLo- or Goodbye-like annotations just below the assignment threshold, surmounting to a total of 600 basidiomycotal MLKL-like (BaMLKL) sequences. This made the superfamily of Goodbye/HeLo/MLKL_NTD-like domains the most frequent in Basidiomycota (nearly 2000 sequences, 23% of all), similarly to Ascomycota (10 000 sequences or 38%, Fig B in S1 Text).

**Fig 2:**
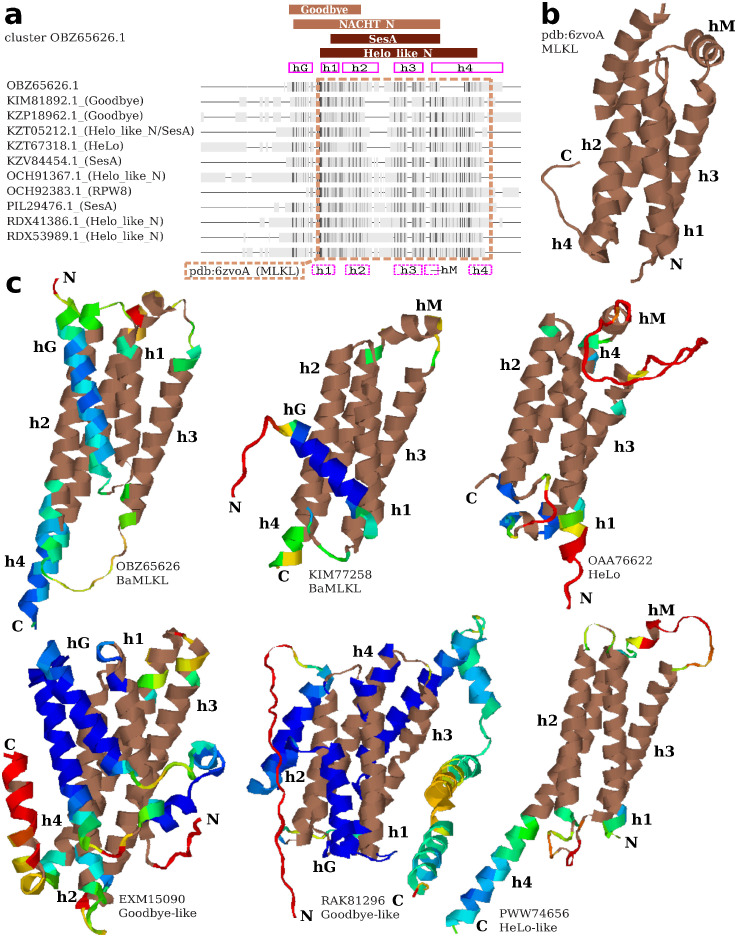
MLKL-like N-termini. **a**) Fingerprint alignment of the doubly (Goodbye-like & Helo-like) annotated OBZ65626 cluster including non-redundant sequences with direct Pfam annotations. The alignment was truncated C-terminally. Darker shade implies higher conservation, while gaps are represented as lines. Columns matched with Pfam profiles of MLKL-like domains are indicated with brown bars. Columns corresponding to helices in a predicted OBZ65626 model are indicated with solid magenta boxes. Columns alignable to the human MLKL structure are framed with a brown dashed line. Columns corresponding to helices in the aligned MLKL structure are indicated with dashed magenta boxes. **b**) The human MLKL structure (pdb:6zvoA). **c**) Structural models of various MLKL-like domains predicted with AlphaFold2 (see Methods). Regions aligned to the human MLKL structure with TM-align are shown in brown. Rainbow colors indicate model quality in terms of pLDDT (below or 50: red, 60: yellow, 70: green, 80: cyan, above 90: blue).

We analyzed the largest cluster with the overlapping Goodbye-like and HeLo-like annotations assigned through the HHblits-based procedure (OBZ65626, 106 sequences). Several sequences in the cluster received also hits from various MLKL-related Pfam profiles when sequences were searched individually (sequence and domain E-values of 1*e* − 3, Fig 2a). Not surprisingly, the multiple sequence alignment of the cluster closely matched (HHpred [74, 75] probability above 98%) the sequence of human MLKL executioner domain with an experimentally solved three-dimensional structure (pdb:6vzo [76], Fig 2b). In fact the MLKL domain was almost perfectly aligned with the Helo_like_N profile match, while the related SesA profile match was slightly shorter. At the same time, the matches to the two Goodbye-like profiles, Goodbye and NACHT_N [11], were both shifted N-terminally with regard to the MLKL-like domain resulting in a partial overlap, significantly longer for NACHT_N. Importantly, the multiple sequence alignment was well conserved for the combined stretch of Goodbye- and HeLo-like matches regardless of Pfam annotations of individual sequences (Fig 2a).

#### HeLo-, Goodbye- and basidiomycotal MLKL-like proteins share a core structural fold

Then, we attempted structure prediction for the largest MLKL-like clusters using AlphaFold2 [77] through the ColabFold advanced notebook [78]. The predictions were carried out solely using multiple sequences alignments of each cluster. Except for the largest HeLo-like cluster, all other predictions resulted in very good quality models (pLDDT around 0.80) sharing a four-helix core (Fig 2c), which is characteristic to the solved MLKL structure. When aligned to the latter using TM-align [79], the predicted models achieved TM-scores between 0.51 to 0.64. The four-helix bundle configuration was supported with alignment conservation scores, calculated with ConSurf [80, 81], which were consistently high for residues facing the interior of the bundle (Fig C in S1 Text). The most notable difference between structural models obtained for various clusters was an additional N-terminal helix in basidomycotal MLKL_NTD homologs and Goodbye-likes (hG in Fig 2), not found in MLKL and HeLo-likes. However, Goodbye-like models presented longer and more complex N-terminal extension than BaMLKLs. Noteworthy was the relatively high conservation of hG residues facing the bundle and h1 residues facing the exterior of the bundle (Fig C in S1 Text). Also, Goodbye-likes lacked a short perpendicular helix (hM) between helices h3 and h4, which seemed to be a common feature of human and basidiomycotal MLKLs and HeLo-likes (Fig 2c).

Taken together, these analyses indicate that although Goodbye-like profiles share a core region with the MLKL bundle and HeLo and Helo-like profiles, they also differ by the presence of an N-terminal extension ahead of the region corresponding to the first helix in MLKL/RPW8/HeLo proteins. Considering the critical role of this region in the oligomerization, membrane targeting and ion specificity of these animal, plant and fungal proteins, further experimental investigation are needed before a potential cell death inducing activity can be firmly attributed to Goodbye-like profiles [39, 40, 44, 82, 83].

### Unannotated longer N-termini

#### A novel helical effector domain is shared between Pezizomycotina and Mortierellomycetes

In addition, largest unannotated clusters were carefully examined and subjected to structural modeling using AlphaFold2 [77, 78] (see Computational methods). The identified domains were listed Table B in S1 Text and briefly characterized in S1 Text. Notably two clusters, mutually homologous, consisted of relatively long domains (N-terminal length above 500 aa) from Pezizomycotina and Mortierellomycetes predicted to be made of multiple alpha-helices forming two stretches of the alpha solenoid-like structure (NLR_Helical in Table B and Fig Dab in S1 Text). Interestingly, homologous domains were also found in bacteria, mainly in *Mycoavidus cysteinexigens*. As this betaproteobacteria is an endosymbiont of *Linnemania (Mortierella) elongata* AG-77 (a fungus with the largest number of these proteins [2]), this may suggest possibility of the horizontal gene transfer.

#### TIR-like effectors are present in Pezizomycotina

Another unannotated cluster consisted of moderately long NLR N-termini (median length of 389 aa), from various Pezizomycotina species, which partially resembled the SEFIR family [84, 85] of TIR clan. A good quality structural model predicted with AlphaFold2 supported homology to TIR and HET domains (Fig Dc in S1 Text). Importantly, the TIR domain was reported in NLRs from plants, bacteria and Chytridiomycota [5, 12, 21, 86]. Interestingly, homologous domains were also present as separate proteins in Mucormycota *Rhizophagus irregularis*, a species related to *Mortierella*, and in *Mycoavidus cysteinexigens*, in accordance with the possibility of horizontal gene transfer [87].

#### Specialized effector domains are abundant in fungal NLRs

Importantly, all other longer domains were represented by less than 100 sequences. With the limitation that in some cases larger families may have been superficially partitioned into small clusters, this indicates that the current Pfam annotations (plus MLKL_NTD and a few inhouse profiles) cover all widely spread abundant domains. At the same time, there seems to exist a substantially large corpus of thousands of specialized N-termini, sometimes confined to narrow taxonomic branches. While some of them may be formed with a tuple of known domains, other could represent novel families (likely being difficult targets for structure prediction due to small alignments). With regard to our previous analyses [5, 11], the current study suggests less diversity in major effector classes (5–7 rather than 12–13), but highlights a likely abundance of specialized domains.

### Amyloid-like motifs in short N-termini

#### A novel *in silico* approach finds amyloid-like motifs in 1/6 of all short NLR N-termini

**Fig 3:**
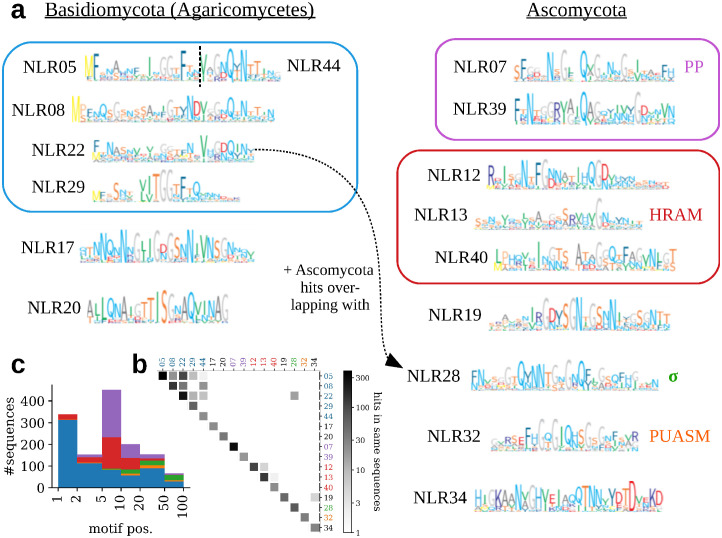
Amyloid-like motifs in short N-termini of NLRs. Clusters of N-termini containing sequences resembling bacterial amyloid signaling motifs were identified using a probabilistic grammatical model [52]. Motifs were extracted with MEME [88] and iteratively refined with profile HMMs. (**a**) Profile HMM-based motif logos—grouped according to overlapping hits in NLR N-termini, as shown in panel (**b**) Overlapping hits in NLR N-termini. See Results and Methods for details. (**c**) Stacked histogram of motif hits positions in NLR N-termini for the five largest motif families, color-coded as in panel (a)).

The largest deficiency in the annotation coverage concerned short N-terminal sequences (length below 150 amino acids). Only less than six percent of them (645 out of 11 634) received any Pfam-based annotation, while less than two percent (214) was annotated as so called prion-forming domains (PFD) [11, 37], consisting of the three known families of fungal ASMs. As more than 3 000 short N-terminal domains were assigned to clusters made with at least 20 sequences, this suggested presence of conserved sequential features. Therefore, we searched for potential additional fungal amyloid signaling motifs using an approach that combined filtering with a probabilistic grammatical model inferred from ten families of bacterial ASMs (BASS1–10 [12]), shown to be sensitive to fungal amyloid signaling sequences [52], with the MEME motif extraction [88] (see Computational methods for details). The procedure resulted in identifying 16 grammar-compatible motifs (Fig 3a. Then, we used profile HMMs of these motifs to scan all NLR N-termini at least 10 amino-acids long, and found hits in 1537 sequences (Table 1), which represented 17% (36%) of all (clustered) short N-termini. The number included 204 out of all 242 sequences already annotated as PFD-LIKEs (84% sensitivity).

**Table 1:** Amyloid-like motifs in short N-termini of NLRs. Motif id indicates ranks in the MEME output. Motifs are grouped based on overlapping hits in NLRs and similar sequence patterns. Established and proposed motif annotation labels are given where applicable. L is the motif length. #NLR and #nei. indicate number of sequences with a given motif in short N-termini of NLRs and C-termini of their genomic neighbors, respectively. AC+ indicates a proportion of motif instances for which ArchCandy score is 0.56 or above. Major taxonomic branch including vast majority of NLRs with the motif is given. #eff. indicates total number of effector proteins (with established association to NLRs) [5] with a given motif in C-termini. #cooc. indicates number of sequences with a given motif in short N-termini of NLRs / C-termini of effector proteins cooccurring in the same strains (genome assemblies). #str. is a number of such strains with cooccurrence. Exp. indicates selected studies reporting experimental validation of some properties typical to ASM for a motif instance in a given family.

Id	Annot.	L	#NLR	AC+	Major tax.	#nei.	#eff.	#cooc.	#str.	Exp.
05	—	17	387	0.28	Agaricomycetes	0	0	—	—	—
08	—	30	138	0.55	Agaricomycetes	0	0	—	—	—
22	—	24	263	0.53	Agaricomycetes	0	0	—	—	—
29	—	20	60	0.53	Agaricales	0	0	—	—	—
44	—	12	24	0.00	Agaricomycetes	0	0	—	—	—
07	PP	23	296	0.64	Ascomycota	37	106	157/76	56	[106]
39	PP	24	20	0.80	Ascomycota	0	2	1/1	1	—
12	HRAM3	26	110	0.48	Sordariomycetes	4	17	80/14	13	—
13	HRAM	24	131	0.57	Ascomycota	16	43	41/24	20	[43, 46]
40	HRAM	26	24	0.92	Ascomycota	0	0	—	—	—
17	—	26	24	0.88	*A. muscaria*	0	0	—	—	—
19	—	27	53	0.60	*Tuber*	0	0	—	—	—
20	—	21	37	0.68	Agaricales	0	0	—	—	—
28	*σ*	28	71	0.80	Ascomycota	42	62	45/43	40	[52]
32	*PUASM*	22	33	0.45	Sordariomycetes	11	29	25/21	18	this study
34	—	28	29	0.52	Ascomycota	0	0	—	—	—

#### Amyloid-like motifs in fungal NLRs cluster to nine classes likely assuming the beta-arch fold

Not surprisingly, some of the 16 motifs clearly corresponded to the three fungal ASM families: HRAM (NLR13, found in 131 sequences), PP (NLR07, 296), and *σ* (NLR28, 71). The overall recall of 498 hits was twice higher in comparison to the combined Pfam-based approaches (242). Several hits of another two motifs, NLR12 and NLR40, overlapped with the NLR13 (HRAM) matches (Fig 3b). Moreover, the HMM scan with a generalized HRAM profile based on HRAM dataset from [45] recognized 27/51 NLR12 and 14/22 NLR40 motifs, thus indicating that these two classes were related to HRAM. Indeed, the NLR12 motif (Fig 3a) is apparently similar to HRAM3 [45]. In addition, the G-hydrophobic-Q-hydrophobic-G pattern of NLR39 motif resembled NLR07 (PP). Five other motifs (NLR17, NLR19, NLR20, NLR32 and NLR34, in 138 sequences altogether) were difficult to assign to the known families. The final and the largest subgroup (689 sequences) consisted of five motifs (NLR05/08/22/29/44) with hits substantially overlapping NLR22 hits. This large group was specific to basidiomycetes except of a dozen of NLR22 hits overlapping ascomycotal NLR28 (*σ*) (Fig 3b). While most motifs were distributed in larger taxonomic branches, two motifs were more restricted: NLR17 was specific to *Amanita muscaria* (strain Koide) and NLR19 to genus *Tuber*. A combined NLR19 + NLR34 configuration was found in five highly homologous sequences from *Tuber melanosporum* (Fig 3b). All 16 motifs are likely to assume the beta-arch fold typical to known fungal and bacterial ASMs as from 45 to 95% motif instances passed the fold prediction threshold of ArchCandy (column AC+ in Table 1). The only exceptions were two shortest motifs, NLR05 (28%) and NLR44 (none), probably because they comprise only parts of the actual amyloid-like motif (Fig 3a).

#### For four motifs, the amyloid signaling is supported by genomic co-localization of effectors

Significant numbers of similar sequence stretches in C-termini (100aa) of genomically neighboring (20kbp) proteins were found only for motifs representing the three fungal ASM families (NLR07 in 37 sequences, NLR12 in 4, NLR13 in 16, and NLR28 in 42) and for NLR32 (in 11 sequences). This suggests that NLR32 defines a new family of amyloid signaling motifs. (For further computational and experimental verification, see below).

#### Amyloid-like motifs differ in their position in NLR N-termini

While instances of the NLR05/22 group were usually situated in the very terminus, most HRAMs (NLR12/13/40) and PPs (NLR07/39) were located at positions 5–9. Moreover, NLR32 and *σ* motifs (NLR28) were shifted further C-terminally with relative majority at positions 20–49 and 50–99, respectively (Fig 3c). In addition, a couple of dozens of amyloid-like sequences of various families (including 17 NLR05 and 7 NLR07) were found located centrally or C-terminally in longer N-termini. Some of them formed combined architectures with annotated domains, most notably with NLR_PRDR (NLR05 in 10 sequences from *A.bisporus*) and MLKL-likes (5 BaMLKL + NLR05 in *Laccaria bicolor*, 4 HeLo-like + NLR28 and 1 HeLo-like + NLR07 in various Ascomycota).

### A reverse approach: Amyloid-like motifs in C-termini of effector proteins

#### Two novel amyloid-like motifs are uniquely associated with the PNP_UDP effector domain

In order to complement the search for amyloid signaling motifs in NLRs and verify discovery of the fourth NLR-related fungal ASM family, we adapted the approach recently used for identification of 10 families of bacterial ASMs in NLR-related proteins in bacteria [12]. The procedure, which also used MEME for motif extraction, started from known effector proteins [5], and relied on genomic proximity of their genes and genes encoding NLRs (see Computational methods for details). Consequently, we identified 22 motifs, and clustered them on the basis of their co-occurrence in 190 pairs of genomically neighboring proteins (Fig E in S1 Text). Three clusters clearly corresponded to the already known families PP, *σ*, and HRAM (Fig E in S1 Text). Two additional motifs with few pairs apparently resembled HRAM2 and HRAM4 [45], respectively. The fourth largest family of motifs exhibited a distinctive conserved pattern FxGxGxQxxGxGxF, which clearly corresponded to the NLR32 motif in Fig 3. Since in both searches the motif was found associated uniquely with the PNP_UDP domain, we termed it PUASM, or the Pnp_Udp-associated Amyloid Signaling Motif. The NLRs with the PUASM motif proteins were annotated either as NACHT or NACHT WD40. All matched instances of the PUASM motif came from various Pezizomycotina species. Finally, we found one more distinct motif related to PNP_UDP, however only present in four pairs (PF01048_015 in Fig E in S1 Text).

#### Amyloid-like motifs differ in the effector domain association

Overall, the ASM differed in type of associated effector domain, either pore-forming (HeLo and HeLo-like for HRAM/NLR13), enzymatic (PNP_UDP for HRAM/NLR12, NLR32 and PF01048_015), or both (PP/NLR07 and *σ*/NLR28). Interestingly, while the NLR13 motif was typically found as a double in C-termini of HeLo and HeLo-like domains, for the second HRAM-like, NLR12, only single instances were found in C-termini of PNP_UDP_1 effector proteins. This may suggest a different mode of operation despite their similar sequence profiles. Notably, the occurrence of ASMs as single instances or two (or three) fold repeats was also reported for bacterial ASMs [12].

#### Amyloid signaling suspected between NLRs and effectors encoded by non-adjacent genes

To check the possibility that proteins cooperating through the amyloid signaling are encoded by non-adjacent genes, we analyzed co-occurrence of particular amyloid-like motifs in N-termini of NLRs and C-termini of established effector domains [5] in entire genomes. Non-singular C-terminal hits and genomic co-occurrences were found only for the three established fungal ASM families and PUASM (Table 1). Such cases were relatively most frequent for HRAM/NLR12, in parallel with the high ratio between the NLR-side and the effector-side motifs in some genomes (mean ratio 5.7:1, Table 1).

### Amyloid-like motifs in Basidiomycota

#### Genome-wide motif searches suggest the NLR-related amyloid signaling in Agaricomycetes

**Fig 4:**
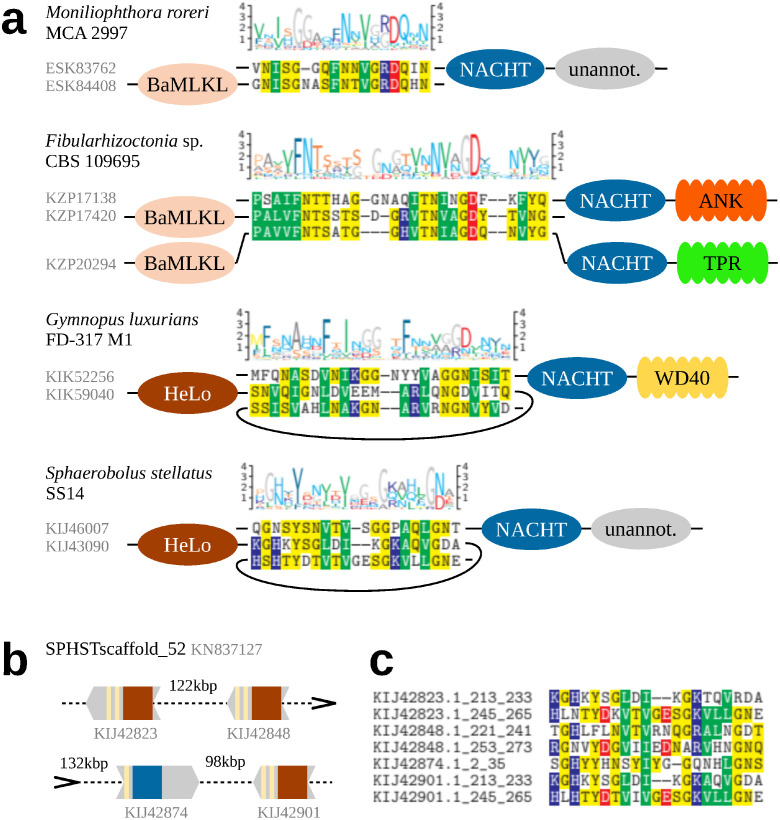
Potentially interacting amyloid-like motifs in Agaricomycetes. (**a**) Motif logos, sequence alignments and domain architectures of selected motif instances. (**b**) Schematic representation of a cluster of amyloid-like motifs in contig SPHSTscaffold_52 from genome assembly GCA_000827215.1 of *Sphaerobolus stellatus* SS14. (**c**) Multiple sequence alignment of motif instances in (b).

With the NLR-related amyloid signaling previously described in multicellular bacteria and Ascomycota, apparent is the lack of evidence of this mechanism in Basidiomycota. On the other hand, we found numerous homologs of the pore-forming HeLo and HeLo-like domains in Basidiomycotal NLRs. Thus, we used them for searching the entire Basidiomycota genomes for homologs separate from NLR domains. We identified hundreds of such putative singular pore-forming domains, which—because of their potential to cause the cell death—can be expected to be under control of other proteins. As in Ascomycota such control is exerted by NLRs through the amyloid signaling sequences, we scanned the identified BaMLKL homologs against ASM profiles and grammars. However, fragments resembling ASMs were identified only in a few out of 500 sequences and in no case similar fragments were found in the neighboring NLRs. Yet in two cases pairs of amyloid-like motif instances occurred when entire genomes were considered (Fig 4a). In *Moniliophthora roreri* (strain MCA 2997) there was a 18 amino-acid long motif apparently shared between two BaMLKL C-termini and 26 short NACHT N-termini (Fig F in S1 Text). In addition, in *Fibularhizoctonia* sp. CBS 109695 there was a conserved pattern shared between two BaMLKL C-termini, eight short NLR N-termini (including KZP25847 with NLR20 instance), and additional five NLR proteins with the pattern situated between BaMLKL and NACHT domains (including KZP30127 and KZP3012 with NLR22 instances)—see alignment in Fig G in S1 Text. It would suggest a possibility that in *Fibularhizoctonia* proteins with the N-terminal and C-terminal amyloid-like sequences were pseudogenes, especially that three NLRs in this group were atypically short (less than 200 amino acids). However, NLRs with N-terminal and mid-sequence ASMs differed in domain configuration with the former belonging to NACHT, NACHT ANK and NACHT VHS architectures, while the latter were all of the NACHT TPR type (Fig 4a). (In *M. rorei*, we found only one protein with the BaMLKL + NOD architecture (ESK90106.1) and the linker sequence between the domains did not resemble an amyloid-like motif).

#### Amyloid-like motifs in agaricomycetal NLRs share features with the HET-s motif homologs

In addition, we investigated two Agaricomycetes species with proteins comprising of a singular HeLo domain and a C-terminal double HET-s motif. In the genome of *Sphaerobolus stellatus* (strain SS14), which included four such C-termini, we found at least eight NACHT NLRs with N-termini comprising of single HRAM-like sequences (Fig H in S1 Text). Two instances (in KIJ28522 and KIJ30800) resembled the NLR13 HRAM motif. This strain was the only case where an NLR and three HeLo proteins were situated on a single contig in genome assembly (Fig 4b and 4c). The shortest distance between genes encoding NLR and HeLo was relatively large 95 kbp. The second species, *Gymnopus luxurians* (strain FD-317 M1), included one protein with HeLo + double HET-s motif architecture. While we did not find any typical HRAMs in N-termini of 200 NLRs, several dozens included an instance of the NLR05/08/22/44 motif meta-family. When fragments of N-terminal sequences best-fitting the PCFG model were aligned, it revealed a 25-residue long core pattern. Interestingly, the alignment exhibited features characteristic to HRAMs: the N-terminal pattern of three hydrophobic residues and the C-terminal G[DN] bigram (Fig I in S1 Text). In total 32 amyloid-like motif instances were associated with NB-ARC, NACHT, NACHT WD and NACHT TPR domain architectures. Taken together these analyses strongly suggest that the NLR-associated amyloid signaling process also occurs in Basidiomycota.

#### N-terminal amyloid motifs often found in dozens of NLRs per basidiomycetal strain

Taken together, presented results support the presence of amyloid signaling in Basidiomycota, or more specifically in Agaricomycetes, in the context of NLR-based regulation of HeLo-/MLKL_NTD-likes. Moreover, they suggest that NLR05/08/22/29/44 meta-family of motifs is a basidiomycotal variety of the HRAM motif or its homolog. However, there were significant differences with regard to Ascomycota. First, while NLR-side amyloid signaling motifs were present in roughly half of Ascomycota strains, they were only found in 1/4 (30%) of Basidiomycota (Agaricomycetes) strains. Second, while there were typically only few amyloid signaling sequences per ascomycotal strain, there were usually dozens per basidiomycotal strain. At the same time, basidiomycotal effector-side C-terminal ASM sequences were seemingly less frequent than NLR-side N-terminal ASM sequences (Fig F–I in S1 Text). Indeed, the high number of NLR-side ASM sequences corresponded to enrichment of basidiomycotal sequences among shorter N-terminal domains (Fig 1b and Fig A in S1 Text).

### Experimental validation of a novel amyloid signaling motif

#### PUASM displays sequence patterns typical to amyloid-like motifs

**Fig 5:**
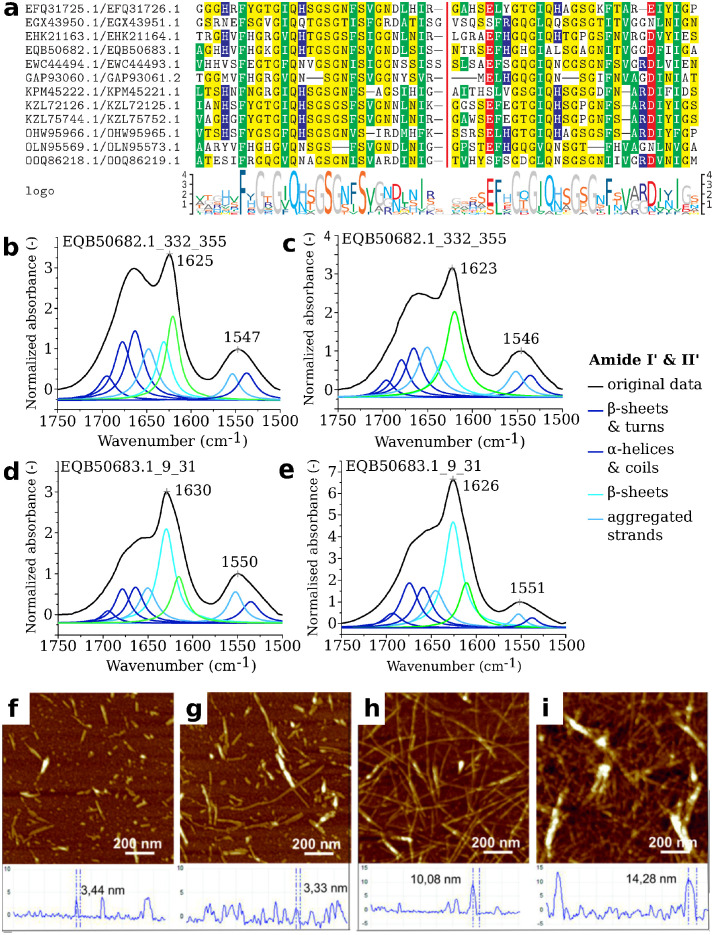
The PUASM motif. Alignment of the PUASM sequence pairs (**a**) from effector C-terminus (left) and NLR N-terminus (right). Colors indicate residue hydrophobicity, curly brackets—the motif ranges. Deconvolution of ATR–FTIR spectra of air-dried peptide films of EQB50682.1_332_355 (**bc**) and EQB50683.1_9_31 (**de**) in the amide bands region (1750–1500 cm^−1^). Spectra registered at 20°C (68°F) after dissolving (**bd**) or after 40 days of incubation at 37°C (98.6°F) (**ce**). AFM images with cross-section profiles of peptides EQB50682.1_332_355 (**fh**) and EQB50683.1_9_31 (**gi**). Samples imaged after dissolving (**fg**) or after 40 days of incubation at 37°C (98.6°F) (**hi**).

The alignment of PUASM instances (Fig 5a) revealed high similarity of PNP_UDP- and NLR-side sequences in the core region covered with the NANBNtm_035 pattern. Some divergence was present C-terminally, with pattern GND prevailing in PNP_UDP-side motifs and pattern ARD in NLR-side motifs. Interestingly, these 3-mers can be found in C-termini of already known amyloid signaling motifs HRAM1 [45] and BASS2 [12], respectively. Further four residues of the C-terminal extension of the motif exhibited a hydrophobic pattern well-conserved in pairwise alignments (Fig 5a). On the other side, N-terminal extensions of the PUASM profile matches often included histidine on the PNP_UDP side and glutamic acid on the NLR side. This, together with the overall composition of the N-terminal extensions, suggests some role of the charge complementarity.

#### Aggregation of synthetic PUASM peptides examined with ATR-FTIR, AFM and ThT assay

To check if biochemical properties of PUASM are consistent with its presumed role as the amyloid signaling motif, we experimentally analyzed a representative pair of motifs of this family, namely, PNP_UDP-side C-terminal EQB50682.1_332_355 and NLR-side N-terminal EQB50683.1_9_31 from a plant pathogenic fungus *Colletotrichum gloeosporioides* Cg-14 [89] (Table C and Fig J in S1 Text). The selected fragments entirely covered the matches of PUASM profiles and the pairwisely conserved C-terminal extensions. The aggregation propensities of the PUASM peptides were determined experimentally using the Attenuated Total Reflectance—Fourier Transform Infrared spectroscopy (ATR-FTIR), Atomic Force Microscopy (AFM), and the Thioflavin T fluorescence assay (ThT). The ATR-FTIR spectroscopy allows determination of secondary structure and monitoring structural changes of peptides upon aggregation processes [90–92], while AFM is useful for detection and visualization of aggregates [93]. In turn, ThT is considered to be the “gold standard” for identifying amyloid fibrils [94, 95]. It is widely accepted that such a combination of experimental techniques is necessary to ascertain whether a particular peptide or protein is able to form the amyloid assemblies [96–98].

#### PUASM peptides display intramolecular *β*-structures and intermolecular *β*-sheets

Analysis of the ATR-FTIR spectra in the range of 1750–1500 cm^−1^ (Fig 5b, 5c, 5d and 5e, Fig K, Table D and Table E in S1 Text) confirmed aggregation properties of studied peptides. The position of the Amide I’ band maximum was observed at 1625 cm^−1^ and 1630 cm^−1^ for EQB50682.1_332_355 and EQB50683.1_9_31, respectively. This signature is considered to be a spectroscopic marker of the cross-*β* amyloid architecture [91, 99]. High absorbances in the region of 1670–1660 cm^−1^ were observed in both spectra. The assignment of this band is still discussed in the literature [100–102]. The overall spectral line in Amide I’ was similar to the spectra observed for *β*-solenoidal proteins, including HET-s [103] and PrP^Sc^ [104]. While for both studied peptides the aggregation process was observed immediately after dissolving, N-terminal EQB50683.1_9_31 aggregated quicker and formed more well-ordered structures [105] (Fig 5b and 5d). A band curve-fitting method allowed to resolve individual Amide I’ band components and obtain a more detailed information about secondary structure of studied peptides. In the wavenumber range of 1640–1610 cm^−1^ two components were visible. The subband at about 1635 cm^−1^ corresponds to intramolecular *β*-structures. The percentage area of this component was 31% and 14% for peptide EQB50683.1_9_31 and EQB50682.1_332_355, respectively. In turn, the second component at about 1620 cm^−1^ corresponds to intermolecular *β*-sheets. Peptide EQB50683.1_9_31 displayed this subband at 1616 cm^−1^, while EQB50682.1_332_355 at 1620 cm^−1^, indicating a looser fibrillar structure of the latter.

#### PUASM peptides form amyloid-like aggregates that elongate during incubation

Atomic Force Microscopy images of both PUASM peptides were acquired for two conditions related to the spectroscopy studies: after dissolving, and after 40 days of incubation at 37°C. The aggregation process of the peptides was present already in the sample after dissolving as the fibers with height of 3.44 ± 0.3 nm and 3.33 ± 0.3 nm, respectively for EQB50682.1_332_355 and EQB50683.1_9_31, were observed (Fig 5f and 5g). The height of the object observable in AFM is comparable with the size of the HET-s peptides obtained by the solid-state NMR technique (pdb:2kj3) [43]. Peptide aggregation was further enhanced in the samples imaged after 40 days of incubation at 37°C (Fig 5h and 5i), when the height of the aggregates reached 10.08 ± 0.9 nm and 14.28 ± 1.3 nm, respectively for EQB50682.1_332_355 and EQB50683.1_9_31. This clearly visible increasing aggregation process was in line with the ATR-FTIR measurements (Fig 5c and 5e).

#### PUASM peptides show an increase in ThT fluorescence in the assembly process

Thioflavin T (ThT) fluorescence assay is the most common assay to follow amyloid formation. We thus determined whether the PUASM peptides bind ThT. We observed an increase in ThT fluorescence over time with a sigmoidal curve for PNP_UDP-side C-terminal peptide EQB50682.1_332_355, starting with a lag phase of 2 hours (Fig L in S1 Text), followed by a rapid growth phase from 2–2.20 h, and ending at a stable plateau with the maximum ThT intensity. A significant increase in the fluorescence emission was observed for NLR-side N-terminal peptide EQB50683.1_9_31 (about 5 times higher than for EQB50682.1_332_355). The lag phase was not observed (Fig L in S1 Text). The steeper ThT curve with quicker attainment of plateau might indicate faster aggregation process of peptide EQB50683.1_9_31 in comparison to peptide EQB50682.1_332_355. While it is clear that both peptides showed an increase in ThT fluorescence during the assembly process, the presence of short fibrils in the AFM study complicate the comparative study of the aggregation kinetics of the two peptides.

#### GFP-PUASM spontaneously forms cytoplasmic foci *in vivo* alike other amyloid-like motifs

**Fig 6:**
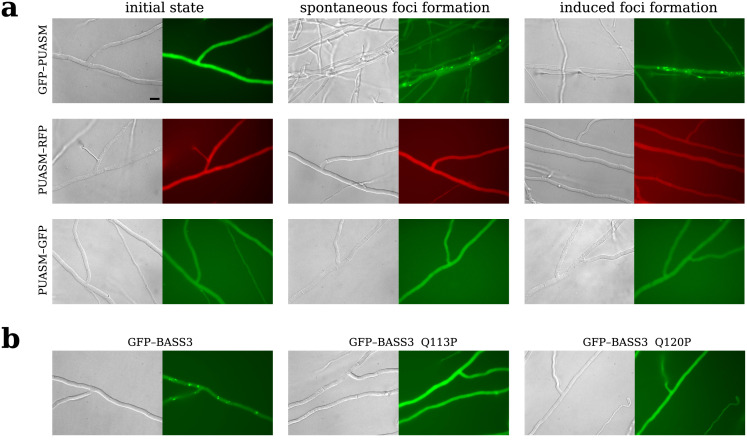
Expression of GFP/RFP-fused PUASM motifs in *Podospora anserina*. **a**) Micrographs of *P. anserina* strains expressing molecular fusions of PUASM with GFP or RFP, as indicated on the left; scale bar: 5 *μ*m. Strains were analyzed in their initial state after transfection (left panels marked) and either after several days of subculturing (middle panels, spontanous foci formation) or after cytoplasmic contact with a strain expressing GFP–PUASM in the foci state (right panels, induced foci formation). Note that the GFP–PUASM construct (but not PUASM–RFP and PUASM–GFP) leads to spontaneous and induced foci-formation. Quantification of the rate of foci formation is given in Table F in S1 Text. **b**) Micrographs of strains expressing GFP–BASS3—positive control for the spontanous foci formation (BASS3 motif of WP_037701008.1 of *Streptomyces atratus*, positions 70 to 124, left panel), and two GFP–BASS3 mutants—negative controls (Q113P, middle, and Q120P, right panel).

It was previously reported that fungal, bacterial and mammalian amyloid motifs could form prions *in vivo* in the *Podospora anserina* model [12, 41, 46, 106]. To determine if PUASMs could also form prions in vivo, we expressed the PNP_UDP-side C-terminal EQB50682.1_332_355 from a plant pathogenic fungus *Colletotrichum gloeosporioides* Cg-14 [89] in *P. anserina* as GFP or RFP fusions. Three different constructs were generated: a N-terminal GFP fusion (GFP-PUASM) and C-terminal RFP and GFP fusions (respectively PUASM–RFP and PUASM–GFP). The three constructs were expressed from a strong constitutive promotor. In GFP–PUASM, the motif thus occurs C-terminally to the GFP domain, an organization that is analogous to that of the native full length EQB50682.1, in which the motif occurs C-terminally to the phosphorylase domain (PF01048). Prion formation was monitored using fluorescence microscopy by following the formation of cytoplasmic fluorescent foci as previously described for other amyloid signaling motifs expressed in *P. anserina* [12, 41, 46, 106]. A GFP fusion with an instance of previously characterized ASM (BASS3 of Streptomyces atratus) was used as positive control and its two proline mutants (BASS3 Q113P and Q120P) were used as negative controls [12]. GFP–BASS3 led to foci formation while the proline mutants did not. The GFP-PUASM fusion led initially to a diffused fluorescence signal (Fig 6, Table F in S1 Text). Upon subculturing, the number of transformants showing cytoplasmic foci gradually increased over time as typically observed for other prion amyloid motifs [106] (Table F in S1 Text). In contrast to the GFP–PUASM construct, fusion constructs displaying the motif N-terminally (PUASM–GFP and PUASM–RFP) remained diffused and did not form foci even upon prolonged subsculturing. A similar situation was observed previously for the HELLF and RHIM motifs for which N-terminal position of the GFP/RFP inhibited foci formation [106]. We conclude from these experiments, that GFP–PUASM (but not RFP–PUASM and GFP–PUASM) spontaneously forms cytoplasmic foci as previously reported for other amyloid signaling motifs.

#### GFP-PUASM behaves as a prion *in vivo* in the *Podospora* model

To determine whether the foci state is infectious, strains expressing GFP–PUASM, PUASM–GFP or PUASM–RFP in the diffuse state were confronted to strains expressing GFP-PUASM in the foci state to induce cytoplasmic contact. At several time points after cytoplasmic contact, the recipient strains were subcultured and monitored to presence of foci (Table F in S1 Text). In this induced prion formation assay, GFP–PUASM strains were efficiently converted to the foci state after cytoplasmic contact with a GFP-PUASM strain in the foci state (Fig 6, Table F in S1 Text). In 96 hours after contact with the inducing strain, all tested strains displayed dots. In comparison, spontaneous dot formation was only detected in about 3% of the strains after 5 days of subculturing (Table F in S1 Text). Thus contact with a strain expressing GFP–PUASM dots induced dot formation in the recipient strain. Again, for the PUASM-GFP and PUASM-RFP proteins prion conversion was not observed. After confrontation with a strain expressing GFP–PUASM foci, when strains were subcultured no foci formation was detected. We conclude from these experiments that the GFP–PUASM fusion protein behaved as a prion *in vivo* in the *Podospora* model. Apparently, as in the case of other amyloid signaling motifs, the C-terminal position of the GFP/RFP inhibited foci formation [106]. In addition, the spontaneous and induced prion conversion of GFP–PUASM was somewhat less efficient than for other amyloid motifs that have been previously tested in the same way [12, 46, 106].

## Discussion

In previous studies we computationally screened N-terminal domains of fungal NLRs using profile Hidden Markov Models (HMM) from the Pfam database directly and complemented the search with several Pfam-like inhouse models [5, 11]. Here we expanded the most recent analysis with a more sensitive search using the state-of-the-art clustering offered by MMseqs2 and HMM–HMM searches with HHblits. The study increased the overall Pfam annotation coverage of N-terminal domains by about 16% (or 19% when MLKL_NTD from CDD is counted), but also highlighted remarkable deficiencies in availability of annotations. Our results highlight the fact that the NLRs of fungi, and especially Basidiomycota, are still not sufficiently described.

### Goodbye resembles Helo but with an additional N-terminal extension

The identification of a common structural core of Helo-like and Goodbye-like domains, the four-helix bundle, raises the question of their functional similarity. Both the distribution of associated nucleotide-binding domain and C-terminal domains, and the paralog-to-ortholog ratio for Goodbye-like and HeLo-like domains are similar [11], which may suggest similarities in their mode of operation. However, Goodbye-likes in NLR N-termini are often associated with another annotated effector domains, which is untypical for HeLo-likes. Moreover, the opposite is true for association with the amyloid signaling motifs, which is common to HeLos, HeLo-likes and basidiomycotal MLKL_NTDs but not to Goodbye-likes. In a plant homolog of HeLo, the N-terminal helix of the bundle (and entire protein) is known to play a significant role in triggering the cell death process [82]. However, in BaMLKL and Goodbye-likes, the bundle is extended N-terminally by one or more helices, respectively. Thus, while the common evolutionary ancestry of HeLo-like and Goodbye-like is rather evident, the question of their functional similarity remains open. In particular, the functional role of the N-terminal extension of Goodbye-likes remains to be explored. For example, it can be speculated that the helices of the N-terminal extension in Goodbye-likes are displaced to enable the oligomerization process occur, possibly under the control of a non-amyloid mechanism involving domains associated with Goodbye-likes.

### A large fraction of the effector domains are involved in regulated cell death

With the limitation that the evolutionary relation of Goodbye-like domains does not necessarily imply functional similarity, it appears that a substantial fraction of the effector domains in both ascomycetes and basidiomycetes is predicted to control regulated cell death. Involvement in regulated cell death has been reported not only for the HeLo/MLKL group but also for the HET domain [107], the Patatin [23] domain and more indirectly for the SesB-like domain [38]. One needs to add to this list the amyloid signaling motifs that control separate downstream cell-death effector domains. Globally, it would appear of at least one-third to half of the fungal NLRs could be involved in some kind of regulated cell death process. This high proportion raises the question of whether some of the other domains (whether annotated or not) could also play a role in regulated cell death. For example, it was recently reported that genes encoding fungal NLRs with N-terminal CHAT and S8 protease effector domains reside adjacent to Gasdermin-encoding genes [53].

### Annotation of very short domains requires more complex methods than profile HMMs

While the vast majority of longer domains is at least partially annotated, this is true only for a definite minority of shorter domains. The shortage of annotations cannot be easily explained by the lack of conserved sequential features. Instead, one of the reasons is the profile HMM model itself, which by assessing each alignment position independently (except for indels) is not statistically powerful enough when dealing with short sequences. In other words, profile HMM models of more diverse families of short sequence fragments (e.g. 20–40 amino-acids long) cannot be sensitive and specific at the same time [52]. Currently, the problem can be at least partially addressed by using more complex and computationally demanding protein sequence models, such as probabilistic context-free grammars (PCFG) [12, 52, 108] and co-evolutionary Potts models [109–111]. Another viable option are the recurrent and attention-based neural networks, which have enough computational power to describe relevant dependencies in protein sequences [112–114]. However, while modern neural networks have been successfully applied to annotation of protein families [115, 116], their performance in modeling short protein sequence fragments is yet too be evaluated.

### NLR-associated amyloid-like motifs are less diverse in fungi compared to bacteria

In Ascomycota, we discovered two new amyloid signaling motif family, which are uniquely associated with the PNP_UDP domain. The amyloid properties of the more abundant PUASM motif were confirmed experimentally using a representative pair of N- and C-terminal sequences. Both of them generated amyloid-like fibers in the *in vitro* condition. (In depth study of the co-aggregation process is left for a separate study.) The effector-side PUASM sequence was shown to be capable of forming prions *in vivo* in the *Podospora anserina* model. Despite the extensive search, the expand diversity of ASM remains lower in fungi than in bacteria using similar identification procedure, which is not inconsistent of the larger phylogenetic breath of the scanned bacterial genome as compared to the fungal ensemble.

### Two strategies emerge for facilitating inheritance of amyloid signaling

Similarly to other ascomycotal amyloid signaling motifs, HRAM, PP and *σ*, effector proteins with C-terminal motif are often coded by direct genomic neighbors of the motif–NLR genes. Such genomic co-localization may facilitate co-inheritence of the two genes of the functional unit in the event of a recombination process. This may be of special importance for the NLR signaling pathway, which is polymorphic in population given the death-and-birth evolution. A notable exception is a PNP_UDP-associated HRAM motif variant (NLR12), for which only in a few cases the effector–motif and motif–NLR pairs present in the genome were co-localized (that is encoded by adjacent genes), while its NLR-side instances were relatively more frequent than the effector-side instances in some genomes. In Basidiomycota, virtually none of the hundreds of instances of amyloid-like motifs found in our survey in N-termini of NLRs was genomically co-localized with amyloid-like motif instances in effector C-termini. Again, in genomes were ASM co-occurred in both types of proteins, the NLR-side N-terminal instances were more frequent than the effector-side C-terminal instances. We speculate that the presence of many NLRs controlling the same effector could potentially relieve the need for genomic co-localization of NLRs and their effectors linked by amyloid signaling sequences.

### Internal ASM instances may serve as scaffolds to stabilize the NLR oligomers

One interesting finding is presence of NLRs with intra-proteins amyloid-like motifs in *Fibularhizoctonia* sp. CBS 109695. Different central and C-terminal domain association in comparison to NLRs with N-terminal ASM-likes suggest also different functions of the motifs in both cases. Therefore, we hypothesize that these internal ASM instances may serve as scaffolds to stabilize the NLR oligomers, similar to cRHIM in the RIP1K/RIP3K complex [57]. In these lines, it is possible that also some other amyloid-like sequences identified in the current study but with no matching effector-side counterparts participate in the assembly of the NLR signalosome or are involved in interactions with motifs located outside the C-terminus of the associated protein.

## Materials and methods

### Computational methods

#### Annotation of NLR N-termini

A set of 36,141 NLR proteins from 487 fungal strains was identified in a previous study through the PSI-BLAST [117] search among completely sequenced fungal genomes in the NCBI nr database [11, 12] (the full list of accessions with their corresponding NOD domain boundaries is included in S1 Table). 32 962 N-termini at least 20 amino-acids long (91%), delimited according to the NACHT or NB-ARC query matches, were further considered, of which 18,674 (57%) were annotated using direct matches to Pfam [29] or inhouse HMM profiles (S1 Data) [11, 12]. The set of N-termini at least 20 amino-acid long was clustered with MMseqs2 [58] in mode 1 (21 758 N-termini in 127 clusters, 15 105 already annotated). Then, sequences in each cluster with at least 20 members were aligned using Clustal-Omega [118] (S2 Data) and searched for homologs in UniRef30 [59, 60] using HHblits [61] (parameters: -e 0.001 -n 2 -E 0.01 -Z 1000000 -M 50). Subsequently, the resulting alignments were used to search Pfam (HHblits parameters: -e 0.001 -n 1 -E 1 -Z 1000000). The clustering required mutual coverage of at least 80% of sequence length, and the annotations were only assigned to sequences which covered at least 50% of the match to the Pfam profile. The resulting cluster-level annotations were retained only if the alignment match to the Pfam profile covered at least 50% of the profile length, and assigned only to individual sequences which covered at least 50% of the match. After completing the main processing, the set of N-termini was re-scanned for the Crinkler domain (PF20147) added recently to the Pfam database.

The tabularized results of the annotation are provided in S1 Table. The overlapping Pfam annotations were resolved as in [11, 12]. The double HeLo/HeLo-like annotations were kept in S1 Table and in Table A in S1 Text but were represented as HeLo in Fig 1f and Fig B in S1 Text. In addition, basidiomycotal sequences from clusters doubly annotated as Goodbye-like/Helo-like, as well as from clusters with CDD [73] MLKL_NTD annotations, were denoted as BaMLKL (see Results).

#### Comparative analysis of Goodbye-, HeLo- and MLKL-likes

For the largest clusters annotated as HeLo, HeLo-like, Goodbye-like and BaMLKL, their representative sequences were submitted to AlphaFold2 structure prediction [77] through the ColabFold advanced notebook [78]. Standard parameters of the notebook were applied except of (1) using the cluster alignments instead of searching genetic databases, (2) trimming off fragments just upstream the NACHT domain were applicable. Successful models—with the mean predicted pLDDT score [77] above 70 overall, and around 80 or more for the core helix bundle—and respective ColabFold outputs are provided in S3 Data. For each cluster, the highest rank model was selected and structurally aligned to the experimentally solved MLKL domain (pdb:6zvo) using TM-align with default parameters [79]. Alignment conservation scores were calculated using the ConSurf webserver with default parameters [80, 81] based on the cluster alignments and AlphaFold2 structural models.

#### Characterization of unannotated longer N-termini

In addition, the largest MMSeqs-produced clusters, which did not get any Pfam annotation through the HHblits procedure, were carefully examined. For five unannotated clusters with at least ten members at the identity threshold of 70% and the median length above 100 amino acids, homologs were searched in UniProt [119] through the web-based hmmsearch with standard parameters [120], and predictions of the three dimensional structure for their representative sequences were attempted using AlphaFold2 [77] through the ColabFold advanced notebook [78]. Standard parameters were used except of adding the MMseqs2 alignments to input (sequences just upstream the NACHT domain was trimmed off). Good quality structures (the predicted pLDDT score above 70) were obtained for three clusters, KEY84097, KFH66451 and PQE30996 (S4 Data). The proposed annotations for the five clusters (Table B in S1 Text) are assigned to member sequences in S1 Table and included in the TIR-like and “other” groups in Fig 1f and Fig B in S1 Text.

#### Extraction of amyloid-like motifs in short N-termini

A subset of 54 NLR N-termini clusters with mean/median sequence length of at most 160/161 amino acids was selected. It consisted of N-termini of 3441 sequences, which were scanned using the PCFG-CM software [52, 121] probabilistic grammatical model inferred from ten families of bacterial ASMs (BASS1–10) [12, 52] (S5 Data) with scanning window of 20 to 40 amino acids and the smoothing factor of 10 PAM [52]. Very high scoring fragments (maximum log10 score at least 3.5, mean log10 score above 1.67) were found in 18 clusters with 1456 sequences (S6 Data). This included all 8 clusters (592 sequences) with at least one PFD-like annotation. The N-terminal sequences were made non-redundant at the identity level of 90% using CD-HIT 4.7 [122, 123] and submitted to motif extraction with MEME 5.0.5 [88, 124] with the following parameters: -nmotifs 100, -minsites 10, -maxsites 500, -minw 10, -maxw 30, -allw, -evt 1. For each of 51 motifs found at the E-value threshold of 1, HMM profiles were built with HMMER 3.2.1 [125] and used for searching against the full set of grammar-fitting N-terminals (at the sequence and domain E-values of 1*e* − 2). Then, obtained hits were extended by 5 amino acids in each direction and realigned using Clustal-Omega with the auto parameter. For each motif, the extended sequences were re-examined for consistency with the grammatical model (maximum log10 score at least 3, mean log10 score above 1). For 16 motifs which passed the grammatical filter, the alignments were used to build final HMM profiles (S7 Data).

#### Analysis of N-terminal amyloid-like motifs

The HMM profiles of the 16 motifs were used for scanning all N-termini longer than 10 amino acids (domain (independent) E-value threshold of 1*e* − 2), comprising also sequences not included in the 127 clusters with 20 or more members. The resulting hits in 1538 sequences are included in S1 Table with coordinates (outermost in rare cases of double ASM hits). For further analysis only hits in N-termini shorter than 200 amino acids not located beyond position 150 were considered. Motif sequences in envelopes of 5 amino acids were tested for the beta-arch structure with ArchCandy 2.0 [51] using the recommended threshold of 0.56. Constituent sequences of the motifs were scanned using a generalized HRAM profile (S8 Data) at the domain (independent) E-value of 1*e* − 2. The profile was built from HRAM motif sequences in Supplementary File 2 from [45], realigned using Mafft [126] (in the auto mode) and pruned of columns with more than 50% gaps using trimAl [127].

For each motif-containing NLR sequence, proteins coded by genes within the ±20kbp neighborhood of the genes encoding these NLRs were fetched from NCBI GenBank [128] or EMBL ENA [129] using an in-house Python (version 3.7.3) script aided by packages requests [130] and xmltodict [131] (S2 Table). The set was then confined to proteins in the length range of 200–400 amino acids (S9 Data), which is typical for proteins with single domain architectures known to be associated to NLRs via amyloid signaling [12, 45]. Next, C-termini (100 amino acids) of the found neighboring proteins were scanned for the presence of the motifs using HMMER (domain (independent) E-value threshold of 1*e* − 2, all heuristic filters off). Pairwise hits of the same motifs in N-termini of NLRs and C-termini of genomically neighboring proteins are collected in S3 Table.

Note that common occurrence of amyloid motifs at the N-termini of NLRs and at the C-termini of effector domains enco ded by adjacent genes was repeatedly used for the identification of such motifs both in fungi and bacterial genomes [12, 37]. This criterion adds sensitivity and specificity to the identification of amyloid motifs.

#### Homology search of effector domains

Remote homologs of effector domains related to NLR proteins were iteratively searched for, starting from 19 Pfam profiles of N-terminal domains of NLRs reported in [5]: Pkinase (PF00069), Peptidase_S8 (PF00082), C2 (PF00168), PNP_UDP_1 (PF01048), TIR (PF01582), Patatin (PF01734), RelA_SpoT (PF04607), DUF676 (PF05057), HET (PF06985), PK_Tyr_Ser-Thr (PF07714), PGAP1 (PF07819), Abhydrolase_6 (PF12697), CHAT (PF12770), TIR_2 (PF13676), HeLo (PF14479), NACHT_N (PF17100), SesA (PF17107), Goodbye (PF17109) and Helo_like_N (PF17111). First, Pfam HMM profiles for each of the domains were used for searching against a local copy of the non-redundant protein sequences database (NCBI’s “nr”, downloaded in November 2019) [128] using HMMER 3.2.1 with the sequence inclusion E-value of 1*e* − 2. Found proteins were then used to build the new HMM profiles and the search was repeated (this time with the more stringent sequence inclusion E-value of 1*e* − 3) until the number of hits did not change by more than 7%. Final profiles were used to delimit domain boundaries through yet another hmmsearch run with the same E-value parameter but all heuristic filters turned off and the initial search space set to 12 155 478 (S10 Data). In addition, all fungal NACHT (PF05729) and NB-ARC (PF00931) proteins were retrieved from the Pfam database (as of January 2020). C-termini of effector domains and N-termini of NACHT/NB-ARC NLRs were extracted and—in both cases—only fragments between 10 and 150 aa were selected for further analysis. (This effectively excluded nearly all proteins with effector + NOD architectures.) The final set included around 235k (nr: 187k) of effector C-termini and 6.8k (nr: 5.1k) NLR N-termini (S11 and S12 Data, respectively).

#### Identification of paired amyloid motifs

The sets of N- and C-termini were clustered using CD-HIT to reduce redundancy at the 70% similarity threshold (separately for each effector domain, together for NACHT and NB-ARC). Then, motif search was performed using MEME with the following parameters: -nmotifs 100 for effectors or -nmotifs 50 for NLRs, -minsites 1% of sequences but no less than 5 and no more than 10, -maxsites 500, -minw 10, -maxw 30, -mod anr. For each of 818 motifs identified at the E-value threshold of 1, including 769 motifs in effector C-termini and 49 motifs in NLR N-termini, HMM profiles were built in the two-stage procedure, as described above (see S13 Data). Next, the N- and C-termini were scanned with the combined set of effector- and NLR-side motif profiles. The same-motif hits in effector proteins and in NLRs (at domain (independent) E-value of 1*e* − 2) were matched based on genomic proximity (up to 20kbp) of genes encoding the proteins (see S4 Table for the genomic neighborhoods of genes encoding NACHT and NB-ARC proteins with short N-termini). At least 3 non-redundant pairs of motif instances were found for 22 motifs (S13 Data), which were then clustered on the basis of their co-occurrence in 190 pairs of genomically neighboring proteins (S5 Table and Fig E in S1 Text).

Finally, hits of the 16 ASM motif profiles in short N-termini of NLRs (previously analyzed) and hits in short C-termini of effector domains (from the homology search, included at domain (independent) E-value of 1*e* − 2 over the entire set) were matched on the strain level (through the BioSample and BioProject identifiers; entries with incomplete pairs of identifiers were rejected) in order to identify potentially correlated pairs, which are not co-localized in genomes (S6 Table).

#### Specialized searches for amyloid motifs in Basidiomycota

BaMLKL homologs were searched in UniProt [119] through the web-based hmmsearch [120] with standard parameters starting from the alignment of the largest BaMLKL cluster in Basidiomycota (representative protein: KIM77258), trimmed to the NACHT_N match. Hits were further restricted to GenBank sequences with length up to 400 amino acids and no Pfam P-loop_NTpase clan (CL0023) annotation at E-value of 1. C-termini (100aa) of resulting 241 BaMLKL homologs (S14 Data) were scanned with the PCFG BASS model (S5 Data) with the same parameters as above (except the minimum scanning window length of 15). For proteomes with the most promising hits in BaMLKL homologs (log10 score above 3, eight sequences from six species), N-termini (150 aa) of all NLR proteins were again scanned with the grammars. Promising N-terminal hits were obtained for *Moniliophthora roreri* (strains 2995 and 2997), *Laccaria amethystina* (strain LaAM-08–1), and *Fibularhizoctonia* sp. CBS 109695. The matched fragments were aligned with their C-terminal counterparts on the per genome basis with Mafft [126] in an accurate mode (–maxiterate 1000 –localpair). The NLR N-terminal and BaMLKL C-terminal ASM-like sequences aligned satisfactorily for *M. roreri* (we only analyzed strain 2997 due to high similarity between the strains) and *Fibularhizoctonia* sp. CBS 109695. The alignments were then extended and trimmed manually (Fig F and Fig G in S1 Text). In addition, the sequences were scanned with the 16 HMM profiles of amyloid-like motifs (domain (independent) E-value of 1*e* − 2).

Next, fungal proteomes in UniProt were scanned using web-based jackhmmer [120] with standard parameters starting from the double HET-s motif from Q03689 (AAB94631) (residues 218–289) of *Podospora anserina*, which resulted in finding five complete HeLo-HRAM-HRAM proteins in two Agaricomycetes: four from *Sphaerobolus stellatus* SS14 and one from *Gymnopus luxurians* FD-317 M1 (see Fig 4). NLRs in these genomes were then scanned with the PCFG model and the hits exceeding the log10 score threshold of 2.33 were aligned with their C-terminal counterparts on the per genome basis with Mafft [126] in the accurate mode. Finally, the alignments were curated manually (poorly aligned sequences were excluded, sequences were extended or trimmed if necessary, Fig H and Fig I in S1 Text).

#### Visualization

Basic data processing and visualization was conducted in Python using pandas [132, 133], matplotlib [134] and seaborn [135] packages, as well as in LibreOffice, GIMP and Inkscape. Multiple sequence alignments and logos were generated using TeXshade [136]. The graph of logos in Fig E in S1 Text was generated with graphviz 2.40.1 [137]. Visualizations of structural models were generated with RasMol [138] (Fig 2) or taken directly from the ColabFold notebook [78] (Fig D in S1 Text).

### Experimental methods

#### In vitro analysis

**Peptide synthesis**. All commercially available reagents and solvents were purchased from Merck, Sigma-Aldrich and Lipopharm.pl, and used without further purification. Peptides EQB50682.1_332_355 (VFHGKGIQHTGSGNFSVGNDLSIS) and EQB50683.1_9_31 (FHGHGIALSGAGNITVGGDFIIG) were synthesized with an automated solid-phase peptide synthesizer (Liberty Blue, CEM) using rink amide AM resin (loading: 0.59 mmol/g). Fmoc deprotection was achieved using 20% piperidine in DMF for 1 min at 90°C. A double-coupling procedure was performed with 0.5 M solution of DIC and 0.25 M solution of OXYMA (1:1) in DMF for 4 min at 90°C. Cleavage of the peptides from the resin was accomplished with the mixture of TFA/TIS/H_2_O (95:2.5:2.5) after 3 h of shaking. The crude peptide was precipitated with ice-cold Et_2_O and centrifuged (8000 rpm, 15 min, 2°C). Peptides were purified using preparative HPLC (Knauer Prep) with a C18 column (Thermo Scientific, Hypersil Gold 12 *μ*l, 250 × 20 mm) with water/acetonitrile (0.05% TFA) eluent system.

**Peptide analytics**. Analytical high-performance liquid chromatography (HPLC) was performed using Kinetex 5*μ* EVO C18 100A 150 × 4.6 mm column. Program (eluent A: 0.05% TFA in H_2_O, eluent B: 0.05% TFA in acetonitrile, flow 0.5 mL/min): A: t = 0 min, 90% A; t = 45 min (25 min in case of EQB50682.1_332_355). Peptides were studied by WATERS LCT Premier XE System consisting of high resolution mass spectrometer (MS) with a time of flight (TOF).

**Attenuated Total Reflectance—Fourier Transform Infrared Spectroscopy (ATR-FTIR)**. Lyophilized peptides were dissolved in D_2_O (deuterium oxide, 99.8% D, Carl Roth, GmbH, Germany) to final concentration of ca. 814 *μ*M. The spectroscopic measurements were performed directly after dissolving peptides in a solvent, after 7 and 40 days of incubation process at 37°C (98.6°F). In addition peptides were measured after 40 days of incubation at 4°C (39.2°F, Fig K in S1 Text). Each time, 10 *μ*l of peptide solution was dropped directly on the diamond surface and was allowed to dry out. ATR-FTIR spectra were recorded using a Nicolet 6700 FTIR Spectrometer (Thermo Scientific, USA) with Golden Gate Mk II ATR Accessory with Heated Diamond Top-plate (PIKE Technologies). The spectrometer was continuously purged with dry air. Directly before sampling, the background spectrum of diamond/air was collected as a reference. For each spectrum 512 scans with a resolution of 4 cm^−1^ were co-added. All spectra were obtained in the range of 4000–450 cm^−1^ at 20°C (68.0°F).

**Spectroscopy data treatment**. ATR-FTIR spectra were initially preprocessed using OMNIC software (version 8, Thermo Fisher Scientific, USA): atmospheric and ATR correction. All spectra were analyzed using the OriginPro (version 2019, OriginLab Corporation, USA). The analysis included: baseline correction, smoothing using the Savitzky-Golay polynomial filter (polynomial order 2, a window size of 9 points) [139] and normalization to 1 for the Amide II’ band. Spectra in the amide bands region (1750–1500 cm^−1^) were deconvoluted into subcomponents using the Lorentz function based on second and fourth derivative spectra (R-Square 0.997).

**Atomic Force Microscopy**. AFM images were acquired in tapping mode using a Nanoscope IIId scanning probe microscope with Extender Module (Bruker) in the dynamic modus. An active vibration isolation platform was applied. Olympus etched silicon cantilevers were used with a typical resonance frequency in the range of 100–200 kHz and a spring constant of 40 N/m. The set-point amplitude of the cantilever was maintained by the feedback circuitry at 80% of the free oscillation amplitude of the cantilever. The volume of 10 *μ*L of 0.814 *μ*M peptide was applied to freshly cleaved ultra-clean mica (Nano and More) and incubated at room temperature for 30 s. The mica discs were then rinsed with ultra-clean purified 18.2 MΩ deionized water and dried using gentle nitrogen gas flow. All samples were measured at room temperature in air. Structural analysis and height measurements of acquired images were performed with Nanoscope v.6.13 software.

**Thioflavin T fluorescence assay**. ThT powder was dissolved in MilliQ to final concentration 2 mM and filtered through 0.22 *μ*m syringe. ThT solution was dissolved in 50 mM Tris-HCl (pH = 7.4) to final concentration 10 *μ*M and filtered. The 90 *μ*L of ThT buffer was mixed with 10 *μ*L of peptide solution (concentration 400 *μ*M) in the 96-wells plate. Samples were measured on the SpectraMax Gemini XPS Microplate (Molecular Devices LLC). The measurements were conducted in room temperature. The excitation wavelength was set at 450 nm and the emission was recorded in the range from 470 to 500 nm. Each group of experiment contained three parallel samples and the data were averaged after measurements.

#### In vivo analysis

**Strains and plasmids**. The *Podospora anserina* Δhellp (ΔPa_5_8070) Δhet-s (ΔPa_3_620) Δhellf (ΔPa_3_9900) strain [106] was used as recipient strain for the expression of molecular fusions of PUASM (PNP_UDP-side C-terminal EQB50682.1_332_355 VFHGKGIQHTGSGNFSVGNDLSIS) from the plant pathogenic fungus *Colletotrichum gloeosporioides* Cg-14 [89] and the GFP (green fluorescent protein) or RFP (red fluorescent protein). These fusions were expressed from plasmids based on the pGEM-T backbone (Promega) named pOP [38] and containing either the GFP or RFP encoding gene, or in a derivative of the pAN52.1 GFP vector [140], named pGB6-GFP and containing the GFP encoding gene. In both cases, the molecular fusions were under the control of the strong constitutive *P. anserina* gpd (glyceraldehyde-3-phosphate dehydrogenase) promoter. The Δhellp Δhet-s Δhellf strain was transformed as described [141] with a fusion construct along with a second vector carrying a ble phleomycin-resistance gene, pPaBle (using a 10:1 molar ratio). Phleomycin-resistant transformants were selected, grown for 30 h at 26°C and screened for the expression of the transgenes using fluorescence microscopy. PUASM was amplified with specific primers either 5’ ggcttaattaaATGGTCTTTCATGGCAAGGGCATCC 3’ and 5’ ggcagatcttgctccGGAGATGCTGAGATCG 3’ for cloning in pOP plasmids, or 5’ ggcgcggccgcGTCTTTCATGGCAAGGGCATC 3’ and 5’ ggcGGATC-CTTAGGAGATGCTGAGATCGTTGCC 3’ for cloning in the pGB6 plasmid (capital letters correspond to the PUASM sequence). The PCR products were cloned upstream of the GFP or RFP coding sequence in the pOP plasmids using PacI/BglII restriction enzymes to generate the pOPPUASM-GFP and pOPPUASM-RFP vectors in which in addition to the BglII site, a two amino acid linker (GA) was introduced between the sequences encoding PUASM and GFP or RFP and cloned downstream of the GFP using NotI/BamHI restriction enzymes to generate the pGB6-GFP-PUASM plasmid.

**Microscopy**. *P. anserina* hyphæ were inoculated on solid medium and cultivated for 24 to 48 h at 26°C. The medium was then cut out, placed on a glass slide and examined with a Leica DMRXA microscope equipped with a Micromax CCD (Princeton Instruments) controlled by the Metamorph 5.06 software (Roper Scientific). The microscope was fitted with a Leica PL APO 63X immersion lens.

**Prion propagation**. Methods for determination of prion formation and propagation were previously described [12, 142]. Prion formation and propagation can be observed using microscopy by monitoring the formation of fluorescent dots. Spontaneous prion formation is first monitored as the rate of spontaneously acquired prion phenotype (dot formation) in the initially prion-free subculture after 5, 11, 18, 32, 49 and 75 days of growth at 26°C on corn-meal agar using microscopy as described. Prion formation can also be measured as the ability to propagate prions from a donor strain (containing prion) to a prion-free strain (induced strain). In practice, prion-free strains are confronted on solid corn-meal agar medium for 2 to 5 days (contact between strains was observed after 24 to 36 hours of culture) before being subcultured and observed by fluorescence microscopy for the presence of dots (this test is referred to as induced prion formation). At least 18 different transformants were used and the tests were realized in triplicates. It is to note that transformants were randomly tested for prion formation allowing various expression levels of the transgene (high levels of expression are usually associated with rapid spontaneous prion formation) except for the induced conversion test where transformants expressing moderate level of transgene were preferred to limit the rate of spontaneous transition within the timing of the experiment that could mask the prion induction.

As a control, we also imaged anew GFP fusion proteins with the wild-type and mutant form of a previously characterized amyloid signaling motif the BASS3 motif found in WP_037701008.1 from *Streptomyces atratus* described in [12]. Two proline mutants substituting conserved glutamine residues that were found previously to abolish in vivo dot formation were used (Q113P and Q120P).

## Supporting information

S1 Text Supplementary online materials. The document includes supplementary text, tables (Table A–F in S1 Text) and figures (Fig A–L in S1 Text).
(PDF)

S1 Table Tabularized results of N-termini annotation. The table aggregates results presented in the manuscript.
(CSV)

S2 Table Genomic neighbors of candidate short N-termini NLRs with ASMs. The list includes accessions of proteins encoded by genes within the neighborhood of 20kbp of genes encoding the query proteins (S6 Data).
(CSV)

S3 Table Pairwise hits of the same ASMs in N-termini of NLRs and C-termini of genomically neighboring proteins. The table is based on S6 and S7 Data, S2 Table and S9 Data. See Computational methods for details.
(CSV)

S4 Table Genomic neighbors of candidate short N-termini Pfam NACHT and NB-ARC proteins. The list includes accessions of proteins encoded by genes within the neighborhood of 20kbp of genes encoding the query proteins (S12 Data).
(CSV)

S5 Table Pairwise hits of the same ASMs in N-termini of NACHT/NB-ARC NLRs and C-termini of genomically neighboring effector proteins. The table is based on S11, S12 and S13 Data and S4 Table. See Computational methods for details.
(CSV)

S6 Table Pairwise hits of the same ASMs in N-termini of NLRs and C-termini of genomically co-occurring effector proteins. The table is based on S6 and S7 Data and S11 Data. See Computational methods for details.
(CSV)

S1 Data Profile HMMs of NLR effector domains. The file includes previously unpublished models used in [5, 11].
(HMM)

S2 Data Multiple sequence alignments of N-termini clusters. The alignments were calculated using ClustalOmega for 127 MMseqs2 clusters with at least 20 member sequences.
(GZ) Click here for additional data file.

S3 Data Structure prediction of HeLo-/Goodbye-/MLKL-like domains. Full AlphaFold2/ColabFold outputs.
(GZ) Click here for additional data file.

S4 Data Structure prediction of previously unannotated domains. Full AlphaFold2/ColabFold outputs.
(GZ) Click here for additional data file.

S5 Data PCFGs for BASS. The file includes previously unpublished grammars used in [52] and a sample scanning configuration.
(GZ) Click here for additional data file.

S6 Data Candidate short NLR N-termini with ASMs. The FASTA file includes sequences from clusters with high content of ASM-like sequences, according to the BASS PCFGs (S5 Data).
(FA) Click here for additional data file.

S7 Data Profile HMMs of ASMs found in short NLR N-termini. Please refer to Computational methods for the profile generation process.
(HMM)

S8 Data Profile HMM of HeLo-related HRAMs. The profile is based on the motifs identified in [45].
(HMM)

S9 Data Short C-termini of 200–400 aa long proteins genomically neighboring candidate short NLR N-termini with ASMs. The FASTA file concerns target proteins listed in S2 Table.
(FA) Click here for additional data file.

S10 Data Lists of HMMER domain hits of effector domain profiles. The lists were obtained through iterative searches in NCBI “nr” starting from Pfam profiles of known NLR effector domains.
(GZ) Click here for additional data file.

S11 Data Short C-termini of effector proteins. The FASTA file concerns target proteins listed in S10 Data.
(FA) Click here for additional data file.

S12 Data Short N-termini of Pfam NACHT and NB-ARC proteins. The FASTA file concerns proteins from NCBI “nr” associated with the two families in the Pfam database.
(FA) Click here for additional data file.

S13 Data Profile HMMs of ASMs found both in effector C-termini and NLR N-termini of genomically neighboring proteins. Please refer to Computational methods for the profile generation process.
(HMM)

S14 Data BaMLKL homologs identified with hmmsearch in Basidiomycota. A FASTA file.
(FA) Click here for additional data file.
