# Supplementary material for: Exploring a diverse world of effector domains and amyloid signaling motifs in fungal NLR proteins
Source: PLoS Comput Biol. 2022 Dec 21;18(12):e1010787. doi: 10.1371/journal.pcbi.1010787 (PMC9815663; doi:10.1371/journal.pcbi.1010787)
Supplement: S1 Text — The document includes supplementary text, tables (Table A–F in S1 Text) and figures (Fig A–L in S1 Text). (PDF) [file pcbi.1010787.s001.pdf]

Exploring a diverse world of effector domains and amyloid signaling motifs in fungal NLR proteins

Jakub W. Wojciechowski[‡,1], Emirhan Tekoglu[‡,§,2,3], Marlena Gąsior-Głogowska[1], Virginie Coustou[4], Natalia Szulc[1], Monika Szefczyk[5], Marta Kopaczyńska[1], Sven J. Saupe[*,4], and Witold Dyrka[*,1]

[1]Katedra Inżynierii Biomedycznej, Wydział Podstawowych Problemów Techniki, Politechnika Wrocławska, Wrocław, Poland
[2]Biyomühendislik Bölümü, Yıldız Teknik Üniversitesi, İstanbul, Turkey
[3]Wydział Chemiczny, Politechnika Wrocławska, Poland
[4]Institut de Biochimie et de Génétique Cellulaire, UMR 5095 CNRS, Université de Bordeaux, Bordeaux, France
[5]Katedra Chemii Bioorganicznej, Wydział Chemiczny, Politechnika Wrocławska, Wrocław, Poland

‡ Contributed equally
§ Present address: Koc University, School of Medicine, Istanbul, Turkey
* witold.dyrka@pwr.edu.pl or sven.saupe@ibgc.cnrs.fr

Results

Super-architectures of NLR N-termini. The corpus of fungal NLR N-termini can be broadly divided into four main super-architectures (Fig 1a). The first of them consists of the shortest N-termini, up to roughly 50 amino acid in length, which mainly comprise a direct N-terminal extension of the nucleotide binding domain. The second architecture adds the amyloid signaling motifs (ASM), typically made of 20–30 residues, which makes the entire N-terminus 70–120 amino-acids long. The third and the most frequent architecture consists of a single effector domain, while the fourth comprises with multiple domains.

Additional notes on the updated annotations of NLR N-termini. Pfam annotations for fungal NLR N-termini are listed in Table A and summarized in Fig 1f and Fig B. The Crinkler domain of the Ubiquitin was found in two mycorrhizal species from two different phyla: *Serendipitia vermifiera* (Basidiomycota) and *Rhizophagus irregularis* (Mucormycota). No MMSeqs-produced cluster matched the previously reported RelA_SpoT [1], PKinase [2], CHAT [3] and TIR [4, 5]

profiles with sufficiently high hit coverage. Interestingly, in the profile-sequence search PKinase was found to be N-terminally attached to another N-terminal domain in 29 cases, therefore it is not unlikely that the remaining single PKinase hits are also partial annotations. While no cluster was annotated as C2 only [6], a huge number of sequences fell into the C2 Goodbye-like class [7, 8] (356 hits), the architecture which is specific to Agaricomycetes. While the clustering-based approach found less SesB-like annotations than the profile-sequence search, this is likely due to the fact that the former procedure used only Pfam AB_Hydrolase clan [9] entries, while the latter method also relied on a sensitive inhouse SesB-like profile [8, 10].

Detailed analyses of the unannotated longer N-termini. Roughly half of the 127 clusters with at least 20 member sequences did not get any Pfam annotation through the HHblits procedure. We carefully examined clusters with at least 10 non-redundant sequences (identity threshold of 70% or nr70) and median length above 100 amino acids. Apart from the MLKL-related clusters described above, there were five such clusters with a total of 195 sequences (127 at nr70) corresponding to four candidate domain families (Table B). All five clusters were associated with NACHT NLRs.

Two clusters (with a total of 94 sequences) consisted of homologous (HHalign [11] E-value of $6e-44$) relatively long domains (N-terminal length above 500 amino acids) from Pezizomycotina (e.g. KEY84097 protein from *Aspergillus fumigatus*) and Mortierellomycetes (e.g KFH66451 from *Podila verticillata*). Structure prediction with AlphaFold2 [12, 13] indicates the domain is made of multiple alpha-helices forming two stretches of the alpha solenoid-like structure (Fig Dab). NLRs with this N-terminal domain usually display C-terminal repeats of type HEAT (from the TPR clan) in Ascomycota, and WD40 in Mucormycota. Homologous proteins were also found (through the web-based profile HMM search) in bacteria, mainly in *Mycoavidus cysteinexigens*. As this betaproteobacteria is an endosymbiont of *Linnemania (Mortierella) elongata* AG-77, a fungus with the largest number of these proteins [14], this may suggest possibility of the horizontal gene transfer.

The second most abundant unannoted cluster included 41 sequences (32 non-redundant at nr70) from *Agaricus bisporus* and *Leucoagaricus sp. SymC.cos* (representative protein XP_007333708 from *A. bisporus*), yet the profile HMM search revealed also single homologs in *Coprinopsis marcescibilis*, yeast *Saprochaete ingens* and protozoan parasite *Eimeria burnetti*. Interestingly, the latter species is a host of *Totiviridae* RNA virus similar to fungal viruses [15] reported also in Agaricaceae [16, 17]. These NLR N-termini (median length of 246 amino acids) consist of proline-rich disordered region and stretches of amino-acid composition resembling known ASMs, including a conserved motif in C-terminus. The yeast homolog (non-NLR) is partially annotated as the glycogen recognition site of AMP-activated protein kinase AMPK1_CBM (PF16561) [18]. Due to the central disordered part, poorly alignable, no reliable structure prediction was possible.

Third most populated unannotated cluster consisted of 31 sequences (15 at nr70, representative protein KIL58680) with the median length of 210 amino acids, which are specific to the *Amanita muscaria* strain Koide BX008. In NLRs the domain is typically associated with C-terminal WD repeats. Again, no reliable structure was predicted.

The final candidate effector domain identified in this study was found in 29 sequences (19 at nr70) from various Pezizomycotina species (representative protein PQE30996 from *Rutstroemia sp.* NJR-2017a WRK4). The N-terminus has the median length of 389 amino acids and partially resembles the SEFIR family ([19, 20]) of TIR clan (HHblits hit probability of 90%). The TIR domain was reported in NLRs from plants, bacteria and Chytridiomycota [21, 22, 10]. The NLR

proteins in this cluster are often associated with C-terminal repeats of Ankyrin, HEAT and WD40 types. In addition to the NACHT-based architectures present in the cluster, the web-based profile HMM search revealed several additional homologs (E-value $> 1e-52$, identity $> 30\%$ and similarity $> 70\%$ over the length > 200aa) in NLRs with the NB-ARC TPR domains. Interestingly, homologous domains are also present as separate proteins in Mucormycota *Rhizophagus irregularis*, a species related to *Mortierella*, and in association with NACHT WD40 and NACHT HEAT in *Mycoavidus cysteinexigens*, in accordance with the possibility of horizontal gene transfer [23]. A good quality structural model predicted with AlphaFold2 (pLDDT$>$ 80%) supports homology to TIR and HET domains (Fig Dc).

Tables

Table A: **N-terminal annotations of fungal NLRs.** Annotations based on Pfam [24] and inhouse [8] families and superfamilies. HHblits hits based on Pfam only. Direct hits include also inhouse annotations from [10]. Annotations are generally sorted by decreasing number of sequences. Homologous families are grouped together. Given are also the typical architecture of central and C-terminal domains if there is one clearly dominating, and the major taxonomic branch including vast majority of the hits.

Annotation	HHblits	Direct	Typical arch.	Major tax. branch
Goodbye-like	4912	4876	NACHT var.	Asco- & Basidiomycota
C2 Goodbye-like	356	55	NACHT WD	Agaricomycetes
PUP Goodbye-like	22	25	NACHT undef.	Eurotiomycetes
SesB-like Goodbye-like	20	18	NACHT ZF	Ascomycota
Goodbye-like/HeLo-like	130	—	NACHT undef.	Basidiomycota
HeLo-like	4044	4395	NACHT var.	Asco- & Basidiomycota
HeLo/HeLo-like	251	—	NACHT ANK	Ascomycota
HeLo	92	296	NACHT undef.	Ascomycota
SesB-like	3000	3851	var.	Ascomycota
PUP	3367	2647	var.	Ascomycota
C-terminus of PUP	45	—	NACHT var.	Ascomycota
HET	873	887	var.	Ascomycota
TIR	—	19	NACHT var.	Chytridiomycota
Patatin	646	651	NB-ARC TPR	Asco- & Basidiomycota
PFD-like	94	167	var.	Ascomycota
C2	—	137	NACHT WD	Agaricomycetes
RelA_SpoT	—	67	NACHT WD	Sordariomycetes
Crinkler	24	27	undef.	Glomeromycetes
SAM_Ste50p	24	—	undef.	Eurotiomycetes
Peptidase_S8	22	22	NACHT WD	Ascomycota
PKinase	—	22	NACHT var.	Ascomycota
CHAT	—	19	var.	Ascomycota

Table B: **Unannotated N-terminal domains of fungal NLRs.** For each cluster given are: an accession of its representative protein is given (Rep. acc.), proposed annotation label, number of sequences in cluster in total (#seq) and non-redundant at identity threshold of 70% (nr70), median sequence length (Len.), typical architecture of central and C-terminal domains (undef.—unannotated, var.—variable), and taxonomic distribution. NLR_PRDR stands for NLR effector domain with a Proline-Rich Disordered Region. See main text for details and Fig S2 for structural models of KEY84097, KFH66451 and PQE30996.

Rep. acc.	Prop. annot.	#seq (nr70)	Len.	Typical arch.	Tax. distrib.
KEY84097	NLR_Helical	67 (42)	515	NACHT TPR	Pezizomycotina
KFH66451	NLR_Helical	27 (19)	617	NACHT WD	Mortierellomycetes
XP_007333708	NLR_PRDR	41 (32)	246	NACHT undef.	Agaricaceae
KIL58680	NLR_Koide	31 (15)	210	NACHT WD	*Amanita muscaria*
PQE30996	TIR-like	29 (19)	389	NACHT var.	Pezizomycotina

Table C: **Analytical data for synthesized PUASM peptides.** Notations: M_{cal} — calculated mass of the peptide, M_{MS} — found mass of the peptide using HRMS, t_{ret} — retention time in analytical HPLC spectra (see Fig J)

Id/range	Formula	M_{cal}	M_{MS}	t_{ret}
EQB50682.1	$C_{107}H_{165}N_{33}O_{34}$	[(M+2H)/2] 1229.6191	[(M+2H)/2] 1229.6208	16.224
332 – 355		[(M+3H)/3] 820.0820	[(M+3H)/3] 820.0734	
EQB50683.1	$C_{100}H_{153}N_{29}O_{28}$	[(M+2H)/2] 1105.5813	[(M+2H)/2] 1105.5806	18.848
9 – 31		[(M+3H)/3] 737.3901	[(M+3H)/3] 737.393	

Table D: **Main Amide I' and Amide II'' band components and integrated intensities** obtained from the curve fitting procedure of ATR-FTIR spectra of air-dried PUASM peptide films with tentative secondary structures assignments. The results from experiments on the day of dissolving and incubation for 7 and 40 days at 37°C (98.6°F)

EQB50682.1_332_355							
after dissolving		after 7 days		after 40 days		band assignment	
band pos. [cm^{-1}]	area [%]	pos. [cm^{-1}]	area [%]	pos. [cm^{-1}]	area [%]	Amide	corresp. structure
1695	6	1691	6	1690	7	I'	β-sheets & β-turns
1677	16	1676	13	1673	17		β-sheets & β-turns
1663	19	1662	18	1658	16		β-sheets & β-turns
1648	14	1646	21	1642	16		α-helices & coils
**1631**	**14**	**1630**	**16**	**1625**	**18**		**β-sheets**
**1621**	**18**	**1619**	**21**	**1617**	**14**		**aggregated strands**
1554	6	1549	2	1550	6	II'	α-helices
1538	7	1535	3	1533	6		β-sheets & turns

EQB50683.1_9_31							
after dissolving		after 7 days		after 40 days		band assignment	
band pos. [cm^{-1}]	area [%]	pos. [cm^{-1}]	area [%]	pos. [cm^{-1}]	area [%]	Amide	corresp. structure
1694	3	1696	3	1693	5	I'	β-sheets & β-turns
1679	11	1679	7	1674	15		β-sheets & β-turns
1664	12	1667	12	1659	13		β-sheets & β-turns
1650	12	1651	23	1645	13		α-helices & coils
**1630**	**31**	**1628**	**26**	**1626**	**37**		**β-sheets**
**1616**	**14**	**1616**	**22**	**1611**	**11**		**aggregated strands**
1552	10	1552	4	1553	3	II'	α-helices
1535	7	1537	3	1538	3		β-sheets& turns

Table E: **Main Amide I' and Amide II' band components and integrated intensities** obtained from the curve fitting procedure of ATR-FTIR spectra of air-dried PUASM peptide films with tentative secondary structures assignments. The results from experiments on the day of dissolving and incubation for 40 days at 4°C (39.2°F)

EQB50682.1_332_355					
after dissolving		after 40 days		band assignment	
band pos. [cm^{-1}]	area [%]	pos. [cm^{-1}]	area [%]	Amide	corresp. structure
1695	6	1696	4	I'	β-sheets & β-turns
1677	16	1679	10		β-sheets & β-turns
1663	19	1665	14		β-sheets & β-turns
1648	14	1651	17		α-helices & coils
**1631**	**14**	**1632**	**13**		**β-sheets**
**1621**	**18**	**1620**	**27**		**aggregated strands**
1554	6	1552	8	II'	α-helices
1538	7	1536	7		β-sheets & turns

EQB50683.1_9_31					
after dissolving		after 40 days		band assignment	
band pos. [cm^{-1}]	area [%]	pos. [cm^{-1}]	area [%]	Amide	corresp. structure
1694	3	1695	3	I'	β-sheets & β-turns
1679	11	1676	10		β-sheets & β-turns
1664	12	1663	8	Amide I'	β-sheets & β-turns
1650	12	1650	12		α-helices & coils
**1630**	**31**	**1627**	**37**		**β-sheets**
**1616**	**14**	**1612**	**15**		**aggregated strands**
1552	10	1551	9	II'	α-helices
1535	7	1535	6		β-sheets & turns

Table F: **Spontaneous and induced prion formation rates of the PUASM motif.** Induction of prion formation was achieved by contact with strains expressing the prion state. "Diff" indicates a soluble state of the fusion protein; "foci" indicates an aggregated state appearing as foci in fluorescence microscopy. Number of transformants in a given state is indicated in columns "#", while percentage of transformants in the aggregated state – in columns "%".

		PUASM-GFP			PUASM-RFP			GFP-PUASM		
	duration	diff	foci		diff	foci		diff	foci	
		#	#	%	#	#	%	#	#	%
spontaneous prion formation (days after transfection)	5d	30	0	0	30	0	0	29	1	3
	11d	30	0	0	30	0	0	26	4	13
	18d	30	0	0	30	0	0	24	6	20
	32d	30	0	0	30	0	0	21	9	30
	49d	30	0	0	30	0	0	20	10	33
	75d	30	0	0	30	0	0	18	12	40
induced prion formation (hours after contact with prion donor)	12h	18	0	0	18	0	0	10	8	44
	48h	18	0	0	18	0	0	7	11	61
	96h	18	0	0	18	0	0	0	18	100

Figures

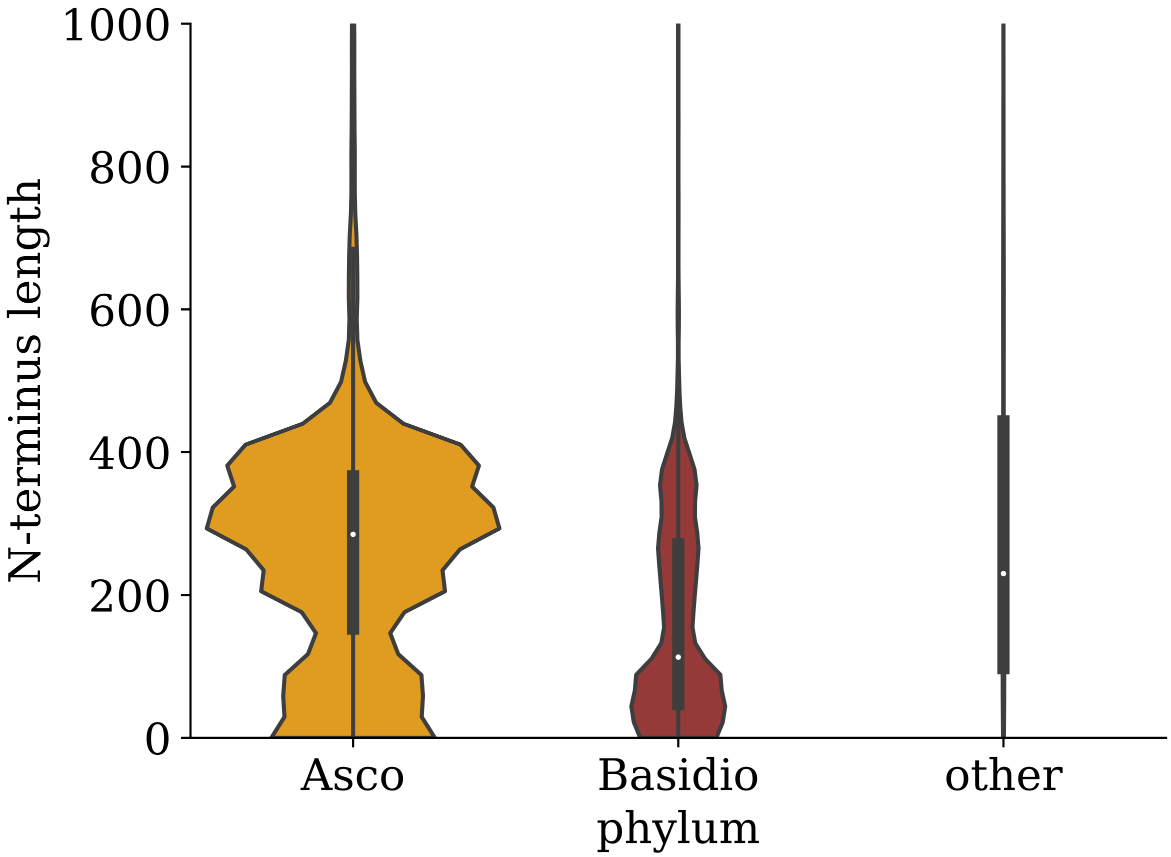

Figure A: **N-termini length distribution** with regard to phylum. Plots are truncated at the length of 1000 amino acids. Violin area is proportional to the number of sequences in phylum. Inner box plots show the quartiles of the distributions.

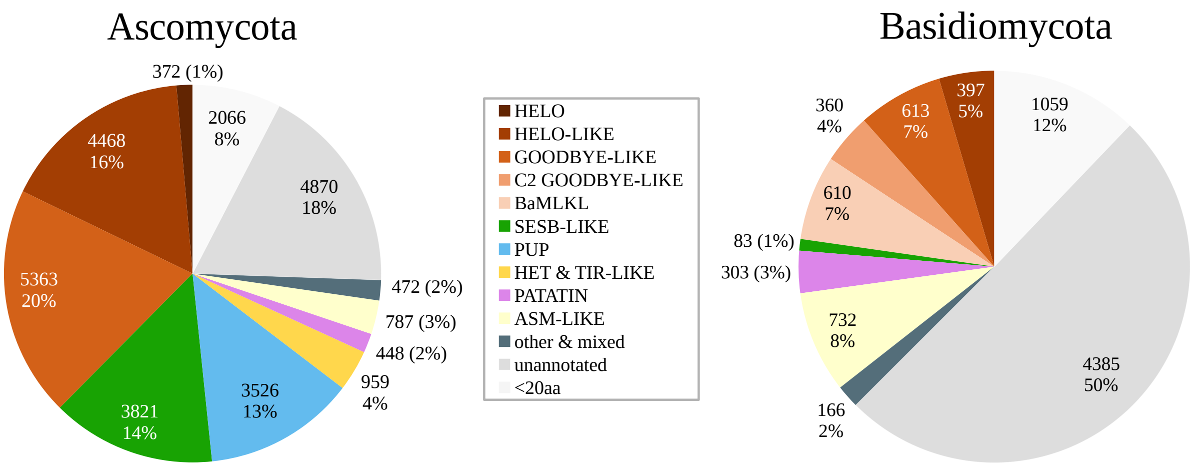

Figure B: **Distribution of domain families in fungal NLR N-termini** with regard to phylum. See Results and Methods for details.

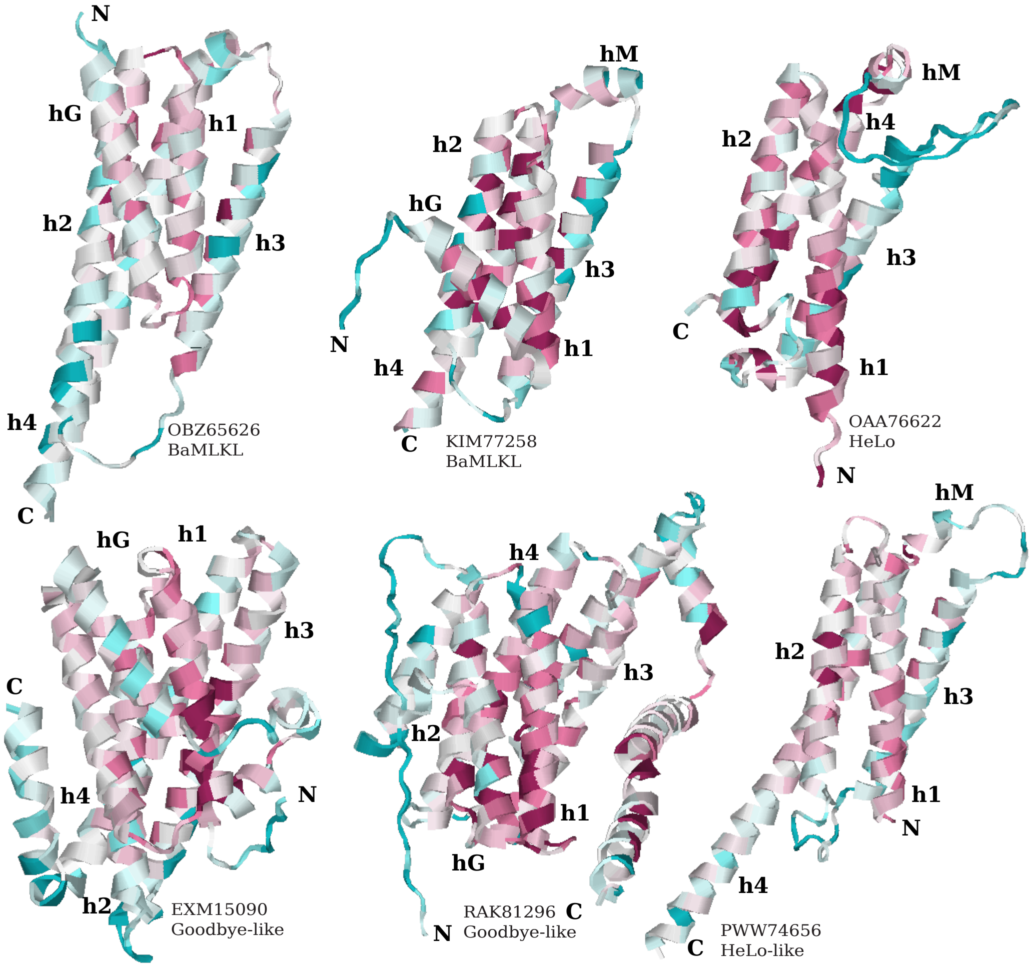

Figure C: **Alignment conservation in MLKL-like domains**. Structural models of various MLKL-like domains predicted with AlphaFold2 (see Methods and Fig 2 in the main text). Alignment conservation was scored and visualized in the cyan–red scale (low to high conservation) according to ConSurf.

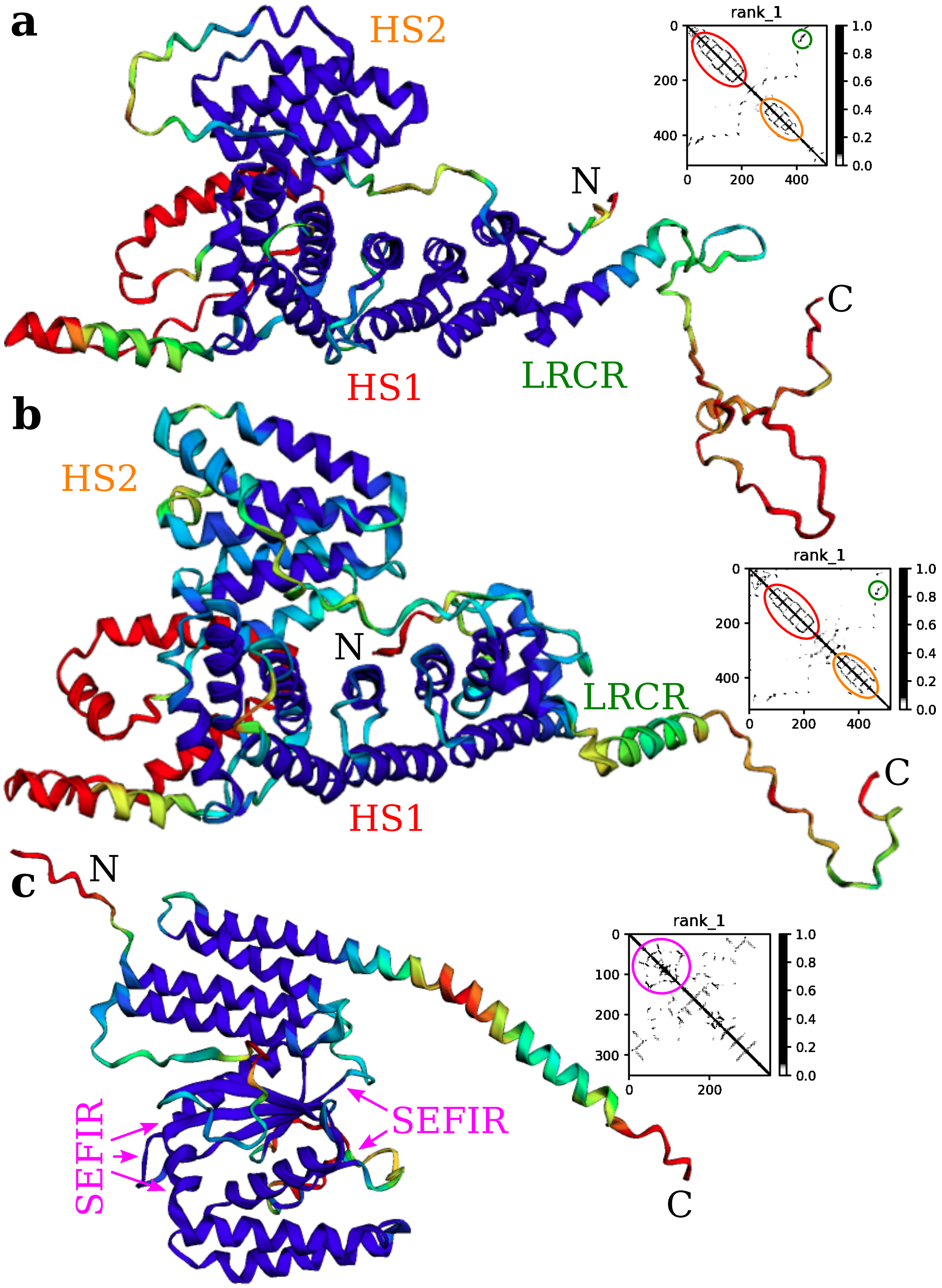

Figure D: **Structural models of three unannotated domains** predicted with AlphaFold2. (**a**) KEY84097, (**b**) KFH66451, (**c**) PQE30966. Rainbow colors indicate model quality in terms of lDDT (below or 50: red, 60: yellow, 70: green, 80: cyan, above 90: blue). Insets show contact probability maps. Regions of special interests are marked with colored ellipses on insets and annotated on structural models. Notations: HS1,2—helix stretch 1,2; LRCR—long-range contact region; SEFIR—region matching Pfam SEFIR domain (PF08357).

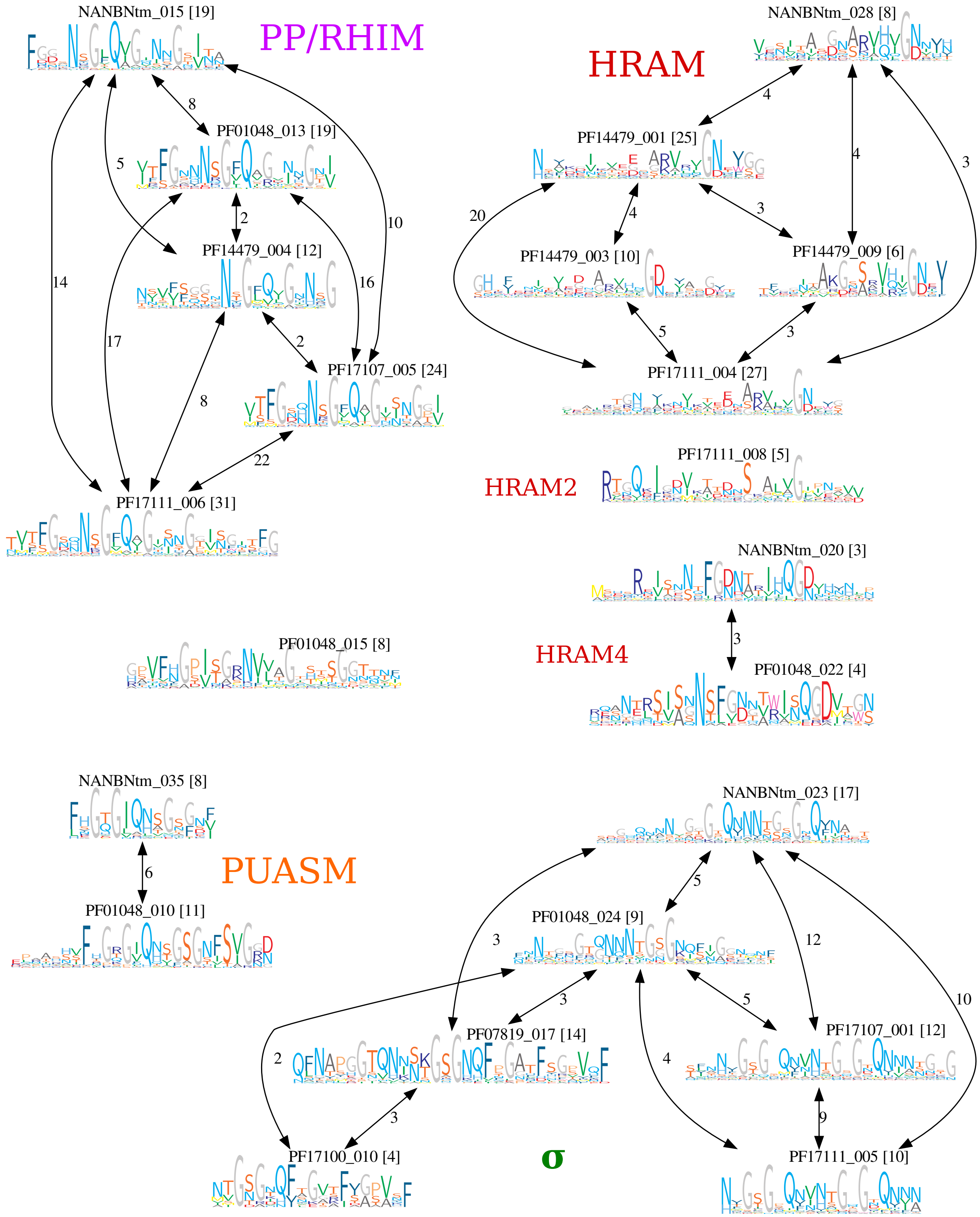

Figure E: **Clustering amyloid signaling motifs** identified through the effector domain-primed search. Motif id indicates the effector domain (PF...) or nucleotide-binding domain (NANB) next to which the seed motif was originally extracted (see Methods) and rank in the MEME extraction. The number in brackets represents the number of unique pairs of motifs instances for each profile, the number on the arrow joining the motif indicates the number of underlying pairs of sequences match, which are in common between linked profiles. Only edges with 2 or more matches are shown.

ESK80979.1_8_24	VNIHGG-HFNNIGRDQIN
ESK82249.1_16_32	VNINGG-QFNNVGRDQIN
ESK82448.1_8_25	VTIHDGEFQNLVYGDQVN
ESK83251.1_10_26	VHFSGG-QFSNIVNGPQI
ESK83762.1_16_32	VNISGG-QFNNVGRDQIN
ESK84058.1_9_25	FNISGG-QFTNVAGDQHY
ESK84408.1_181_198	GNISGNASFNTVGRDQHN
ESK85360.1_10_26	FQISGG-QFNVAGRDIHR
ESK85509.1_8_24	LNISGG-QFNNVGRDQIN
ESK86205.1_11_27	FDISGGT-FNVIHGVQNN
ESK86206.1_9_25	VKIRGGA-HNVVHGDQNS
ESK86209.1_10_26	FDISGGT-FNAVHGIQNN
ESK86709.1_9_26	VQIHGGEFRNVVYGNQDN
ESK86730.1_10_27	VDIHGGEFRNVVYGNQDN
ESK86868.1_10_26	VTVHGGA-RNMVHGDQHN
ESK86870.1_10_26	VTVHGGA-RNMVHGDQHN
ESK86939.1_8_24	VNINGG-HFNNVGRDQIN
ESK87464.1_8_24	VQVEGDV-INDIGRDQYI
ESK88197.1_8_23	FSITGG-DFV-VARDYHE
ESK90275.1_9_25	VHVQGDA-NNNVAGDQVN
ESK90280.1_8_24	VHVARDA-KNIVYGDQIN
ESK90283.1_8_24	TAIHGGA-HSTVGRDQNV
ESK90535.1_8_24	FSVHGG-HFNVAGRDINN
ESK91378.1_8_24	AIVHGDA-HNTVGRDQNV
ESK91749.1_9_25	VSIHGGA-YNLVHGEQMN
ESK93417.1_8_24	ATIWGDA-HNNVGRDQSH
ESK94148.1_10_26	VHVSGG-QLIAIGRDVNH
ESK94684.1_208_225	SSLSGNIKFNNVVGDQSN

logo

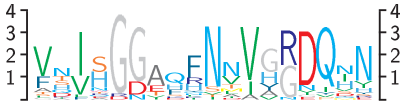

Figure F: **Alignment of candidate amyloid signaling motifs in *Moniliophthora roreri*** (strain MCA 2997). NLR-side N-terminal motifs and MLKL-like-side C-terminal motifs were aligned with Clustal Omega in *auto* mode.

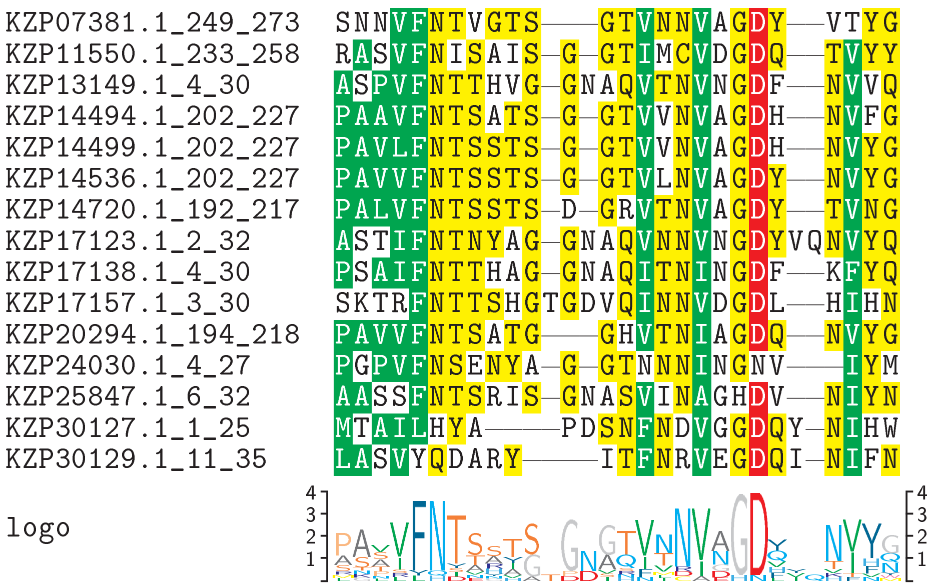

Figure G: **Alignment of candidate amyloid signaling motifs in *Fibularhizoctonia* sp. CBS 109695.** Eight NLR-side N-terminal motifs, five intra-protein motifs from MLKL-like–NLR proteins, and two MLKL-like-side C-terminal motifs (KZP11550, KZP14720) were aligned with Mafft in *linsi* mode.

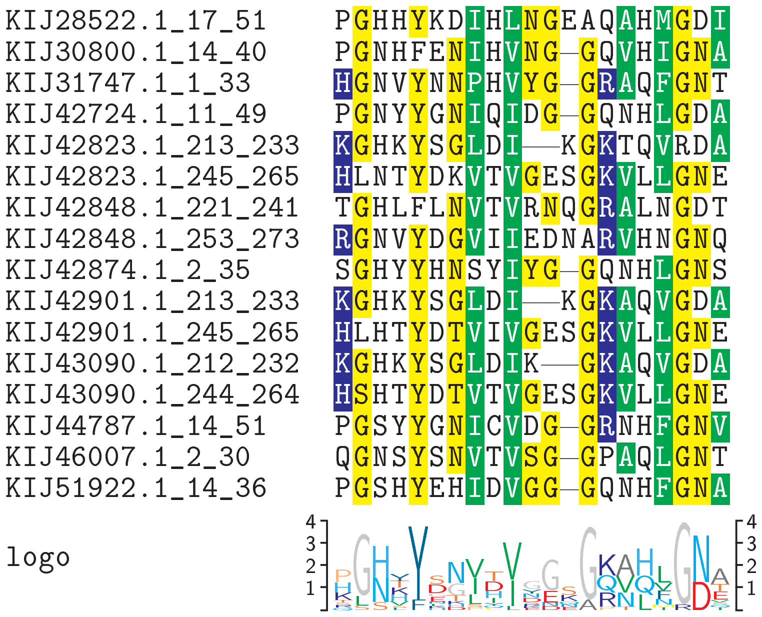

Figure H: **Alignment of candidate amyloid signaling motifs in *t*** (strain SS14). Eight NLR-side N-terminal motifs and four HeLo-side double C-terminal HRAMs were aligned with Mafft in *linsi* mode and refined manually.

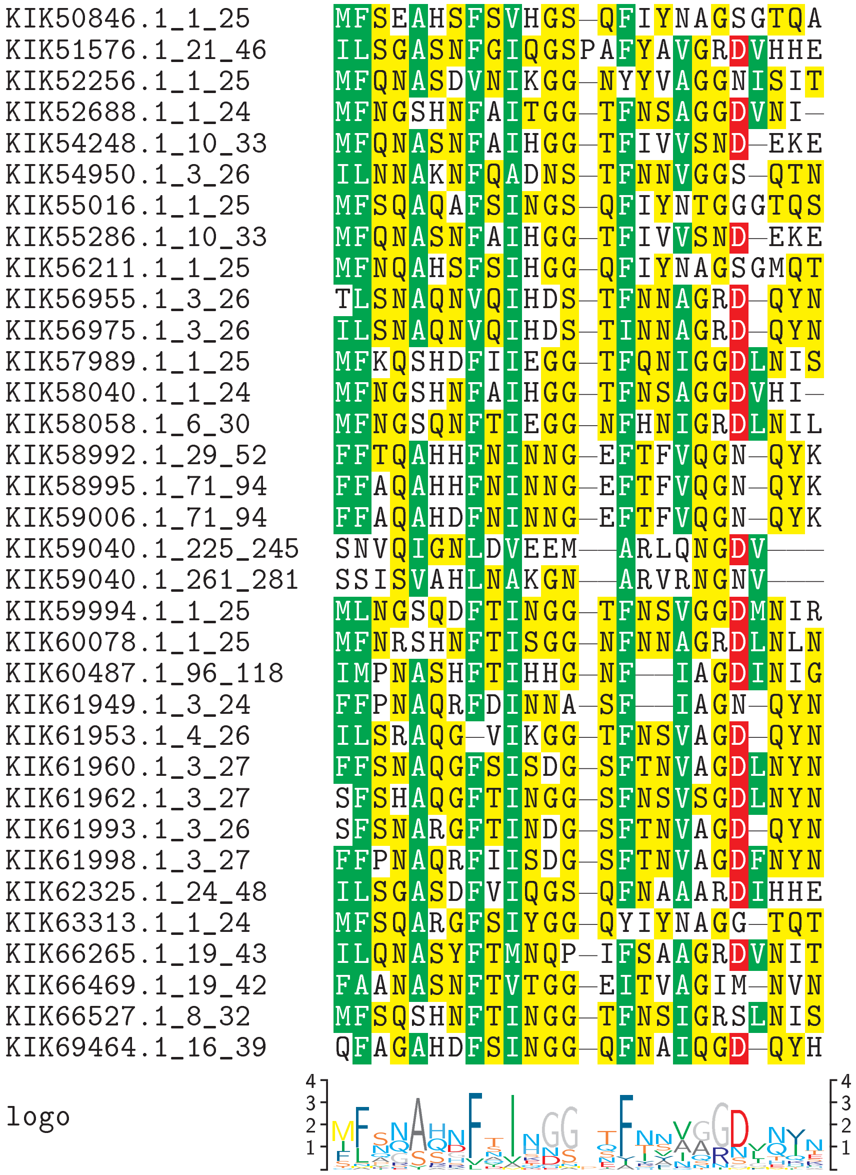

Figure I: **Alignment of candidate amyloid signaling motifs in *Gymnopus luxurians*** (strain FD-317 M1). NLR-side N-terminal motifs of NLR05/08/22/44 family were aligned with Mafft in *linsi* mode and trimmed manually. A HeLo-side double C-terminal HRAM was aligned to them manually.

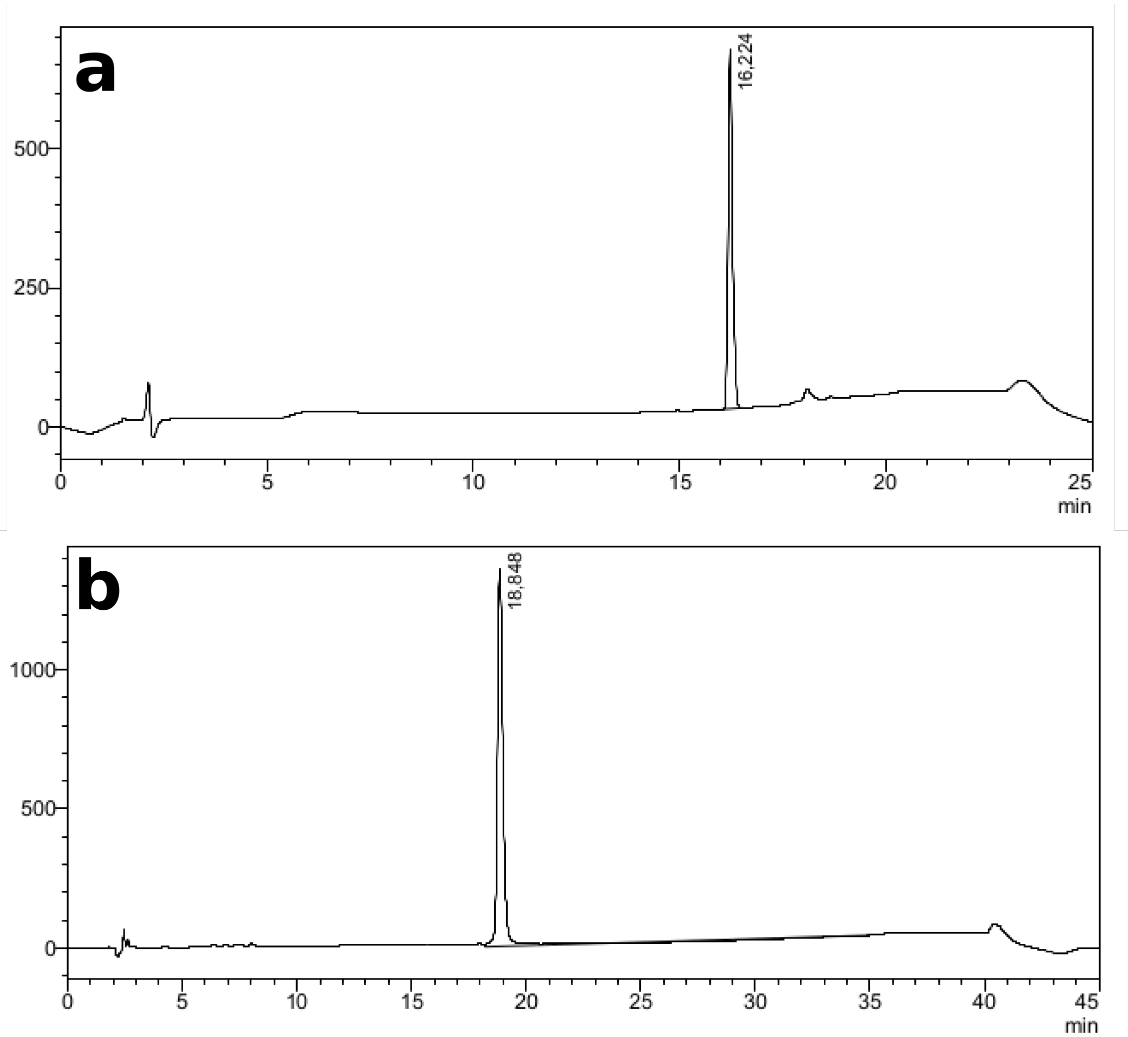

Figure J: **Analytical HPLC chromatograms of synthesized PUASM peptides.** **a**) EQB50682.1_332_355, **b**) EQB50683.1_9_31. Program (eluent A: 0.05% TFA in H2O, eluent B: 0.05% TFA in acetonitrile, flow 0.5 mL/min): A: t=0 min, 90% A; t=25 min for EQB50682.1_332_355 or t=45 min for EQB50683.1_9_31 10% A. See also Table C above and Experimental methods in the main text.

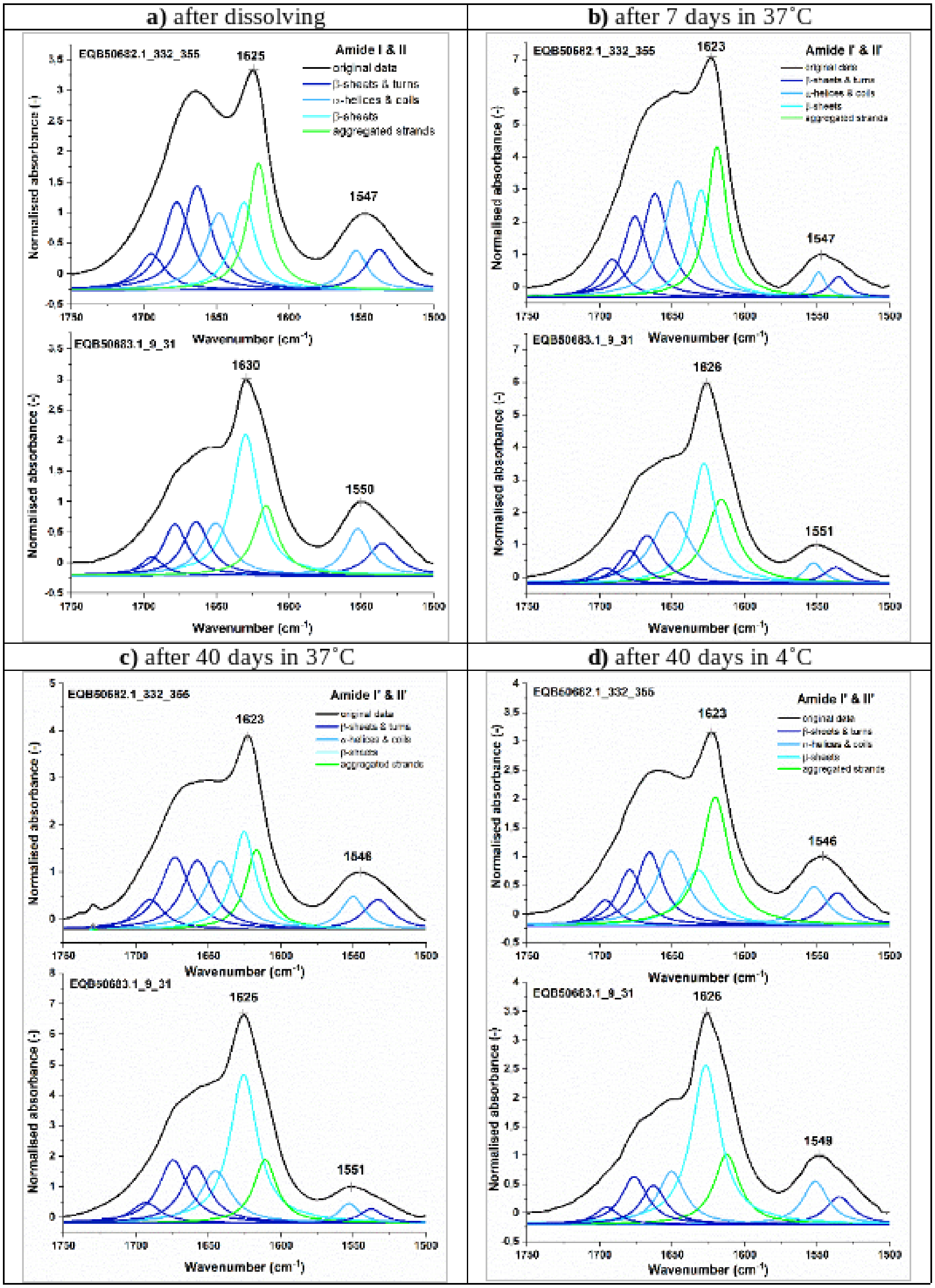

Figure K: **Normalized ATR-FTIR spectra of air-dried peptide films** of EQB50682.1_332_355 and EQB50683.1_9_31 with sub-bands obtained from the curve fitting procedure in the amide bands region (1750–1500 cm^{-1}) registered at a temperature of 20°: directly after dissolving (**a**), after 7 days (**b**) and 40 days of incubation process at 37°C (98.6°F) (**c**), and after 40 days of incubation at 4°C (39.2°F) (**d**).

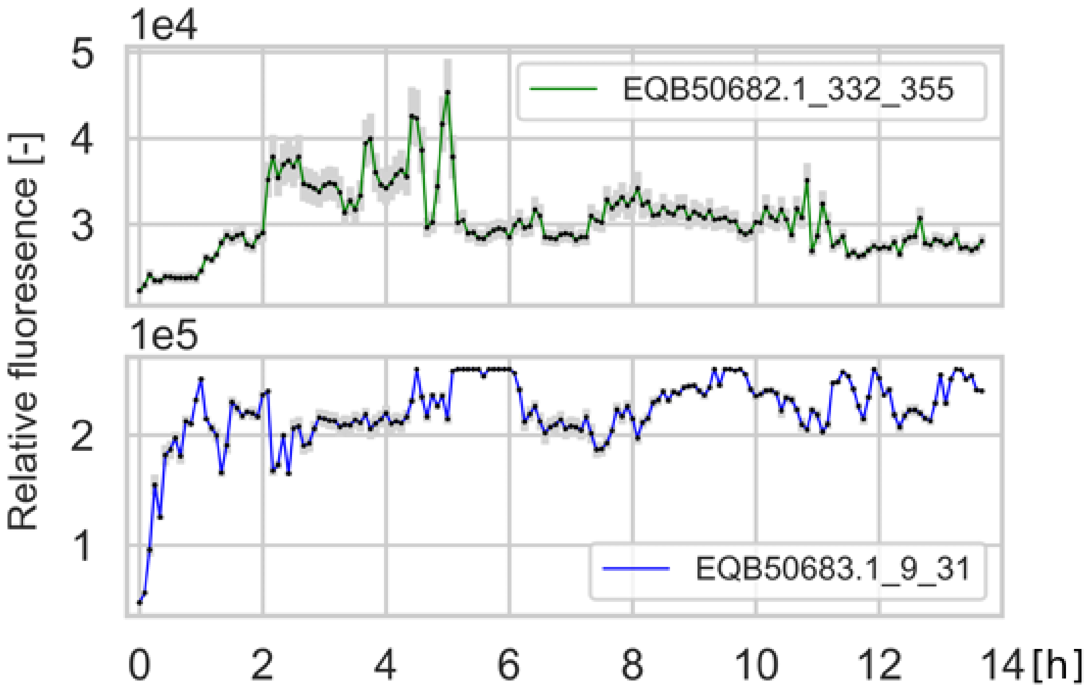

Figure L: **Aggregation kinetics** of peptides EQB50682.1_332_355 and EQB50683.1_9_31. See Results and Methods for details.

References

1. Xiao H, Kalman M, Ikehara K, Zemel S, Glaser G, Cashel M. Residual guanosine 3',5'-bispyrophosphate synthetic activity of relA null mutants can be eliminated by spoT null mutations. The Journal of Biological Chemistry. 1991;266(9):5980–5990.

2. Hanks SK, Quinn AM. Protein kinase catalytic domain sequence database: Identification of conserved features of primary structure and classification of family members. In: Protein Phosphorylation Part A: Protein Kinases: Assays, Purification, Antibodies, Functional Analysis, Cloning, and Expression. vol. 200 of Methods in Enzymology. Academic Press; 1991. p. 38–62.

3. Aravind L, Koonin EV. Classification of the caspase–hemoglobinase fold: Detection of new families and implications for the origin of the eukaryotic separins. Proteins: Structure, Function, and Bioinformatics. 2002;46(4):355–367.

4. Bonnert TP, Garka KE, Parnet P, Sonoda G, Testa JR, Sims JE. The cloning and characterization of human MyD88: a member of an IL-1 receptor related family 1. FEBS Letters. 1997;402(1):81–84.

5. Armant MA, Fenton MJ. Toll-like receptors: a family of pattern-recognition receptors in mammals. Genome biology. 2002;3(8):REVIEWS3011.

6. Ponting CP, Parker PJ. Extending the C2 domain family: C2s in PKCs delta, epsilon, eta, theta, phospholipases, GAPs, and perforin. Protein Science. 1996;5(1):162–166.

7. Daskalov A, Paoletti M, Ness F, Saupe SJ. Genomic Clustering and Homology between HET-S and the NWD2 STAND Protein in Various Fungal Genomes. PLoS ONE. 2012;7(4):e34854.

8. Dyrka W, Lamacchia M, Durrens P, Kobe B, Daskalov A, Paoletti M, et al. Diversity and Variability of NOD-Like Receptors in Fungi. Genome Biology and Evolution. 2014;6:3137–3158.

9. Ollis DL, Cheah E, Cygler M, Dijkstra B, Frolow F, Franken SM, et al. The alpha/beta hydrolase fold. Protein Engineering, Design and Selection. 1992;5(3):197–211.

10. Daskalov A, Dyrka W, Saupe SJ. 2. In: Benz JP, Schipper K, editors. NLR Function in Fungi as Revealed by the Study of Self/Non-self Recognition Systems. Cham: Springer International Publishing; 2020. p. 123–141.

11. Remmert M, Biegert A, Hauser A, Soeding J. HHblits: lightning-fast iterative protein sequence searching by HMM-HMM alignment. Nature Methods. 2012;9(2):173–175.

12. Jumper J, Evans R, Pritzel A, Green T, Figurnov M, Ronneberger O, et al. Highly accurate protein structure prediction with AlphaFold. Nature. 2021;596(7873):583–589.

13. Mirdita M, Schütze K, Moriwaki Y, Heo L, Ovchinnikov S, Steinegger M. ColabFold: making protein folding accessible to all. Nature methods. 2022;19(6):679—682.

14. Uehling J, Deveau A, Paoletti M. Do fungi have an innate immune response? An NLR-based comparison to plant and animal immune systems. PLoS Pathogens. 2017;13(10):e1006578.

15. Ghabrial SA, Nibert ML. Victorivirus, a new genus of fungal viruses in the family Totiviridae. Archives of Virology. 2009;154:373–379.

16. Jethon AM. Potential virual symbiont in leaf cutter ant system. The Pennsylvania State University; 2018.

17. Jo Y, Choi H, Chu H, Cho WK. Identification of viruses from fungal transcriptomes. bioRxiv. 2020;doi:10.1101/2020.02.26.966903.

18. Polekhina G, Gupta A, van Denderen BJW, Feil SC, Kemp BE, Stapleton D, et al. Structural basis for glycogen recognition by AMP-activated protein kinase. Structure (London, England : 1993). 2005;13(10):1453–1462.

19. Novatchkova M, Leibbrandt A, Werzowa J, Neubüser A, Eisenhaber F. The STIR-domain superfamily in signal transduction, development and immunity. Trends in biochemical sciences. 2003;28(5):226–229.

20. Wu B, Gong J, Liu L, Li T, Wei T, Bai Z. Evolution of prokaryotic homologues of the eukaryotic SEFIR protein domain. Gene. 2012;492(1):160–166.

21. Jacob F, Vernaldi S, Maekawa T. Evolution and Conservation of Plant NLR Functions. Frontiers in Immunology. 2013;4:297.

22. Dyrka W, Coustou V, Daskalov A, Lends A, Bardin T, Berbon M, et al. Identification of NLR-associated amyloid signaling motifs in bacterial genomes. Journal of Molecular Biology. 2020;432(23):6005–6027.

23. Guo Y, Narisawa K. Fungus-Bacterium Symbionts Promote Plant Health and Performance. Microbes and Environments. 2018;33(3):239–241.

24. Finn RD, Coggill P, Eberhardt RY, Eddy SR, Mistry J, Mitchell AL, et al. The Pfam protein families database: towards a more sustainable future. Nucleic Acids Research. 2016;.
