# Supplementary figures and images for: Exploring a diverse world of effector domains and amyloid signaling motifs in fungal NLR proteins
Source: PLoS Comput Biol. 2022 Dec 21;18(12):e1010787. doi: 10.1371/journal.pcbi.1010787 (PMC9815663; doi:10.1371/journal.pcbi.1010787)

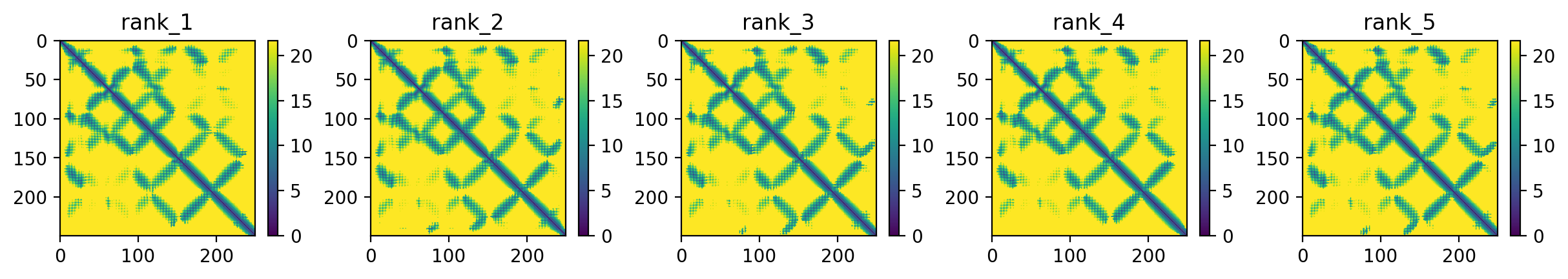

Supplement: S3 Data — Full AlphaFold2/ColabFold outputs. (GZ) [file pcbi.1010787.s010.tar.gz › EXM15090_1_250/predicted_distogram.png]

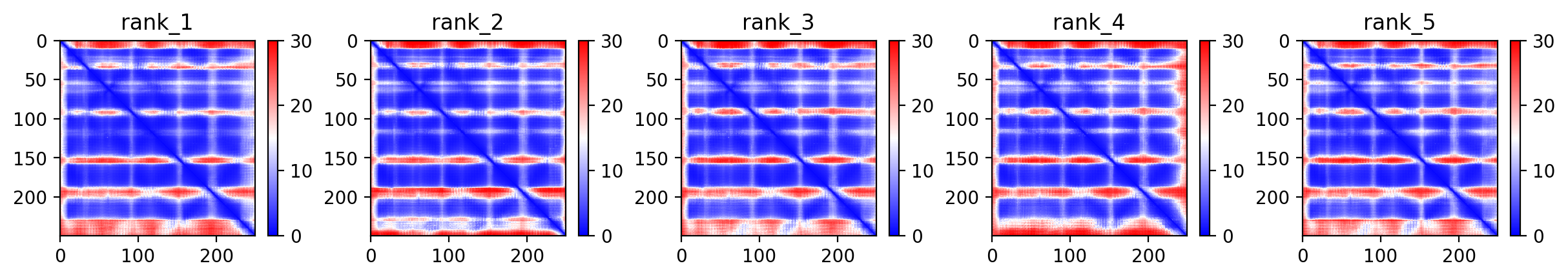

Supplement: S3 Data — Full AlphaFold2/ColabFold outputs. (GZ) [file pcbi.1010787.s010.tar.gz › EXM15090_1_250/predicted_alignment_error.png]

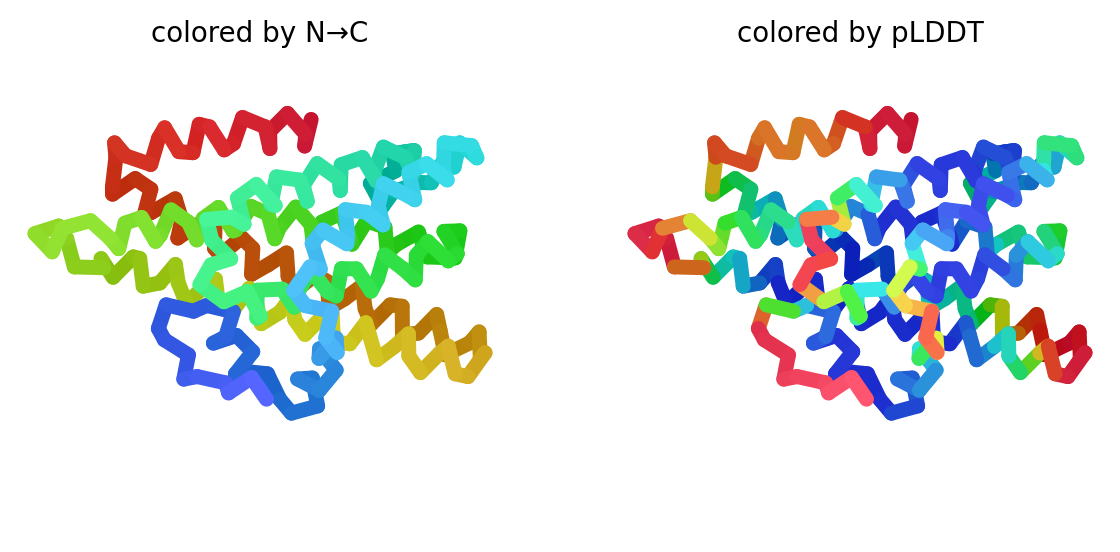

Supplement: S3 Data — Full AlphaFold2/ColabFold outputs. (GZ) [file pcbi.1010787.s010.tar.gz › EXM15090_1_250/rank_5_model_2_ptm_seed_0.png]

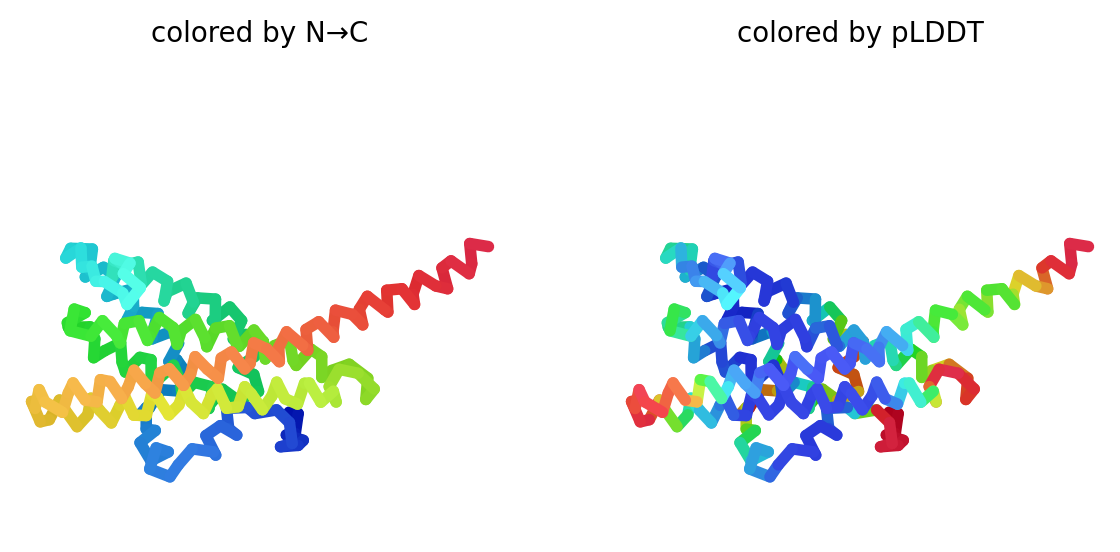

Supplement: S3 Data — Full AlphaFold2/ColabFold outputs. (GZ) [file pcbi.1010787.s010.tar.gz › EXM15090_1_250/rank_4_model_1_ptm_seed_0.png]

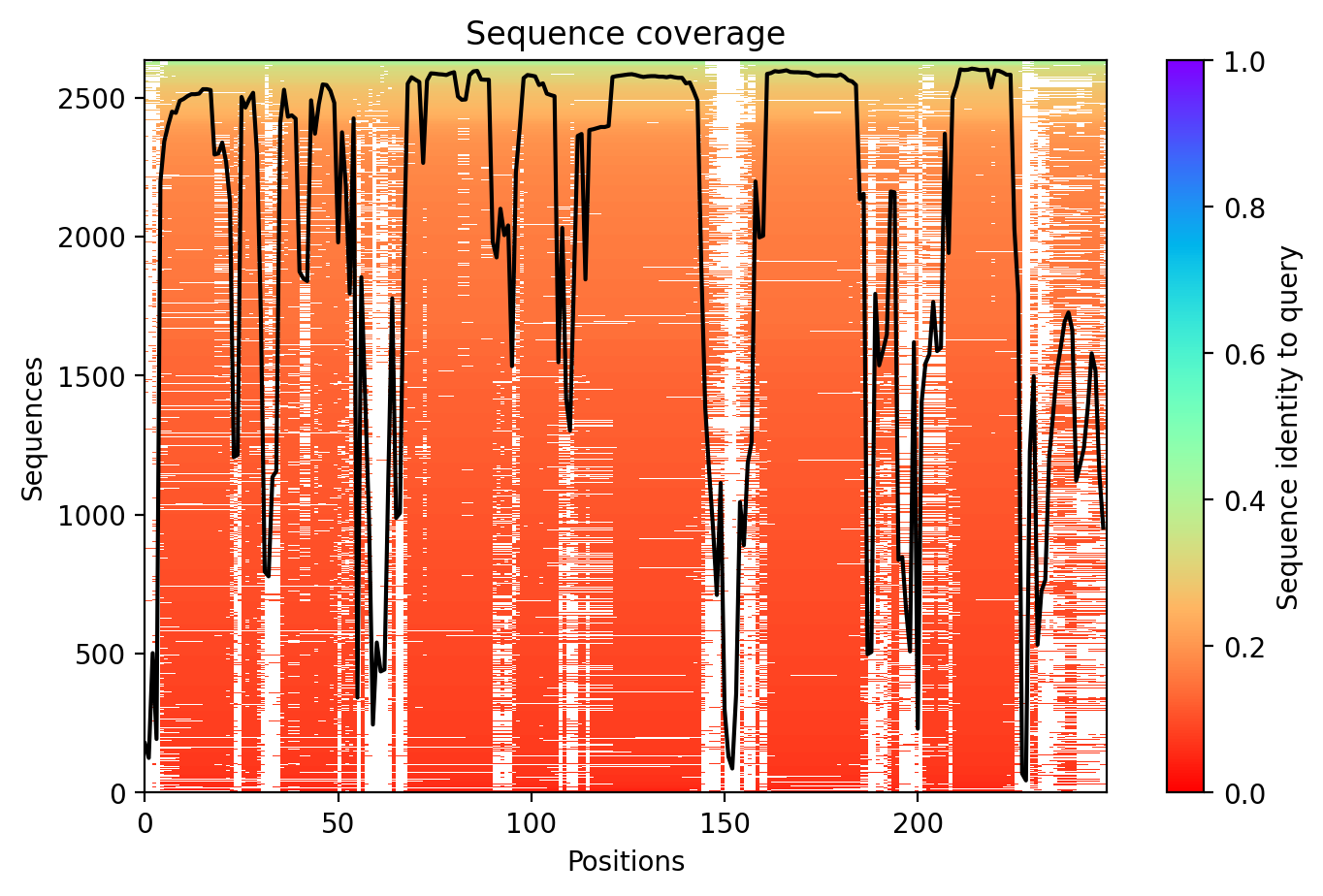

Supplement: S3 Data — Full AlphaFold2/ColabFold outputs. (GZ) [file pcbi.1010787.s010.tar.gz › EXM15090_1_250/msa_coverage.png]

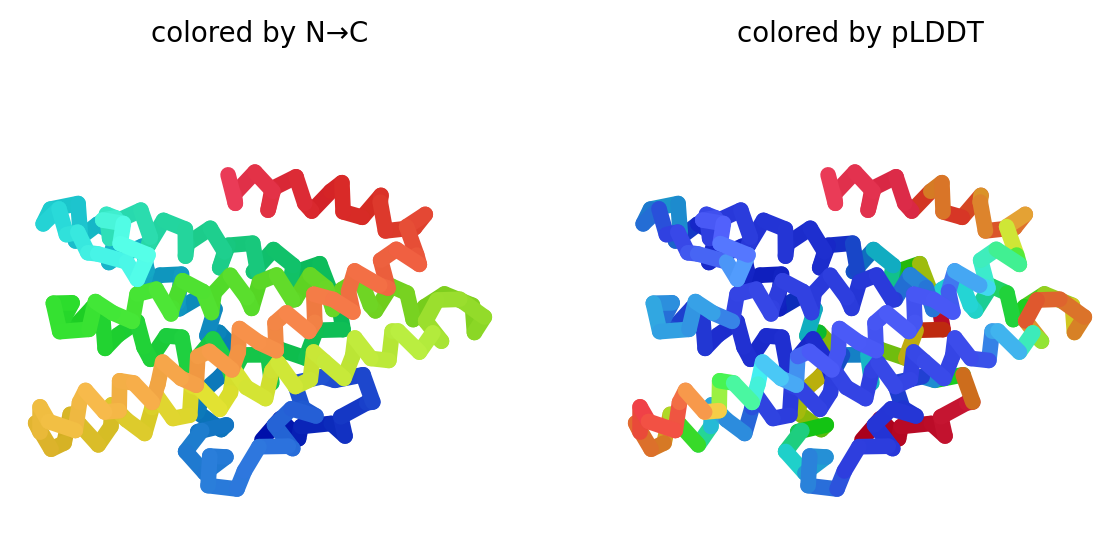

Supplement: S3 Data — Full AlphaFold2/ColabFold outputs. (GZ) [file pcbi.1010787.s010.tar.gz › EXM15090_1_250/rank_1_model_3_ptm_seed_0.png]

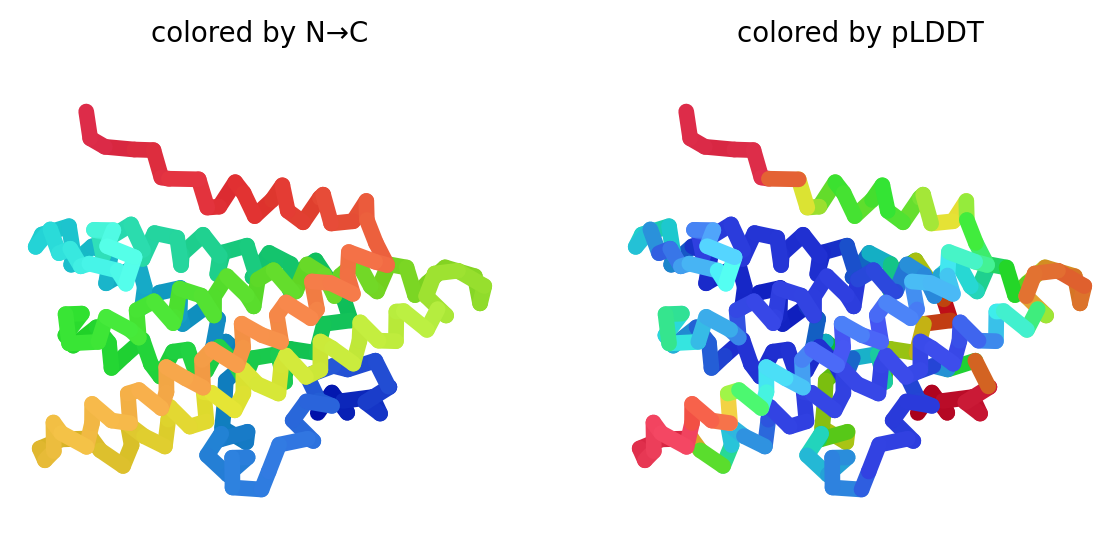

Supplement: S3 Data — Full AlphaFold2/ColabFold outputs. (GZ) [file pcbi.1010787.s010.tar.gz › EXM15090_1_250/rank_2_model_5_ptm_seed_0.png]

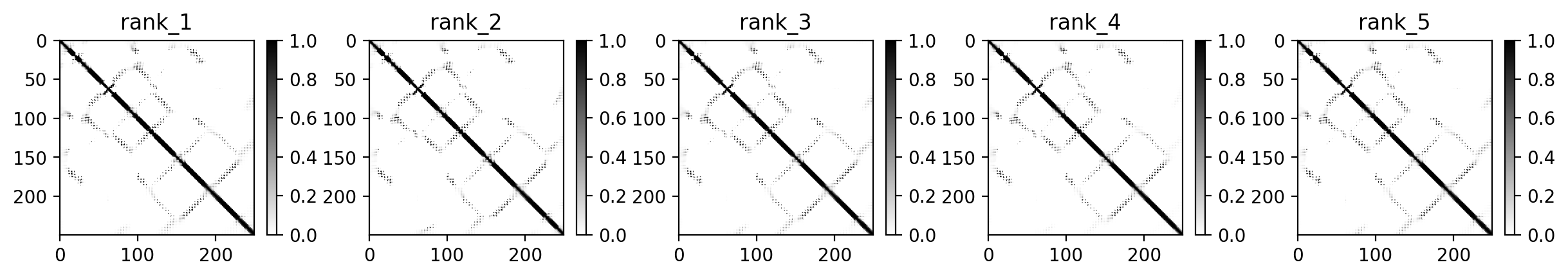

Supplement: S3 Data — Full AlphaFold2/ColabFold outputs. (GZ) [file pcbi.1010787.s010.tar.gz › EXM15090_1_250/predicted_contacts.png]

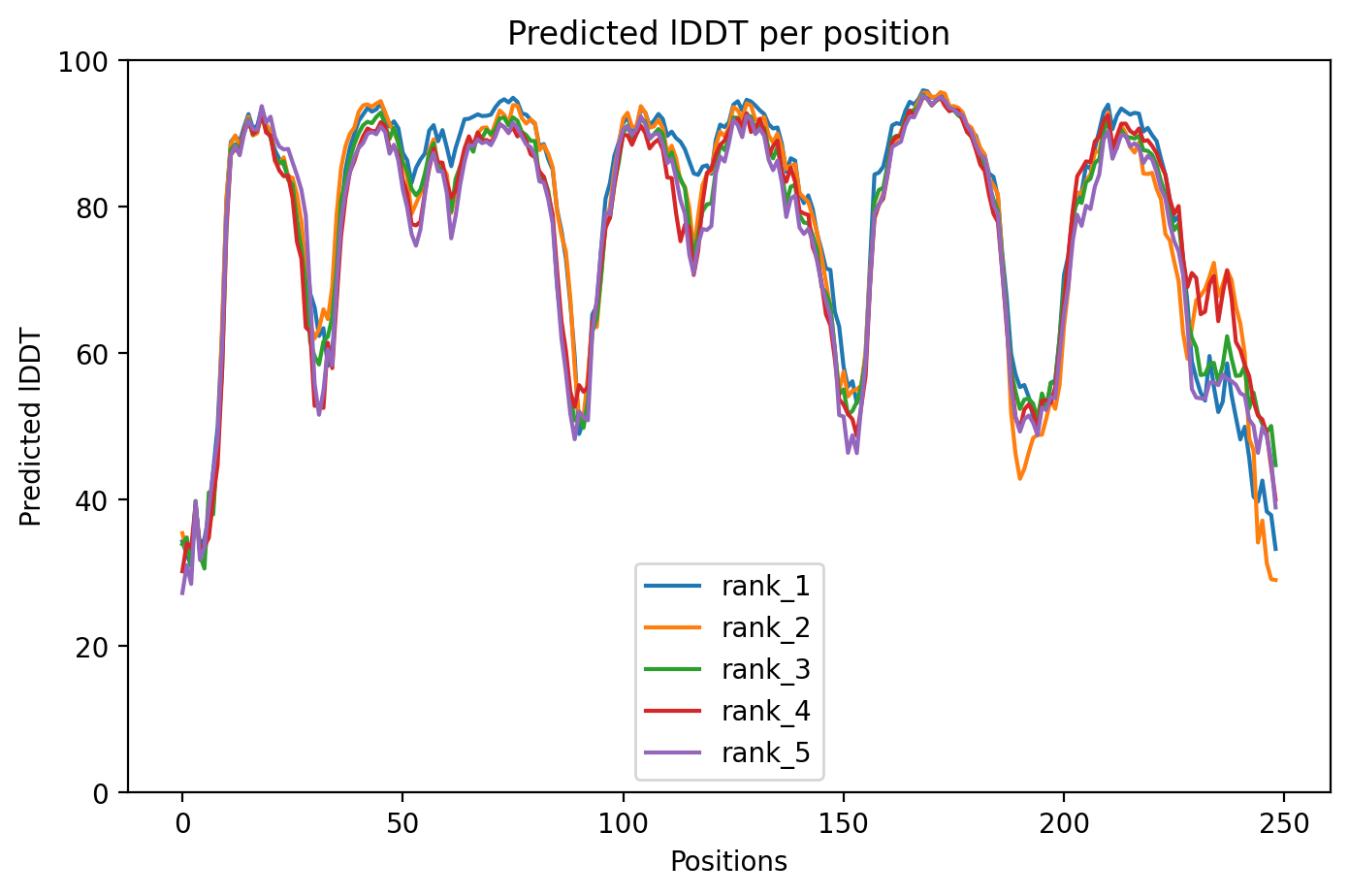

Supplement: S3 Data — Full AlphaFold2/ColabFold outputs. (GZ) [file pcbi.1010787.s010.tar.gz › EXM15090_1_250/predicted_LDDT.png]

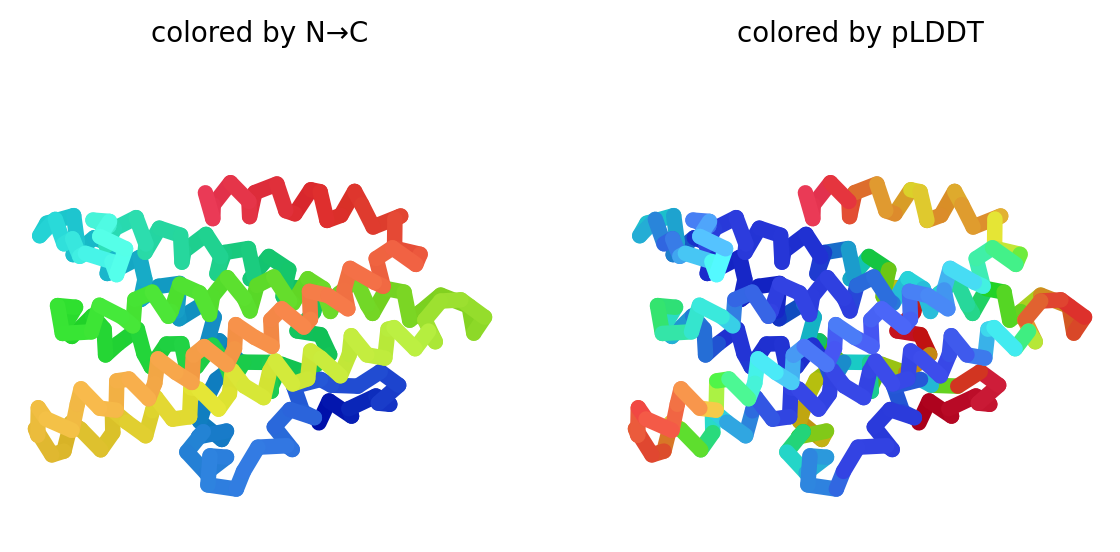

Supplement: S3 Data — Full AlphaFold2/ColabFold outputs. (GZ) [file pcbi.1010787.s010.tar.gz › EXM15090_1_250/rank_3_model_4_ptm_seed_0.png]

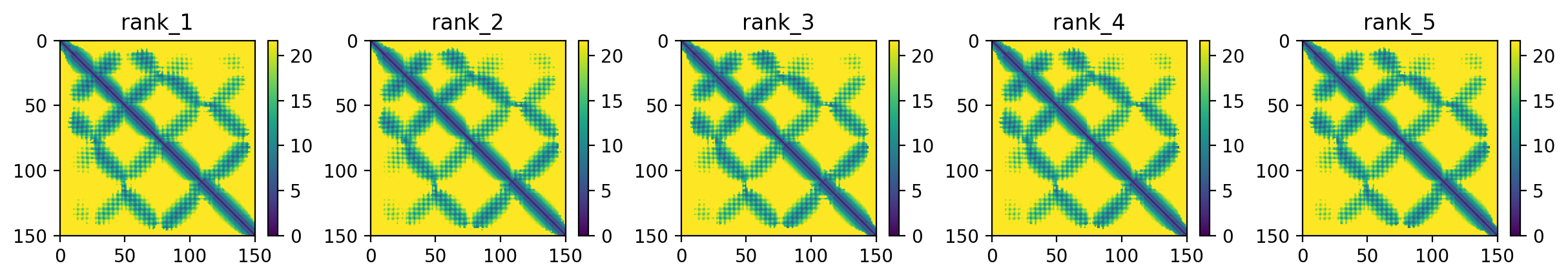

Supplement: S3 Data — Full AlphaFold2/ColabFold outputs. (GZ) [file pcbi.1010787.s010.tar.gz › KIM77258_1_150/predicted_distogram.png]

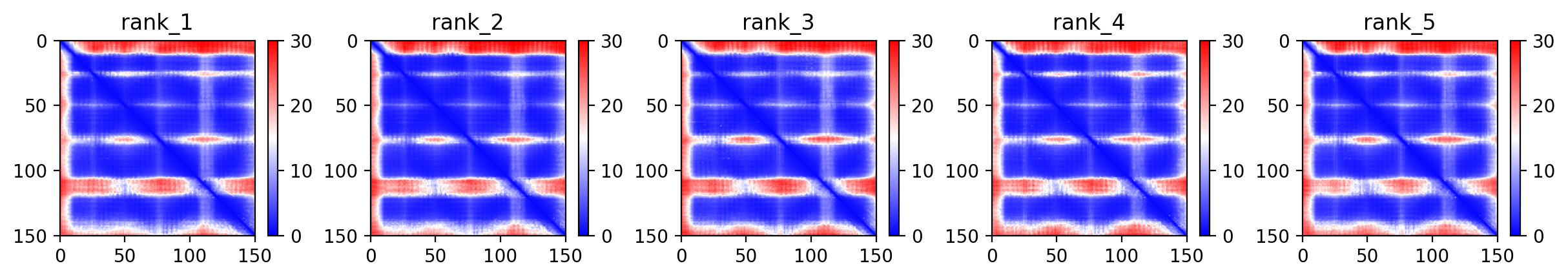

Supplement: S3 Data — Full AlphaFold2/ColabFold outputs. (GZ) [file pcbi.1010787.s010.tar.gz › KIM77258_1_150/predicted_alignment_error.png]

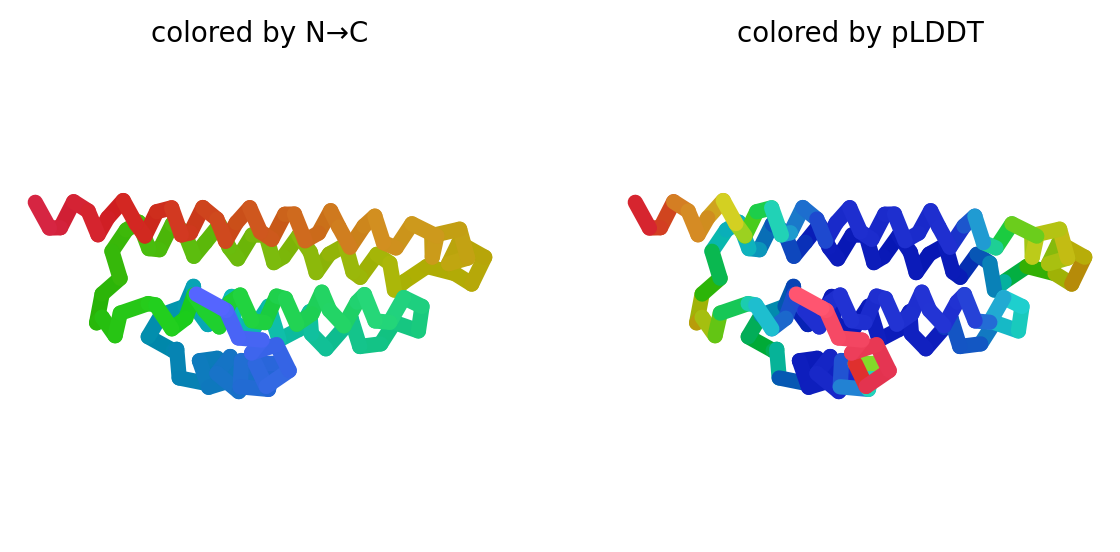

Supplement: S3 Data — Full AlphaFold2/ColabFold outputs. (GZ) [file pcbi.1010787.s010.tar.gz › KIM77258_1_150/rank_5_model_2_ptm_seed_0.png]

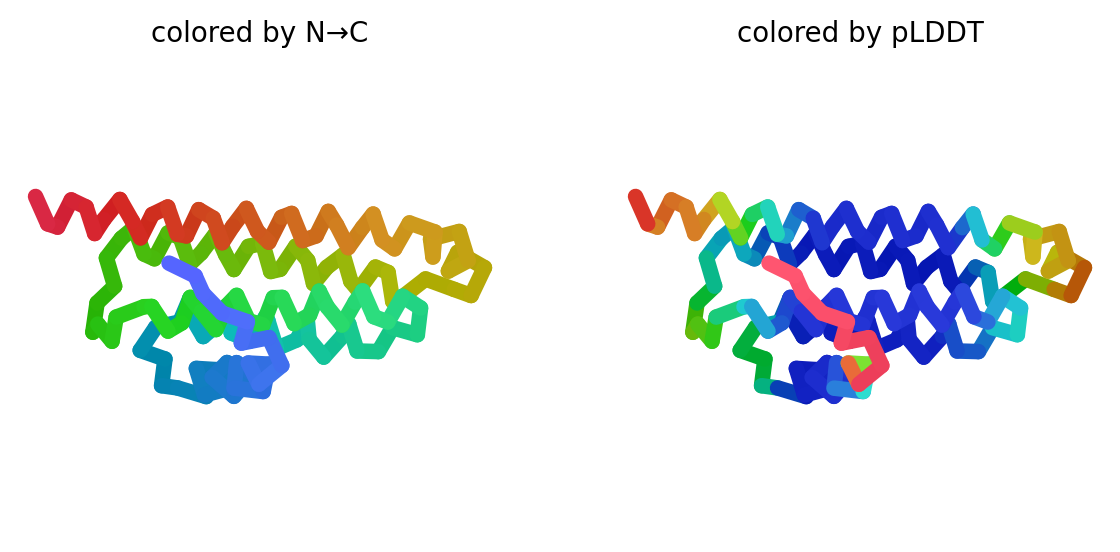

Supplement: S3 Data — Full AlphaFold2/ColabFold outputs. (GZ) [file pcbi.1010787.s010.tar.gz › KIM77258_1_150/rank_4_model_1_ptm_seed_0.png]

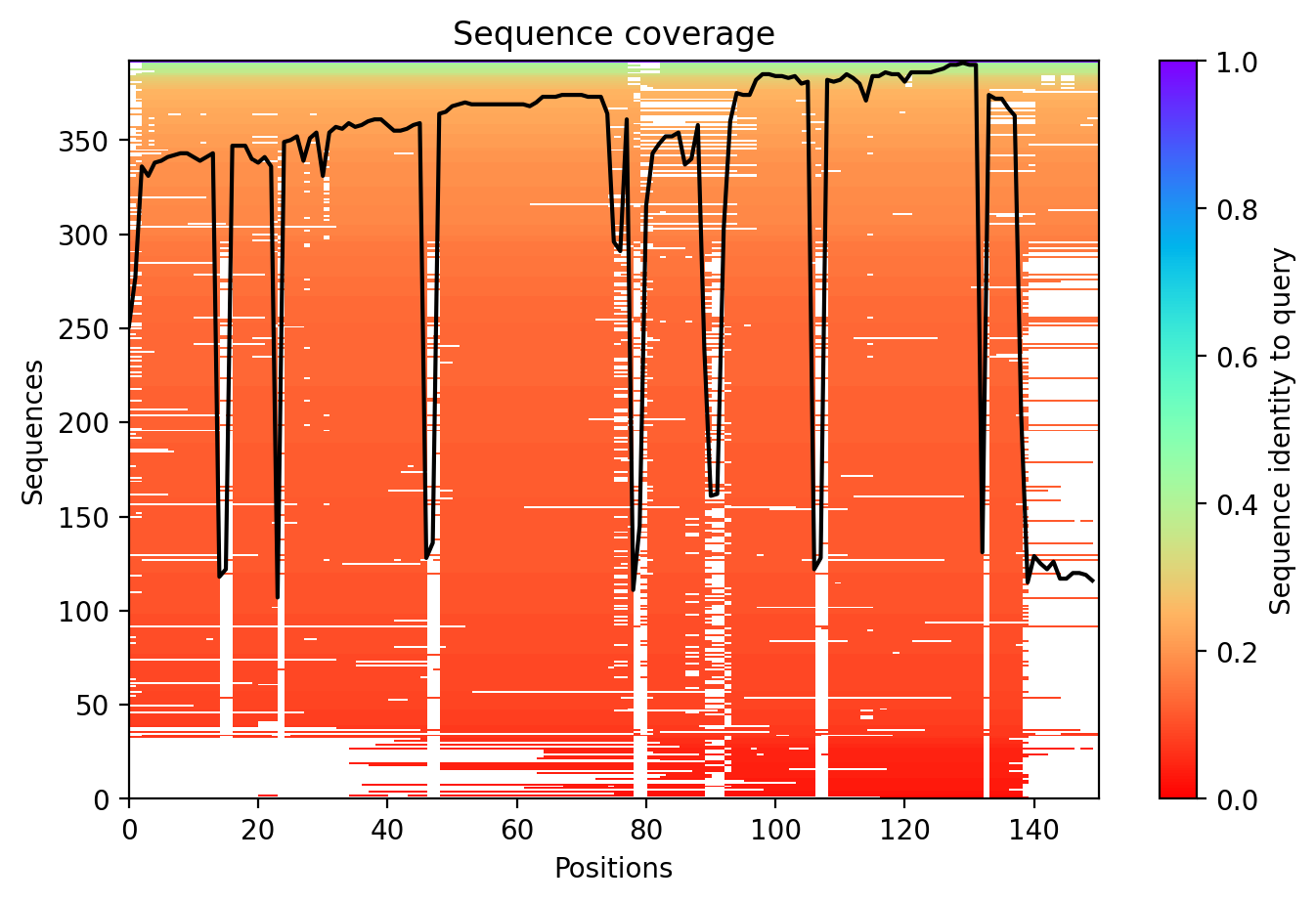

Supplement: S3 Data — Full AlphaFold2/ColabFold outputs. (GZ) [file pcbi.1010787.s010.tar.gz › KIM77258_1_150/msa_coverage.png]

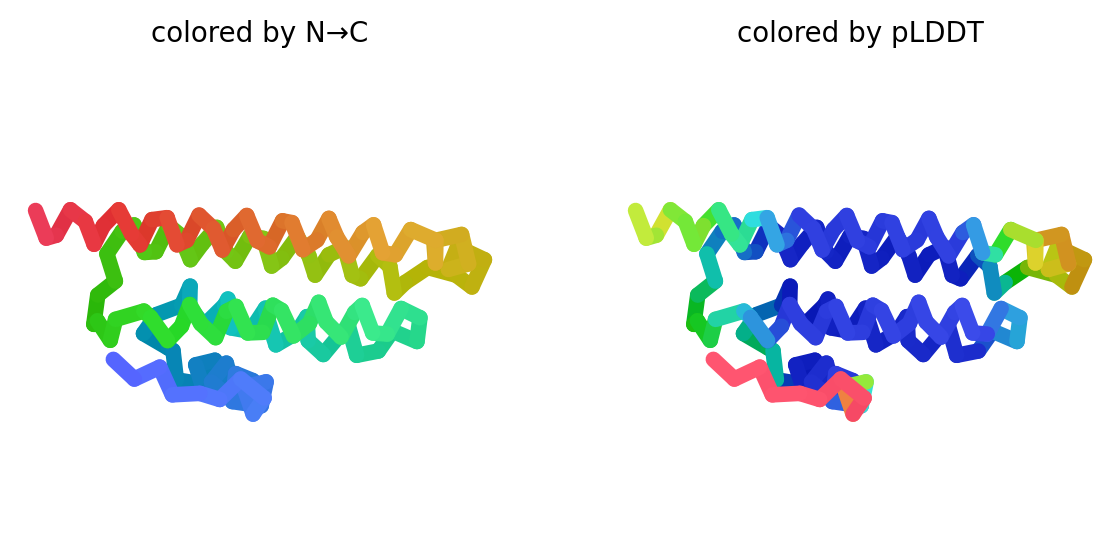

Supplement: S3 Data — Full AlphaFold2/ColabFold outputs. (GZ) [file pcbi.1010787.s010.tar.gz › KIM77258_1_150/rank_1_model_3_ptm_seed_0.png]

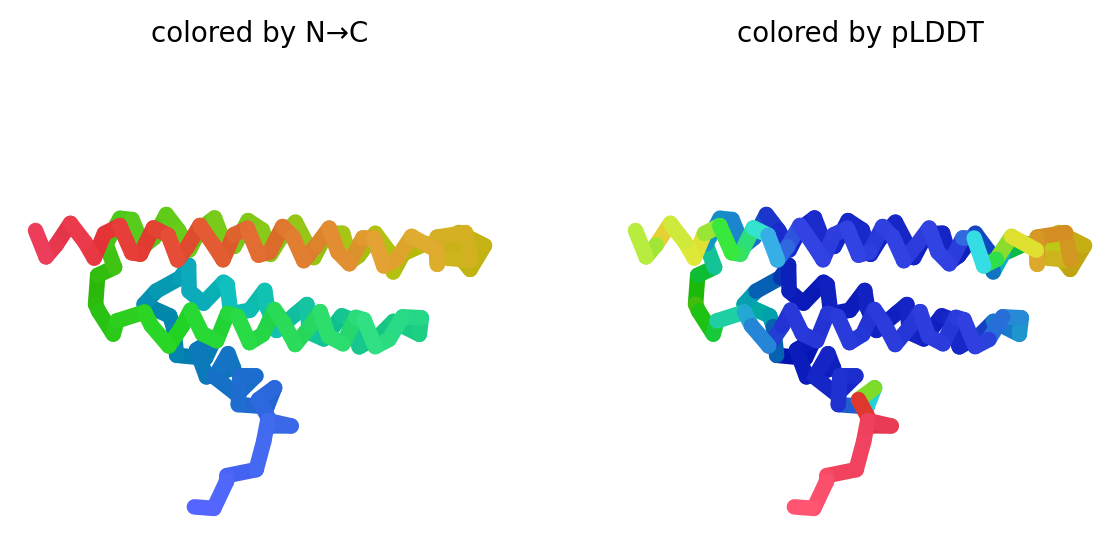

Supplement: S3 Data — Full AlphaFold2/ColabFold outputs. (GZ) [file pcbi.1010787.s010.tar.gz › KIM77258_1_150/rank_2_model_5_ptm_seed_0.png]

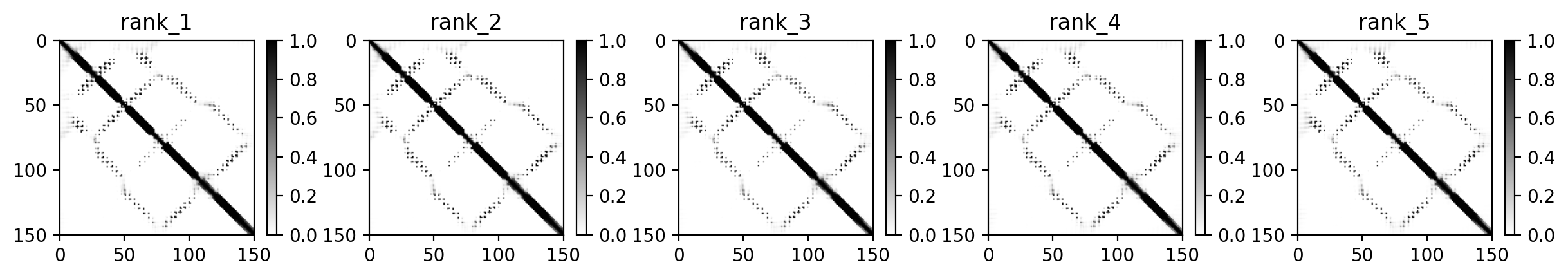

Supplement: S3 Data — Full AlphaFold2/ColabFold outputs. (GZ) [file pcbi.1010787.s010.tar.gz › KIM77258_1_150/predicted_contacts.png]

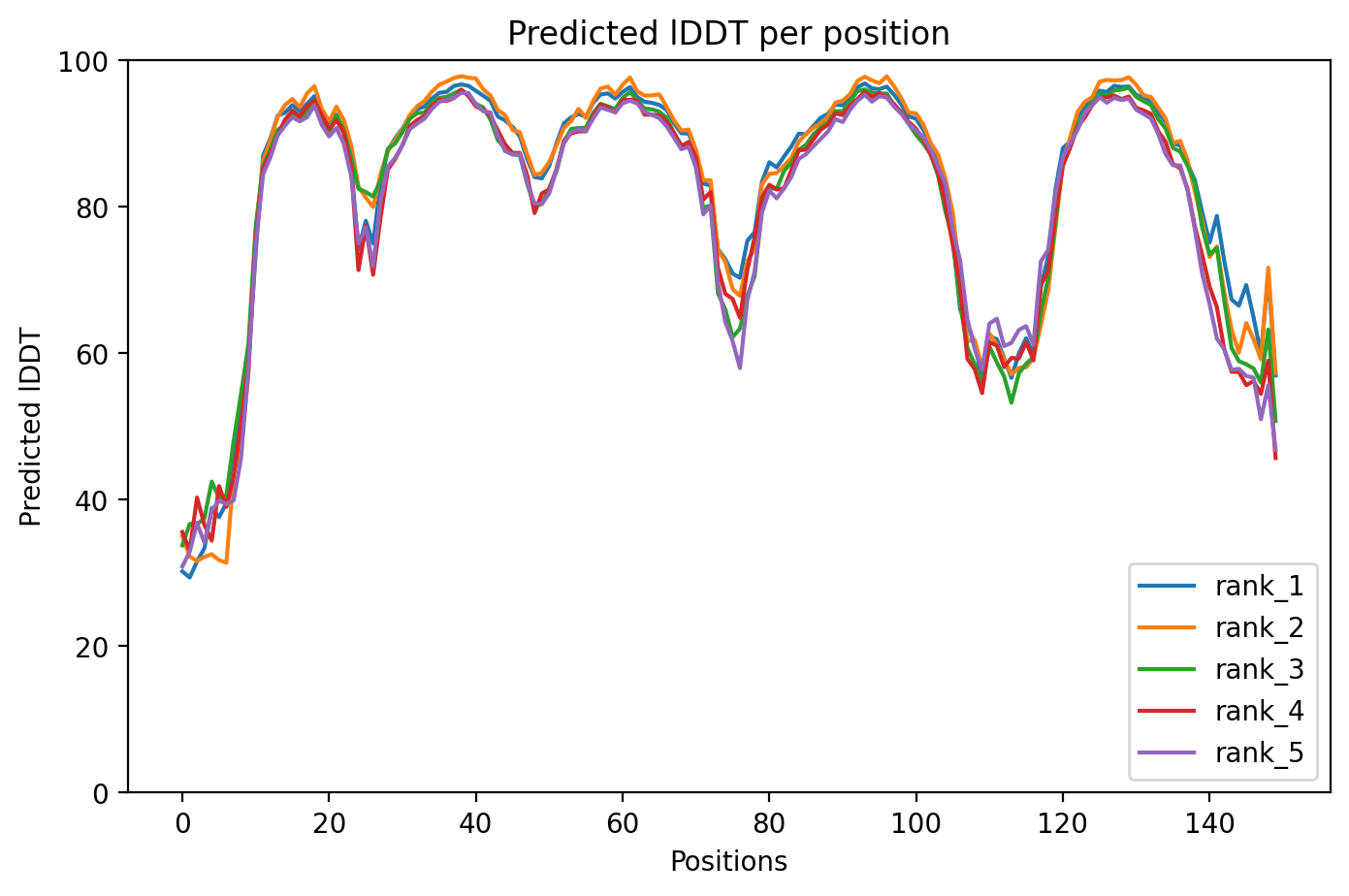

Supplement: S3 Data — Full AlphaFold2/ColabFold outputs. (GZ) [file pcbi.1010787.s010.tar.gz › KIM77258_1_150/predicted_LDDT.png]

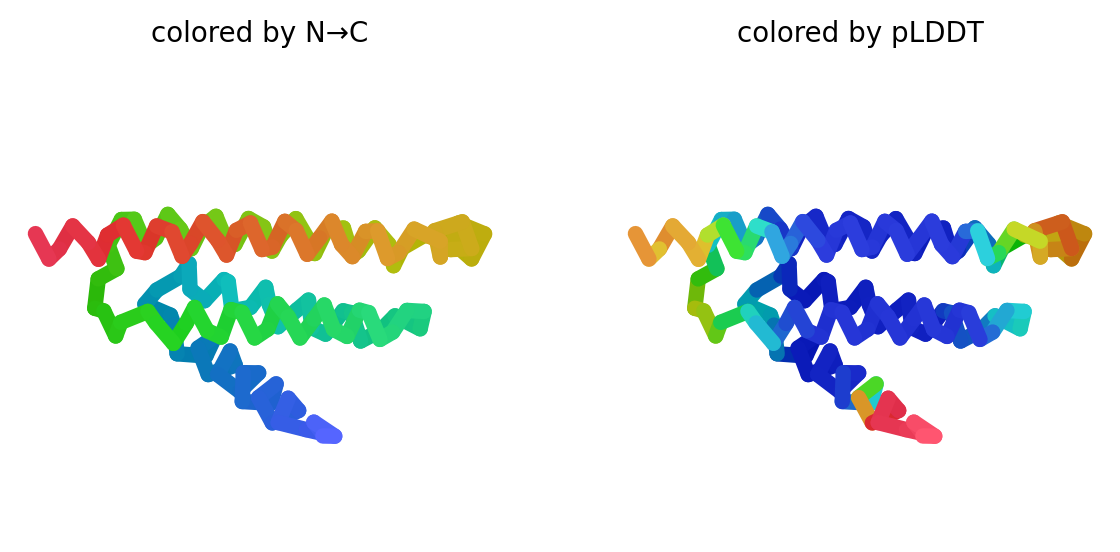

Supplement: S3 Data — Full AlphaFold2/ColabFold outputs. (GZ) [file pcbi.1010787.s010.tar.gz › KIM77258_1_150/rank_3_model_4_ptm_seed_0.png]

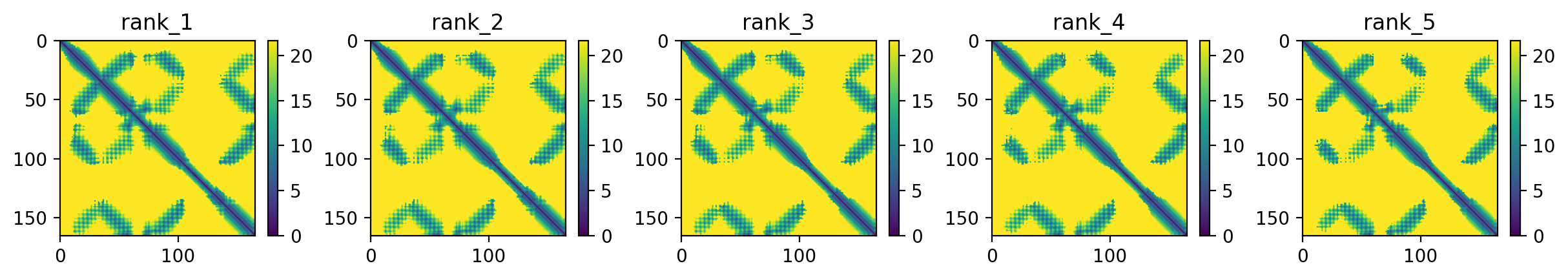

Supplement: S3 Data — Full AlphaFold2/ColabFold outputs. (GZ) [file pcbi.1010787.s010.tar.gz › OAA76622_1_165/predicted_distogram.png]

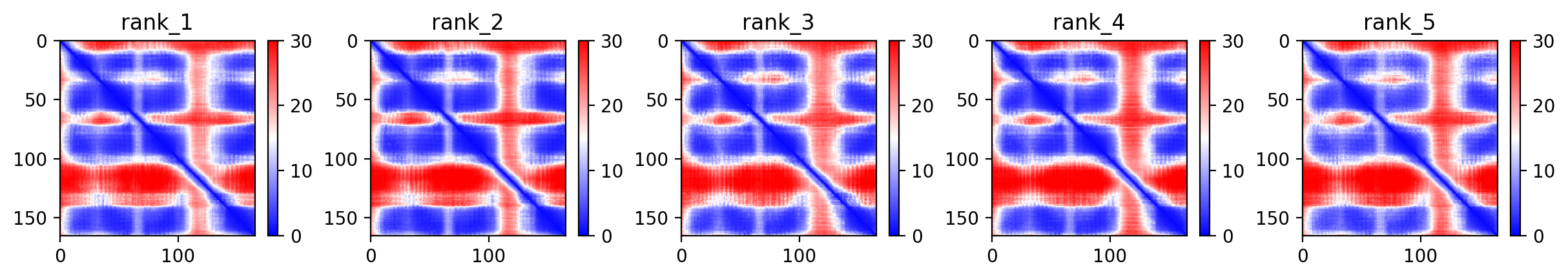

Supplement: S3 Data — Full AlphaFold2/ColabFold outputs. (GZ) [file pcbi.1010787.s010.tar.gz › OAA76622_1_165/predicted_alignment_error.png]

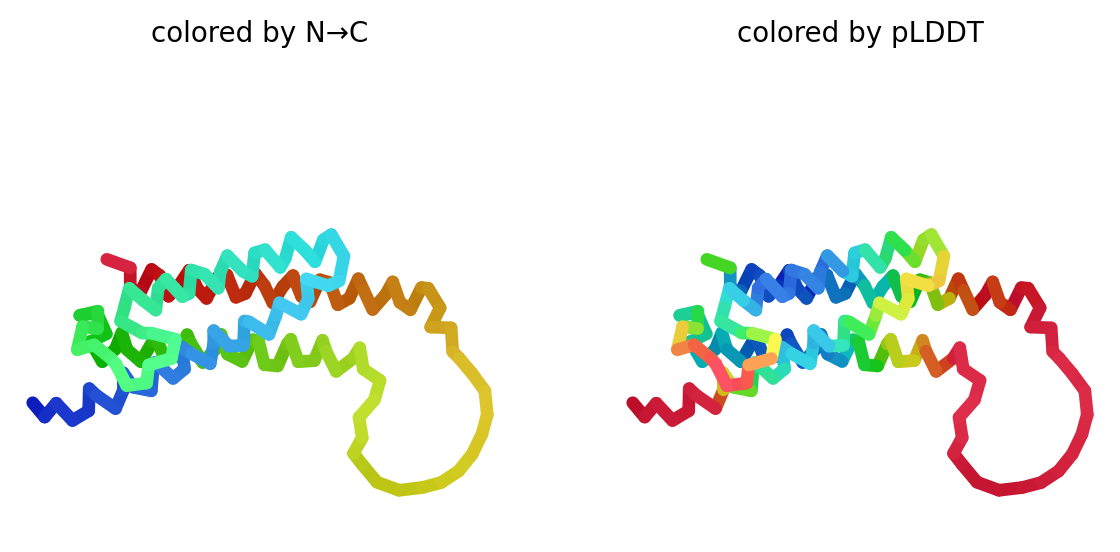

Supplement: S3 Data — Full AlphaFold2/ColabFold outputs. (GZ) [file pcbi.1010787.s010.tar.gz › OAA76622_1_165/rank_3_model_1_ptm_seed_0.png]

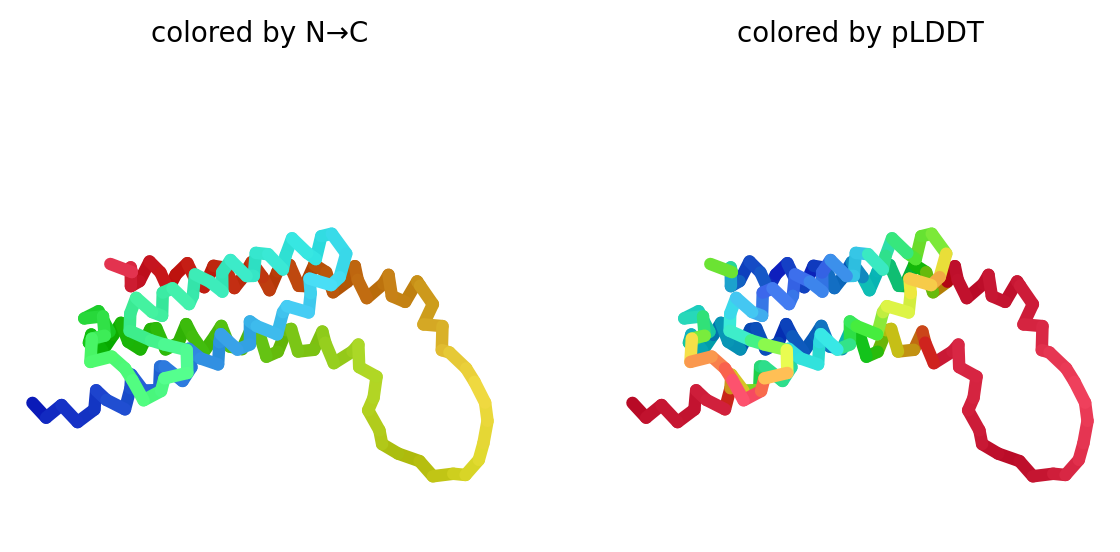

Supplement: S3 Data — Full AlphaFold2/ColabFold outputs. (GZ) [file pcbi.1010787.s010.tar.gz › OAA76622_1_165/rank_4_model_2_ptm_seed_0.png]

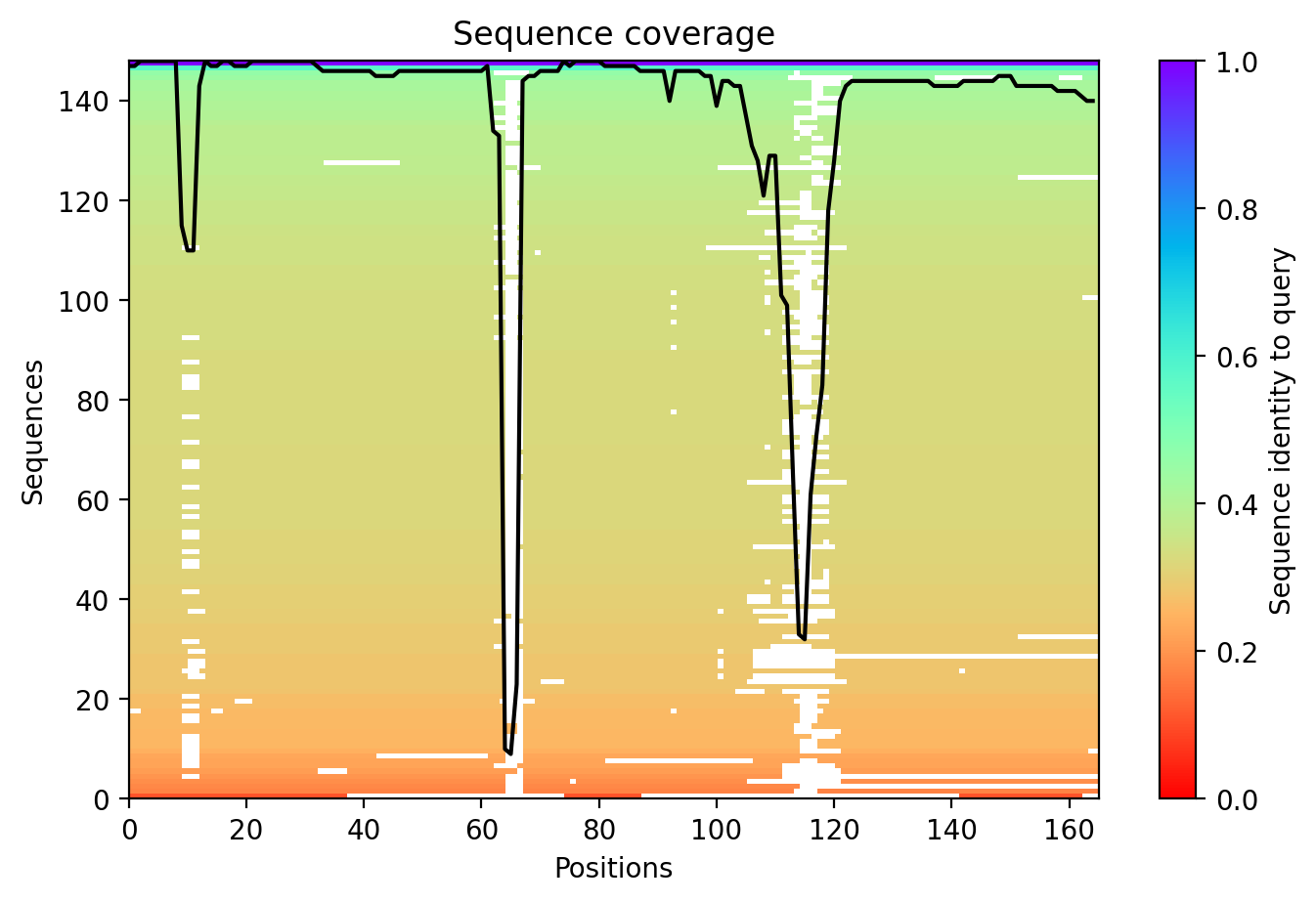

Supplement: S3 Data — Full AlphaFold2/ColabFold outputs. (GZ) [file pcbi.1010787.s010.tar.gz › OAA76622_1_165/msa_coverage.png]

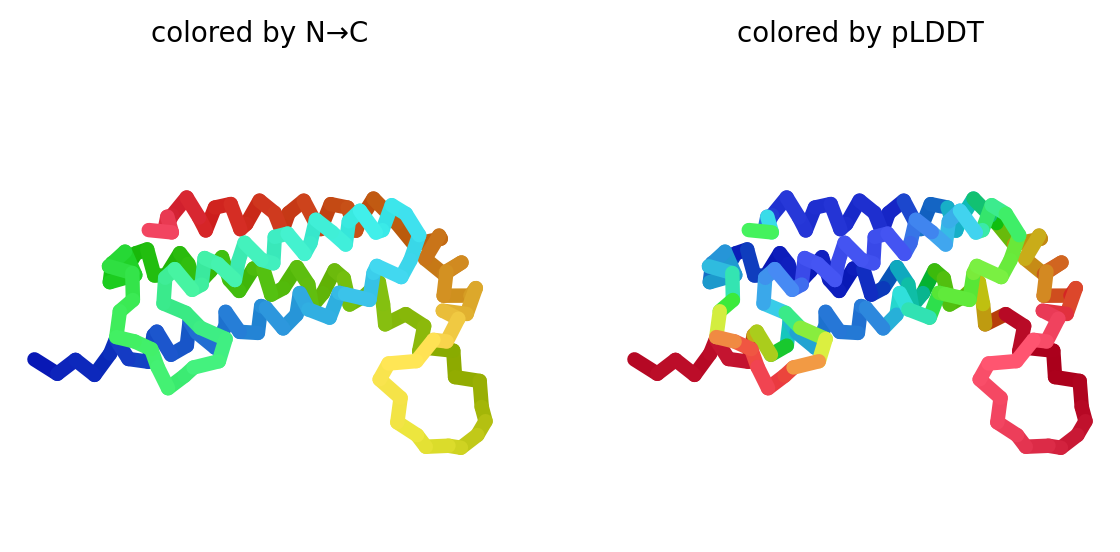

Supplement: S3 Data — Full AlphaFold2/ColabFold outputs. (GZ) [file pcbi.1010787.s010.tar.gz › OAA76622_1_165/rank_1_model_3_ptm_seed_0.png]

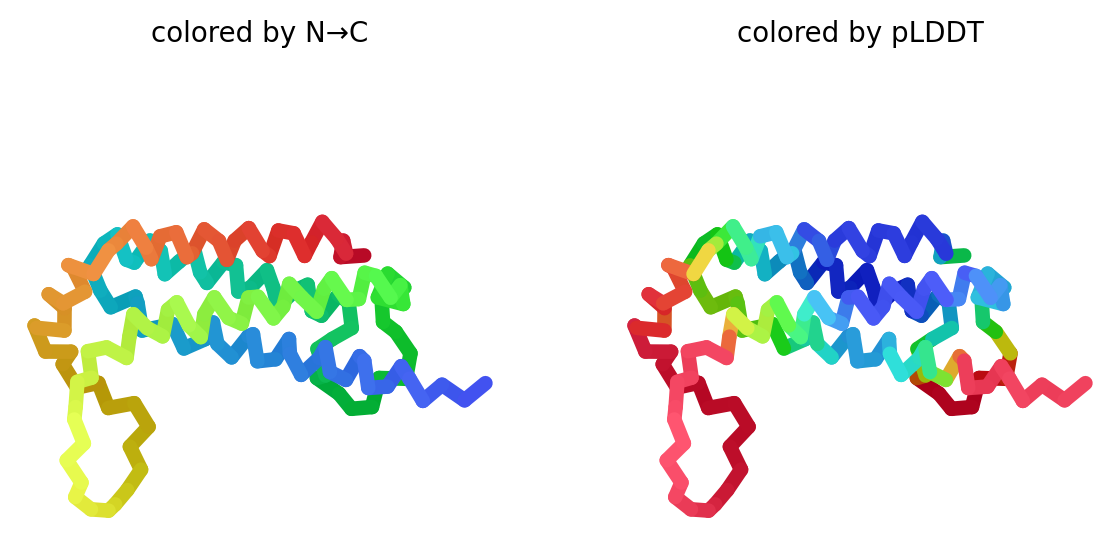

Supplement: S3 Data — Full AlphaFold2/ColabFold outputs. (GZ) [file pcbi.1010787.s010.tar.gz › OAA76622_1_165/rank_2_model_5_ptm_seed_0.png]

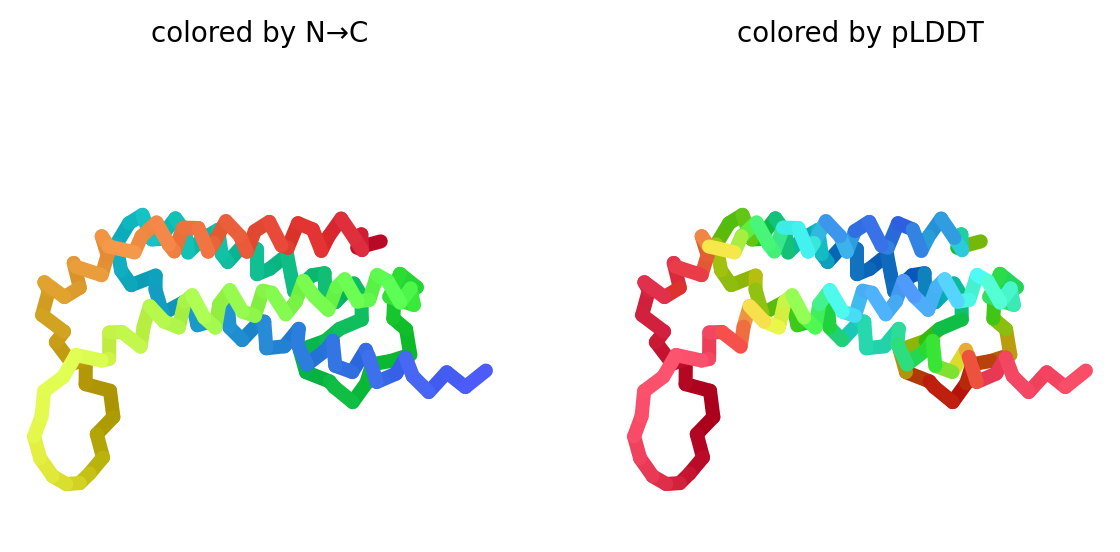

Supplement: S3 Data — Full AlphaFold2/ColabFold outputs. (GZ) [file pcbi.1010787.s010.tar.gz › OAA76622_1_165/rank_5_model_4_ptm_seed_0.png]

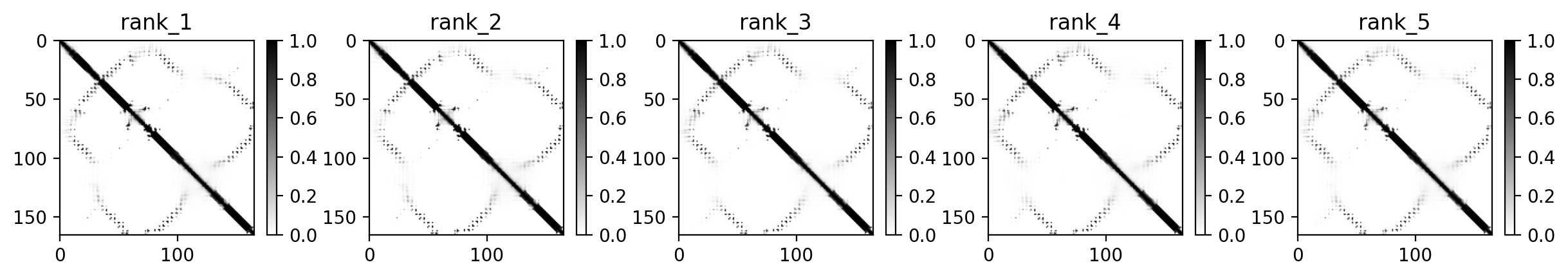

Supplement: S3 Data — Full AlphaFold2/ColabFold outputs. (GZ) [file pcbi.1010787.s010.tar.gz › OAA76622_1_165/predicted_contacts.png]

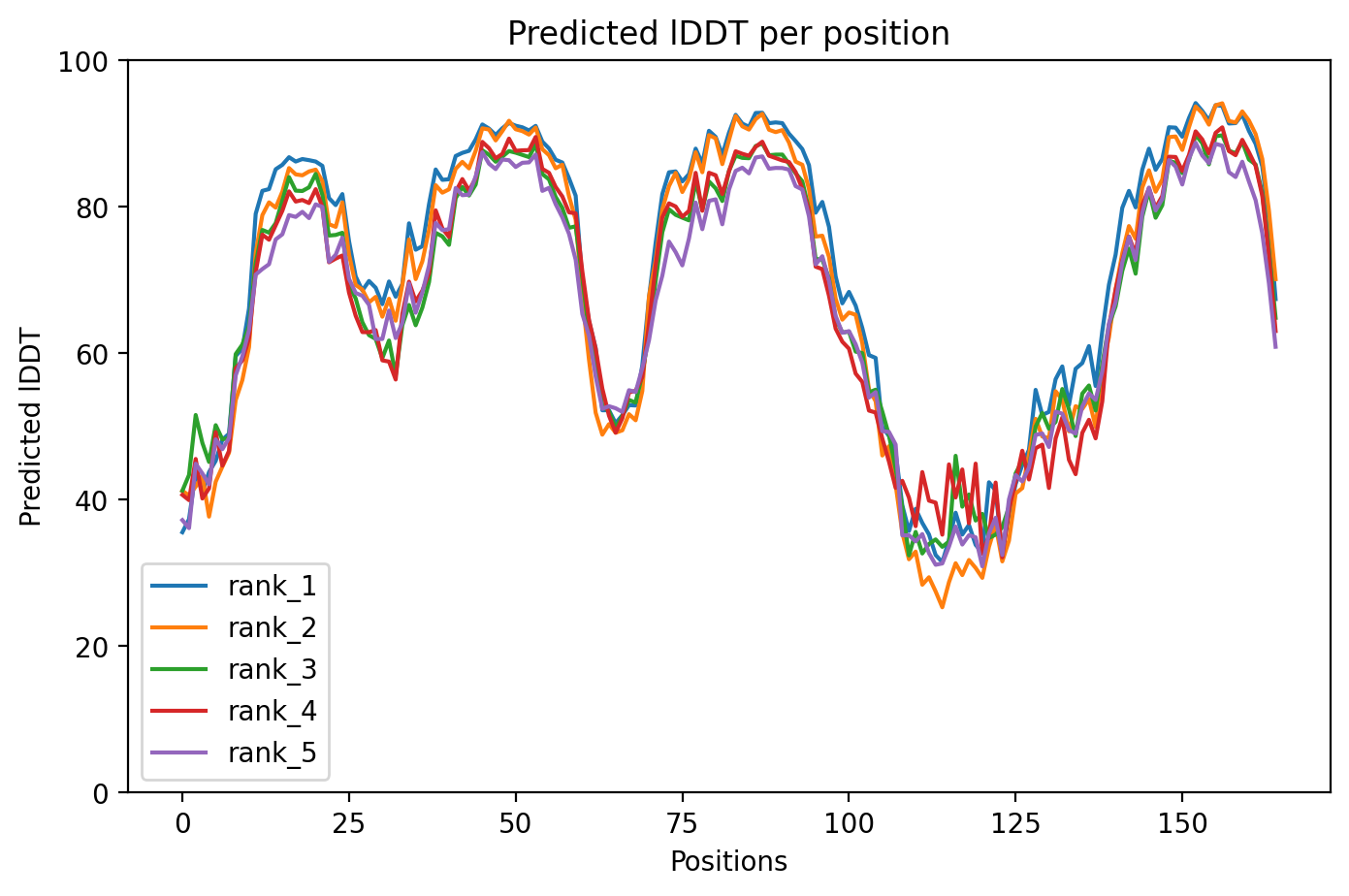

Supplement: S3 Data — Full AlphaFold2/ColabFold outputs. (GZ) [file pcbi.1010787.s010.tar.gz › OAA76622_1_165/predicted_LDDT.png]

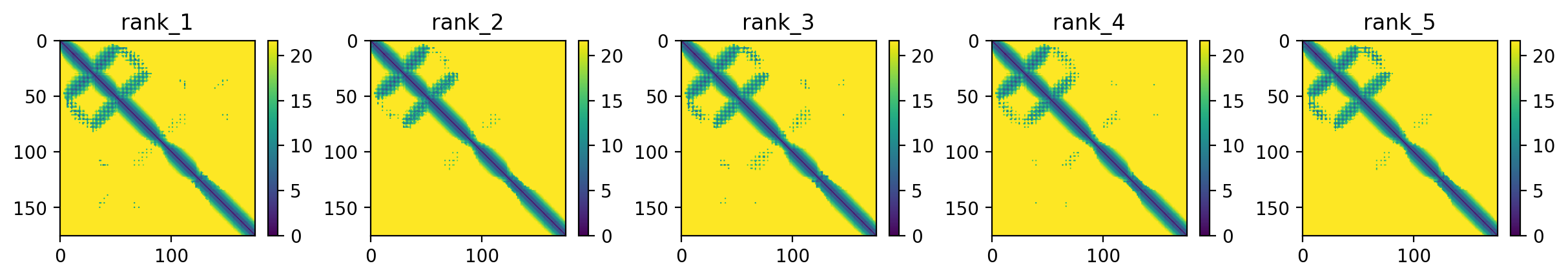

Supplement: S3 Data — Full AlphaFold2/ColabFold outputs. (GZ) [file pcbi.1010787.s010.tar.gz › OBZ65626_1_175/predicted_distogram.png]

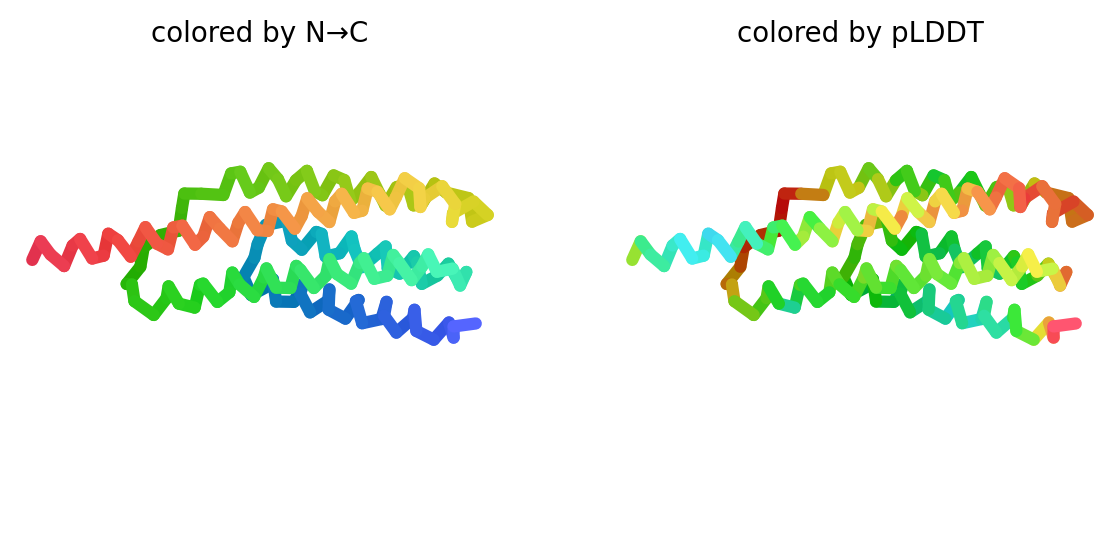

Supplement: S3 Data — Full AlphaFold2/ColabFold outputs. (GZ) [file pcbi.1010787.s010.tar.gz › OBZ65626_1_175/rank_3_model_2_ptm_seed_0.png]

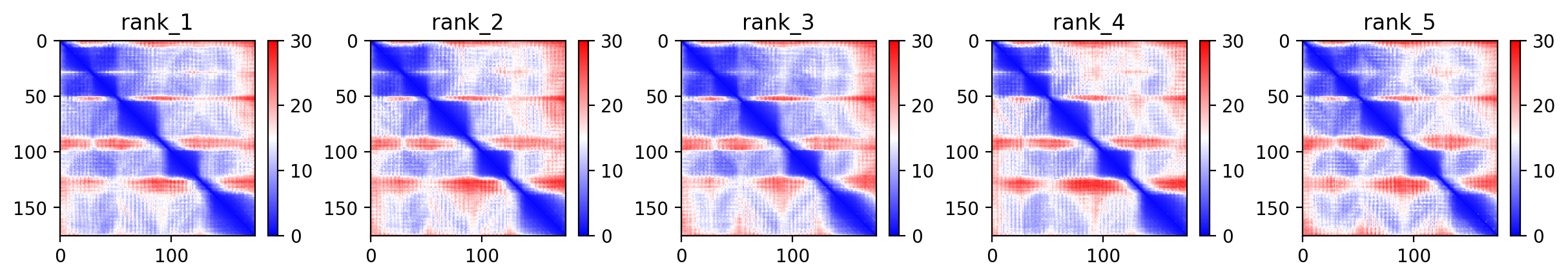

Supplement: S3 Data — Full AlphaFold2/ColabFold outputs. (GZ) [file pcbi.1010787.s010.tar.gz › OBZ65626_1_175/predicted_alignment_error.png]

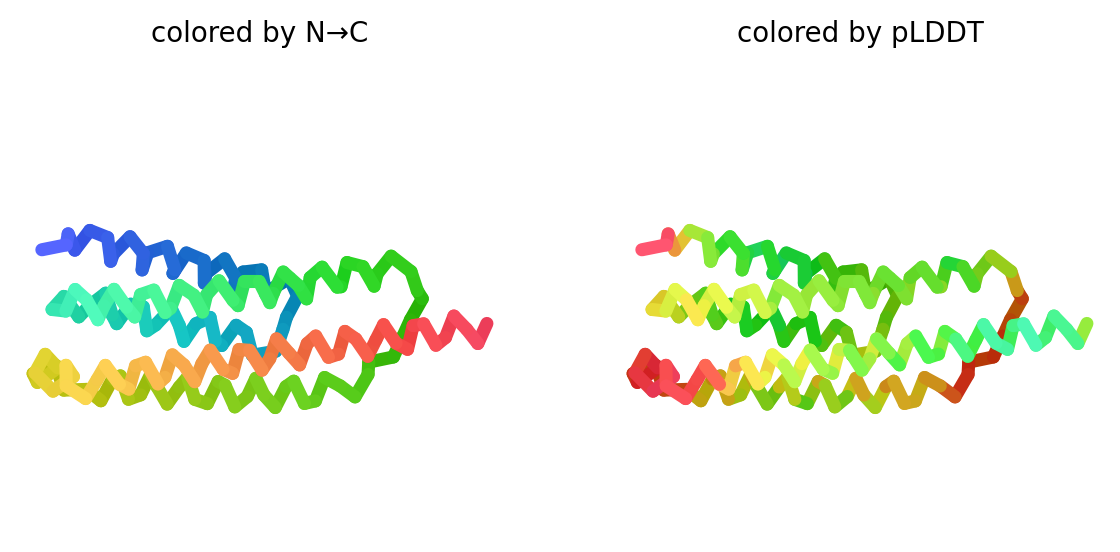

Supplement: S3 Data — Full AlphaFold2/ColabFold outputs. (GZ) [file pcbi.1010787.s010.tar.gz › OBZ65626_1_175/rank_5_model_1_ptm_seed_0.png]

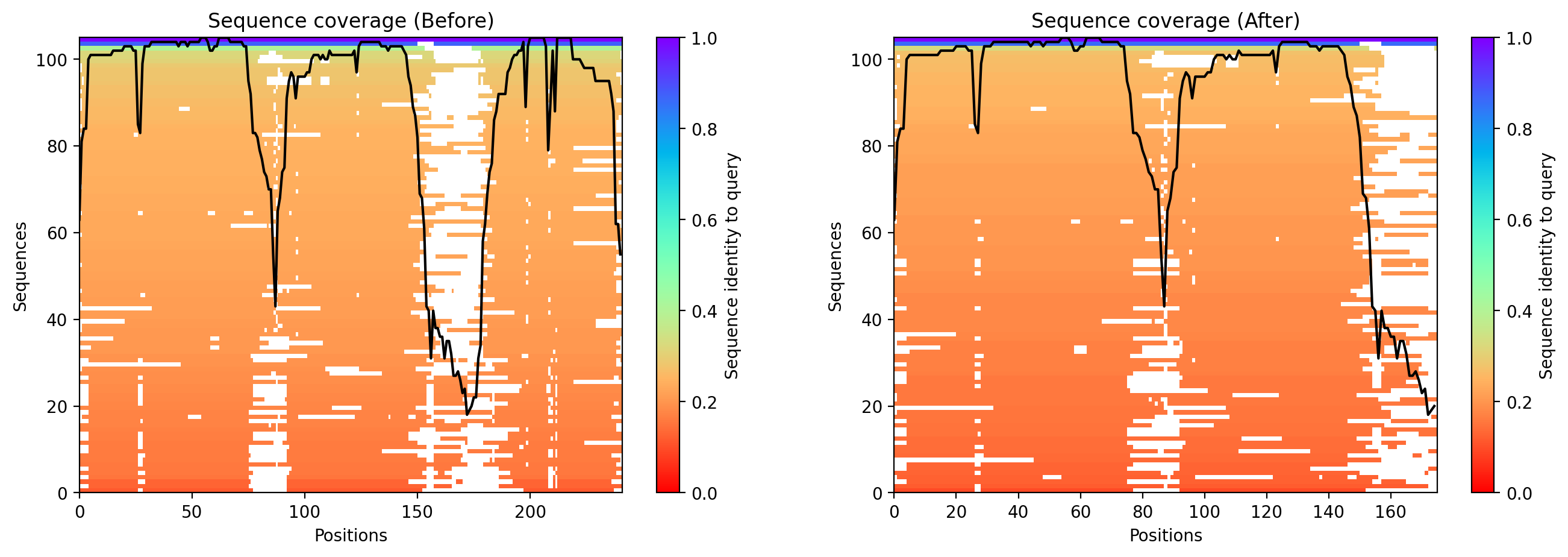

Supplement: S3 Data — Full AlphaFold2/ColabFold outputs. (GZ) [file pcbi.1010787.s010.tar.gz › OBZ65626_1_175/msa_coverage.filtered.png]

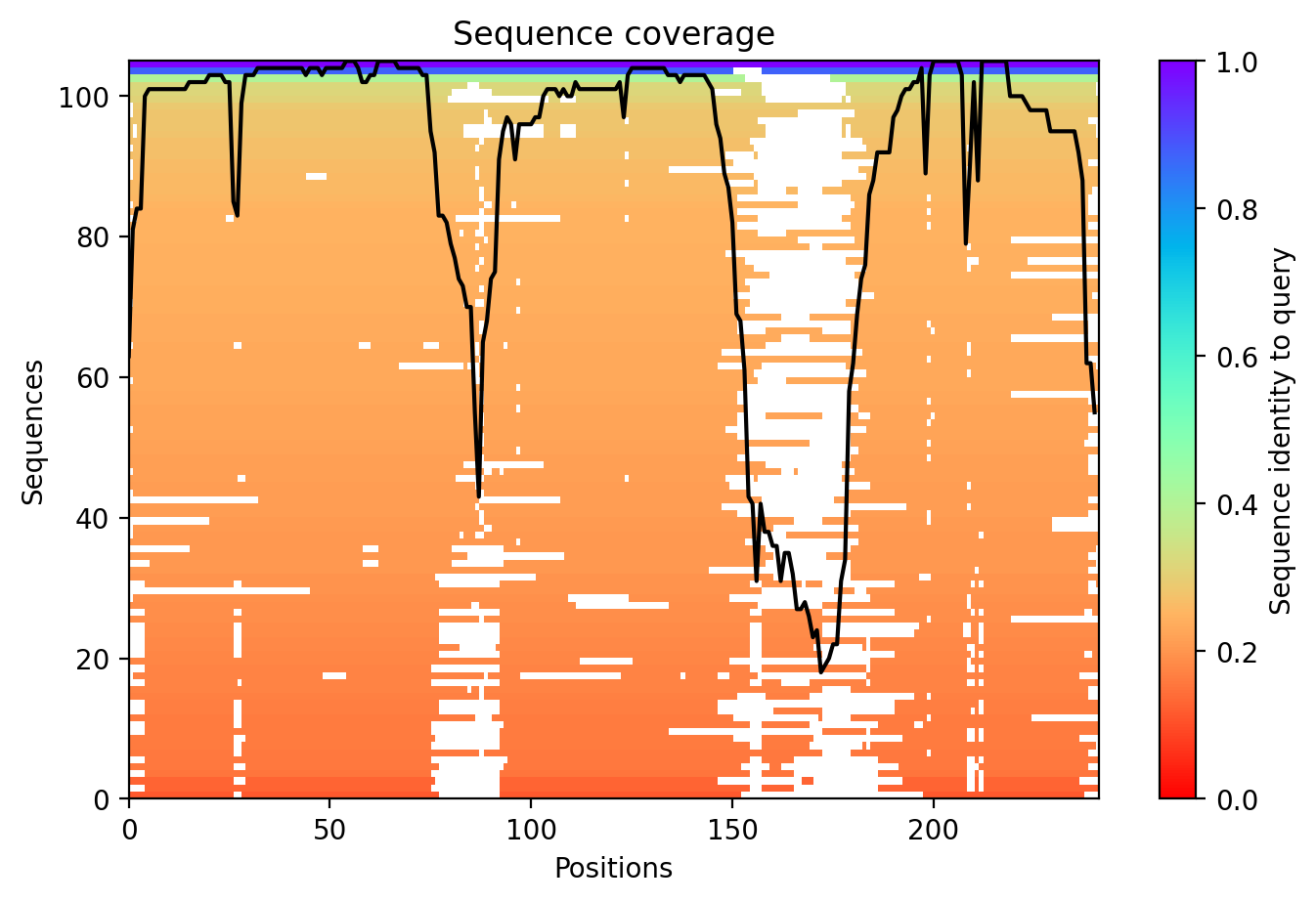

Supplement: S3 Data — Full AlphaFold2/ColabFold outputs. (GZ) [file pcbi.1010787.s010.tar.gz › OBZ65626_1_175/msa_coverage.png]

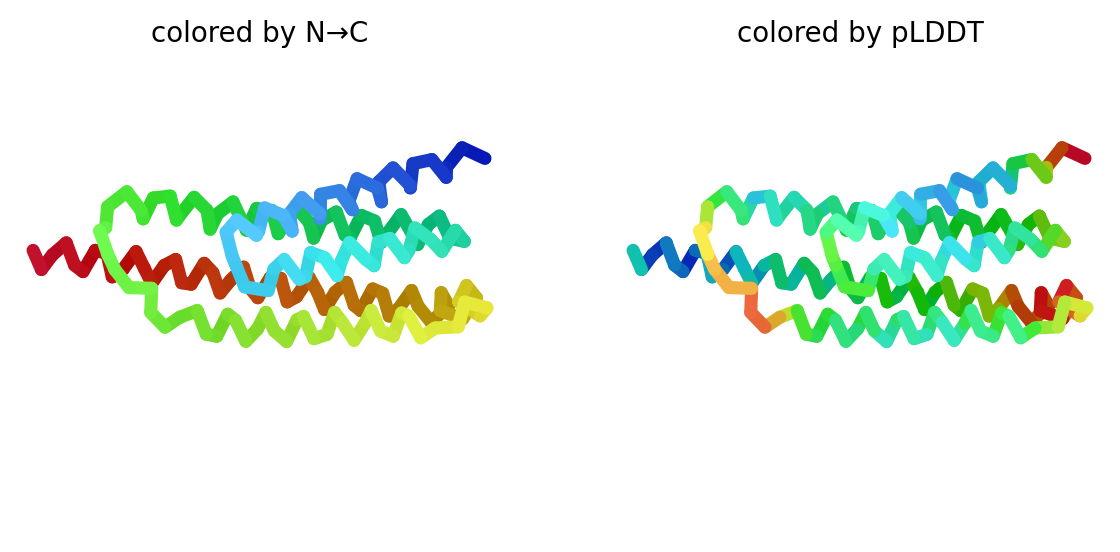

Supplement: S3 Data — Full AlphaFold2/ColabFold outputs. (GZ) [file pcbi.1010787.s010.tar.gz › OBZ65626_1_175/rank_1_model_3_ptm_seed_0.png]

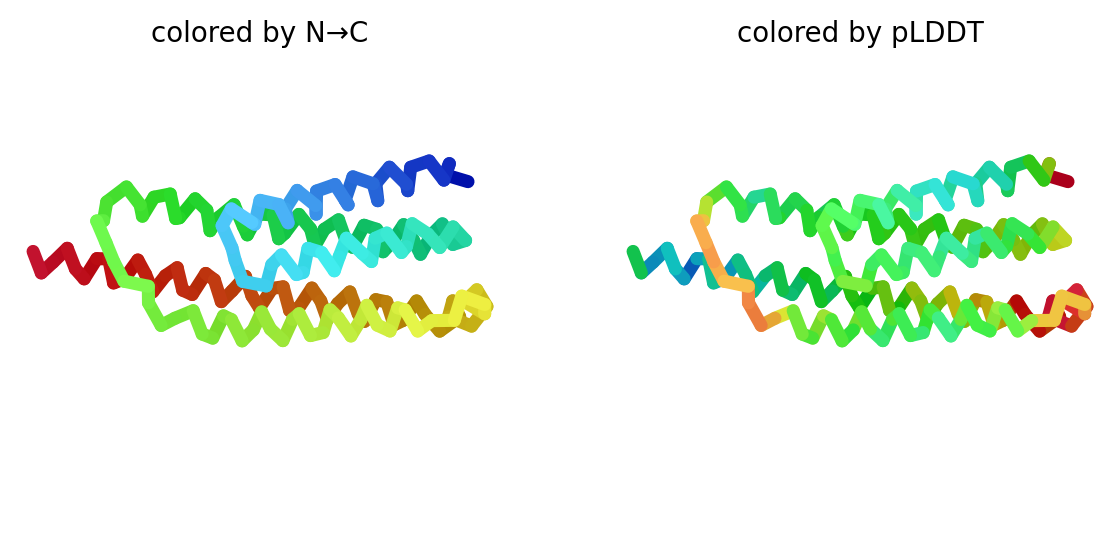

Supplement: S3 Data — Full AlphaFold2/ColabFold outputs. (GZ) [file pcbi.1010787.s010.tar.gz › OBZ65626_1_175/rank_2_model_4_ptm_seed_0.png]

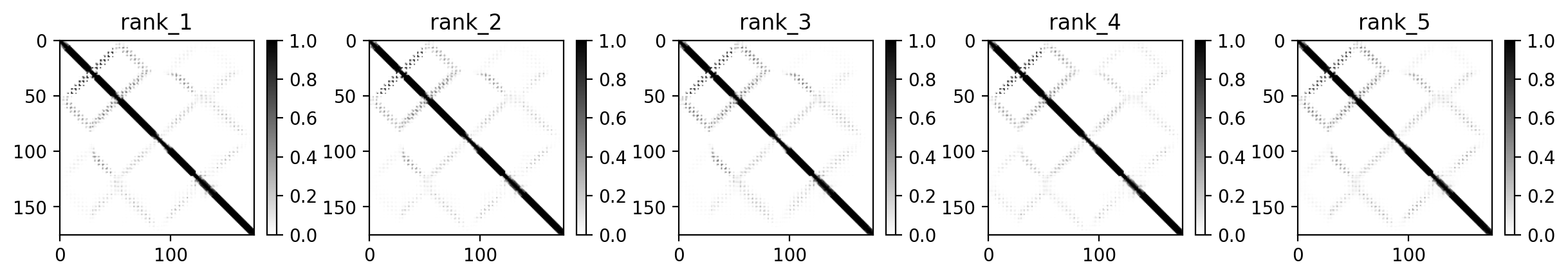

Supplement: S3 Data — Full AlphaFold2/ColabFold outputs. (GZ) [file pcbi.1010787.s010.tar.gz › OBZ65626_1_175/predicted_contacts.png]

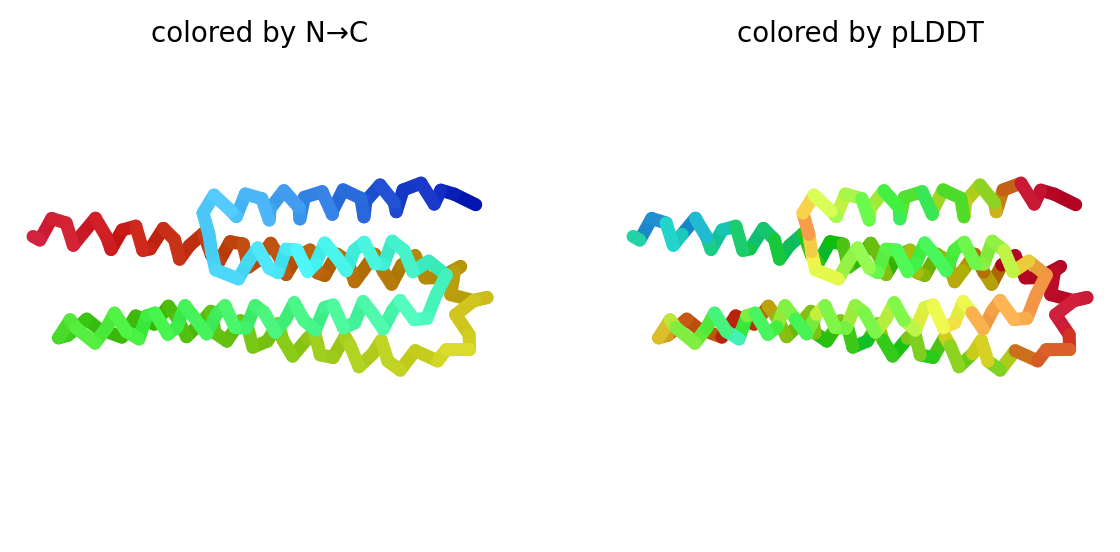

Supplement: S3 Data — Full AlphaFold2/ColabFold outputs. (GZ) [file pcbi.1010787.s010.tar.gz › OBZ65626_1_175/rank_4_model_5_ptm_seed_0.png]

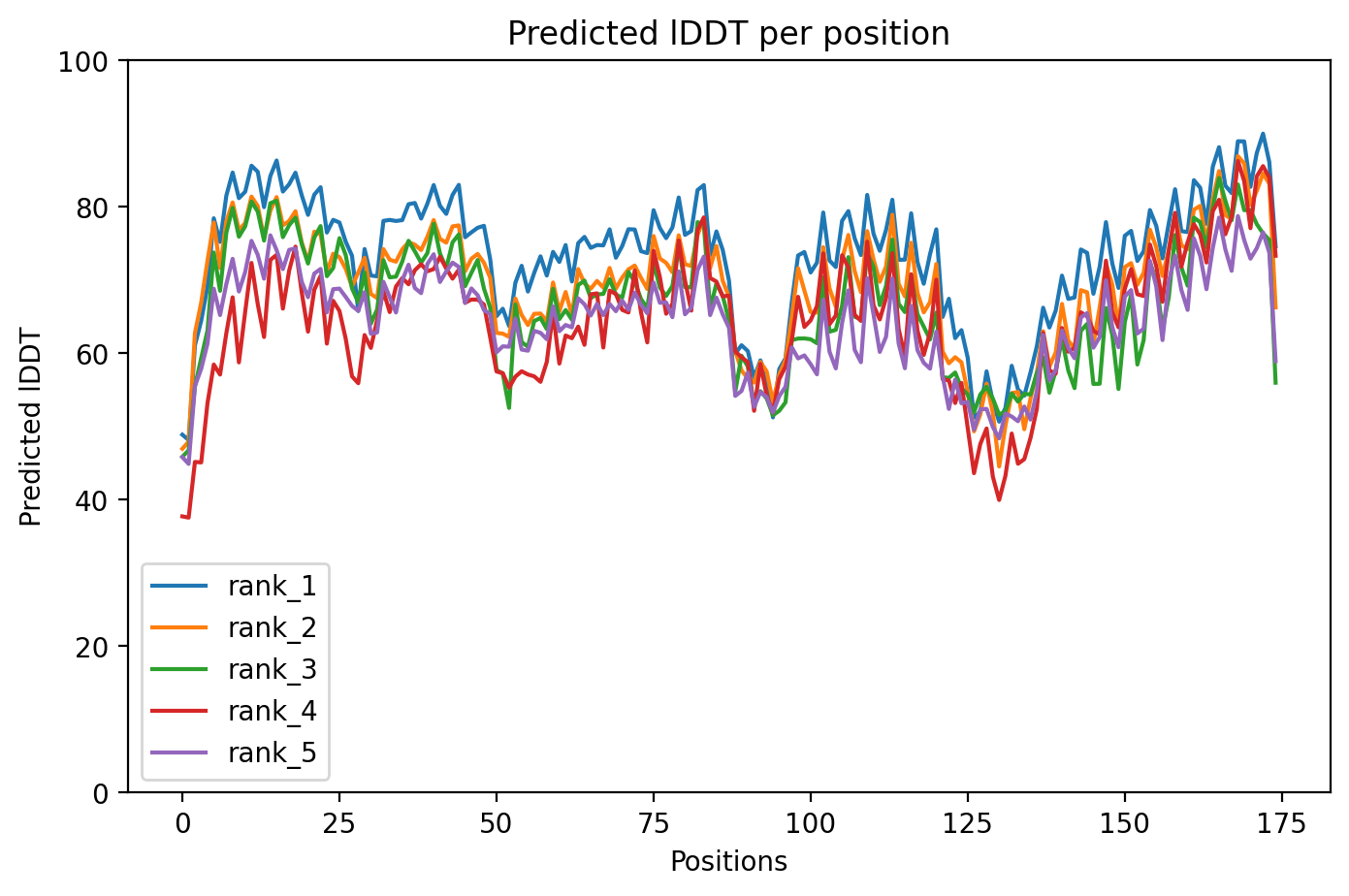

Supplement: S3 Data — Full AlphaFold2/ColabFold outputs. (GZ) [file pcbi.1010787.s010.tar.gz › OBZ65626_1_175/predicted_LDDT.png]

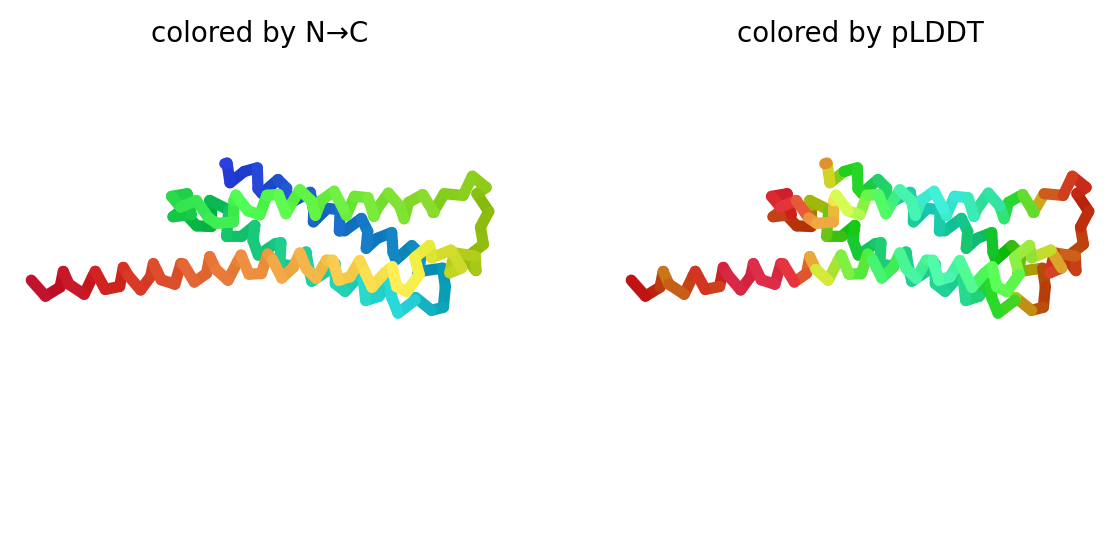

Supplement: S3 Data — Full AlphaFold2/ColabFold outputs. (GZ) [file pcbi.1010787.s010.tar.gz › PWW74656_1_165/rank_4_model_4_ptm_seed_0.png]

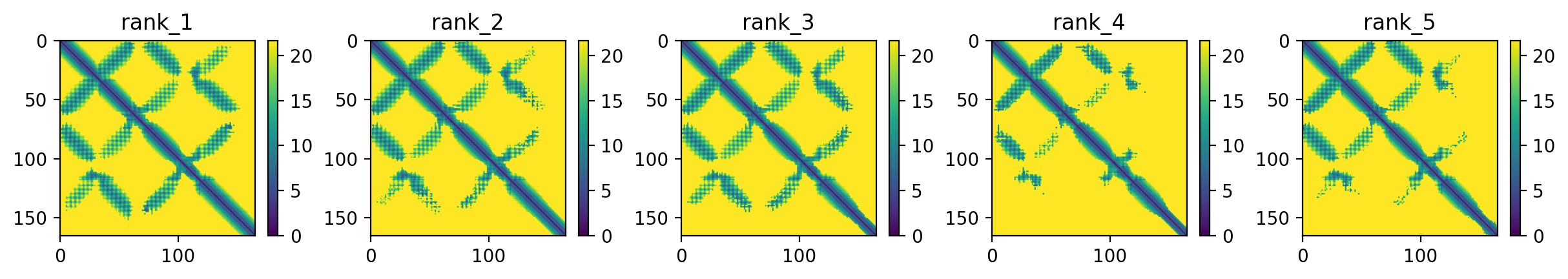

Supplement: S3 Data — Full AlphaFold2/ColabFold outputs. (GZ) [file pcbi.1010787.s010.tar.gz › PWW74656_1_165/predicted_distogram.png]

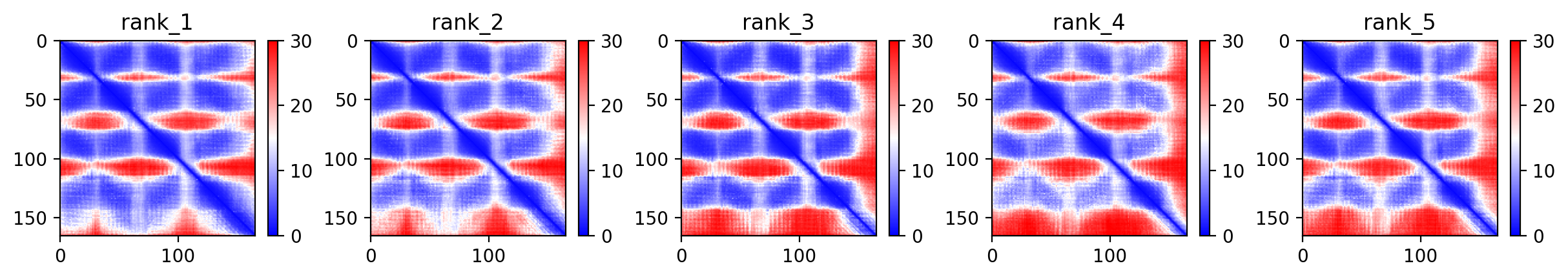

Supplement: S3 Data — Full AlphaFold2/ColabFold outputs. (GZ) [file pcbi.1010787.s010.tar.gz › PWW74656_1_165/predicted_alignment_error.png]

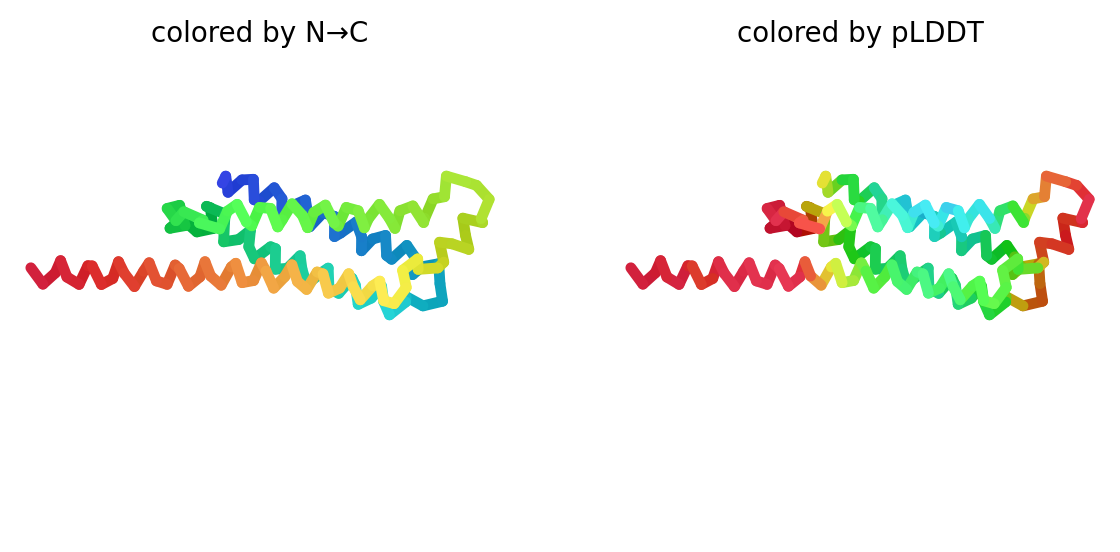

Supplement: S3 Data — Full AlphaFold2/ColabFold outputs. (GZ) [file pcbi.1010787.s010.tar.gz › PWW74656_1_165/rank_5_model_2_ptm_seed_0.png]

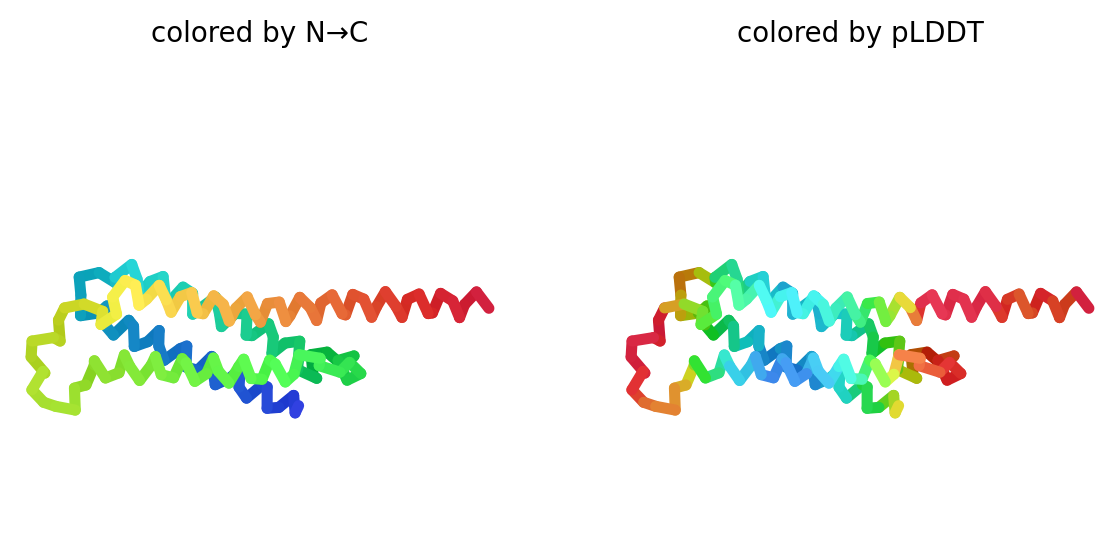

Supplement: S3 Data — Full AlphaFold2/ColabFold outputs. (GZ) [file pcbi.1010787.s010.tar.gz › PWW74656_1_165/rank_3_model_1_ptm_seed_0.png]

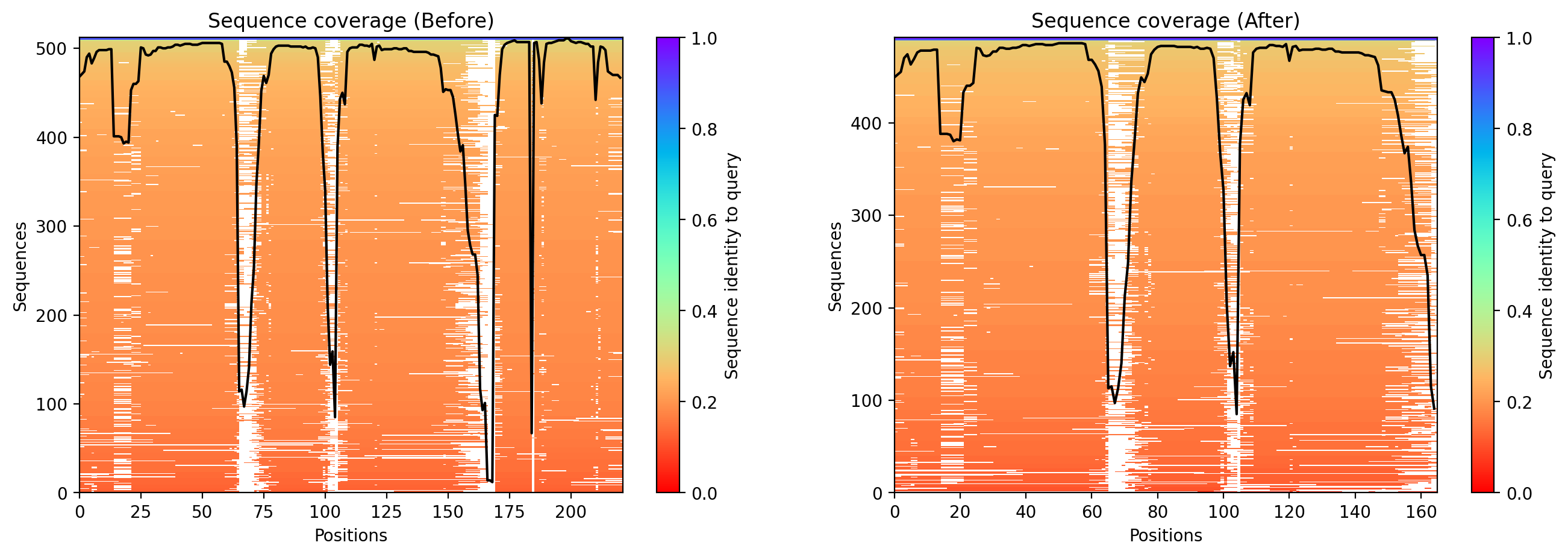

Supplement: S3 Data — Full AlphaFold2/ColabFold outputs. (GZ) [file pcbi.1010787.s010.tar.gz › PWW74656_1_165/msa_coverage.filtered.png]

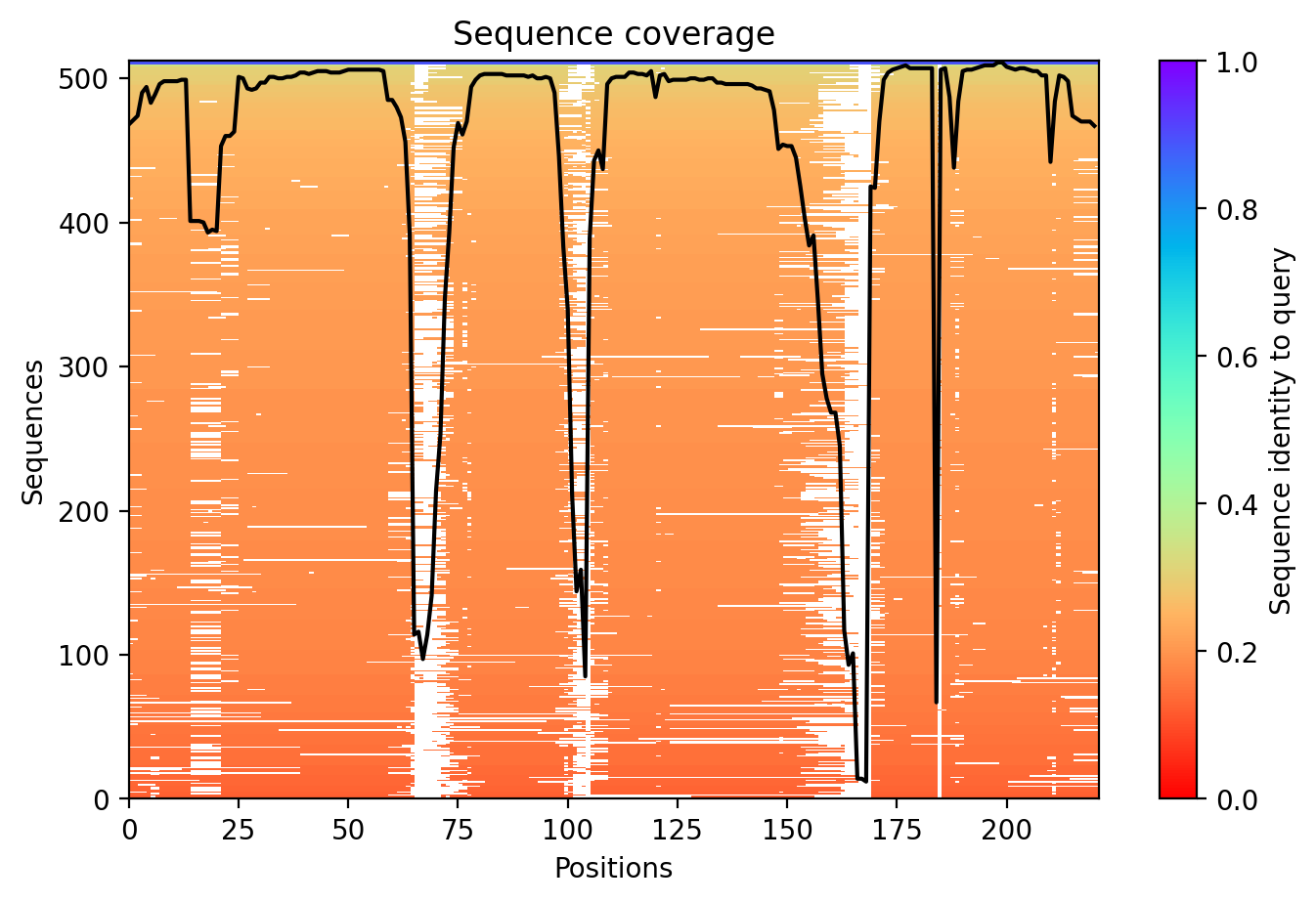

Supplement: S3 Data — Full AlphaFold2/ColabFold outputs. (GZ) [file pcbi.1010787.s010.tar.gz › PWW74656_1_165/msa_coverage.png]

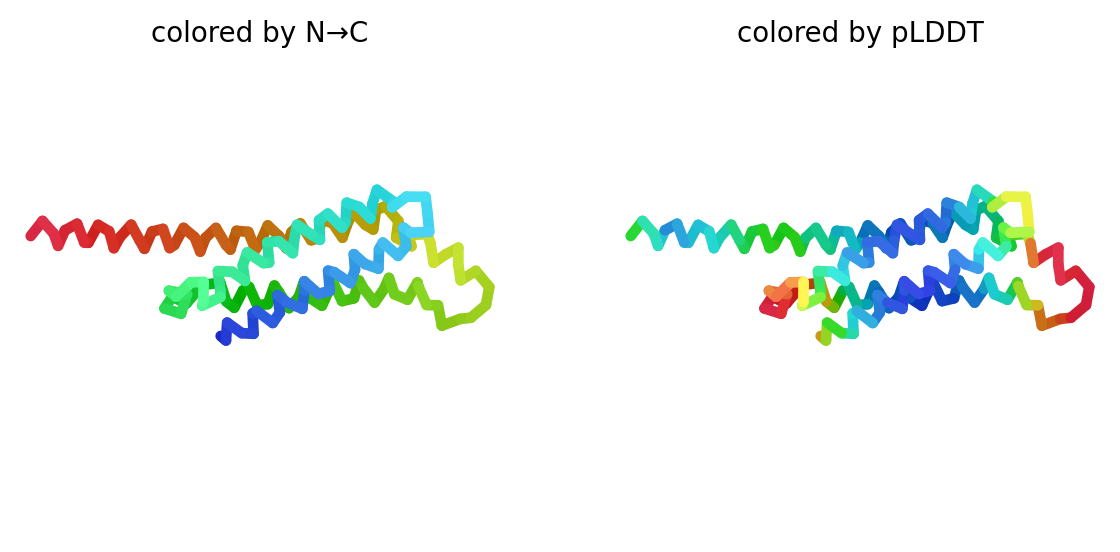

Supplement: S3 Data — Full AlphaFold2/ColabFold outputs. (GZ) [file pcbi.1010787.s010.tar.gz › PWW74656_1_165/rank_1_model_3_ptm_seed_0.png]

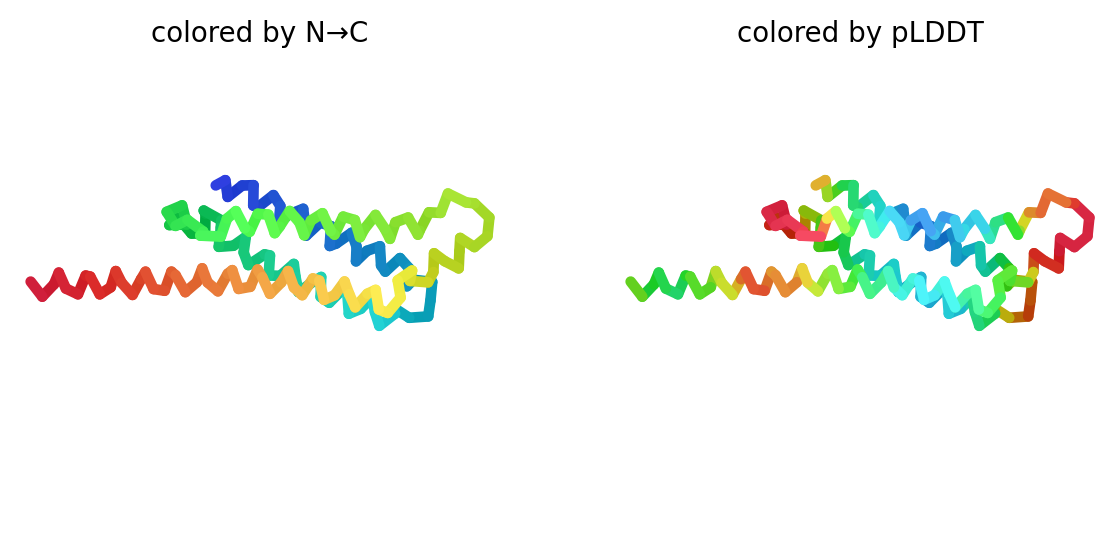

Supplement: S3 Data — Full AlphaFold2/ColabFold outputs. (GZ) [file pcbi.1010787.s010.tar.gz › PWW74656_1_165/rank_2_model_5_ptm_seed_0.png]

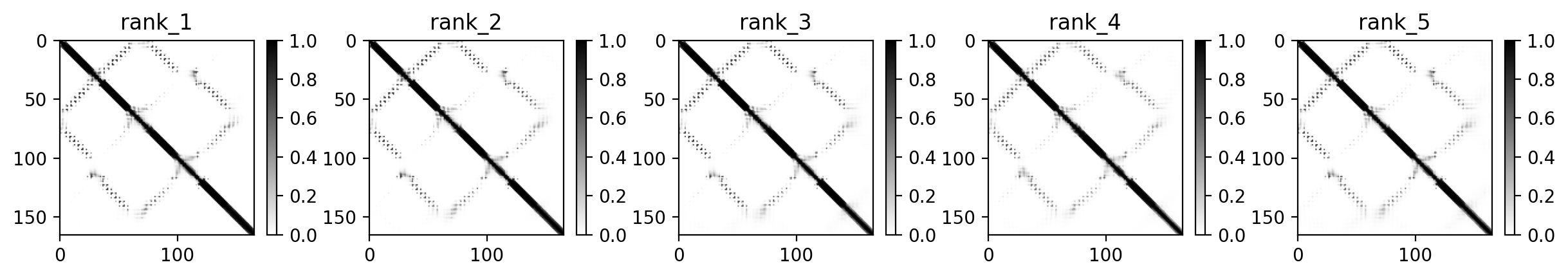

Supplement: S3 Data — Full AlphaFold2/ColabFold outputs. (GZ) [file pcbi.1010787.s010.tar.gz › PWW74656_1_165/predicted_contacts.png]

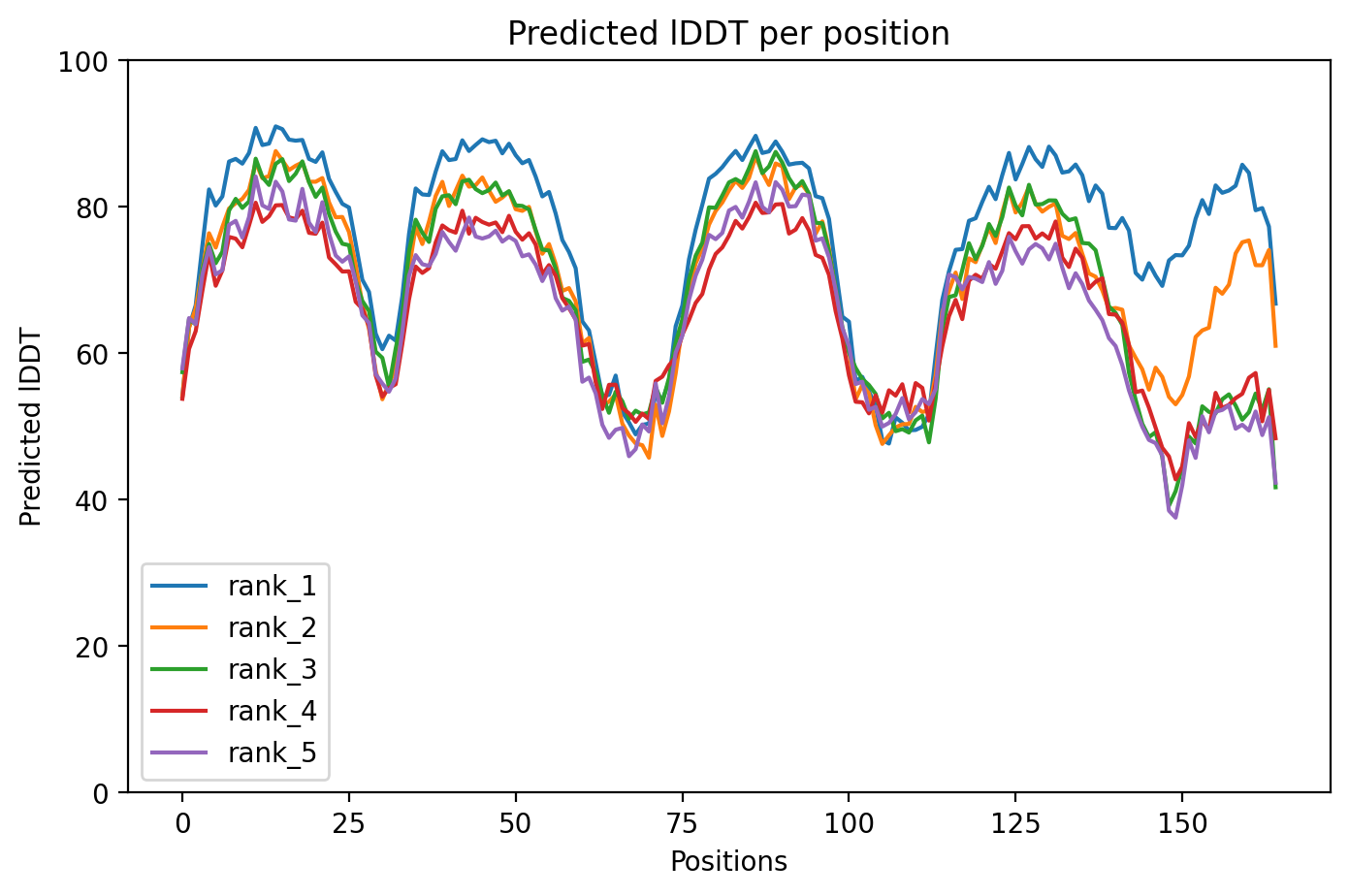

Supplement: S3 Data — Full AlphaFold2/ColabFold outputs. (GZ) [file pcbi.1010787.s010.tar.gz › PWW74656_1_165/predicted_LDDT.png]

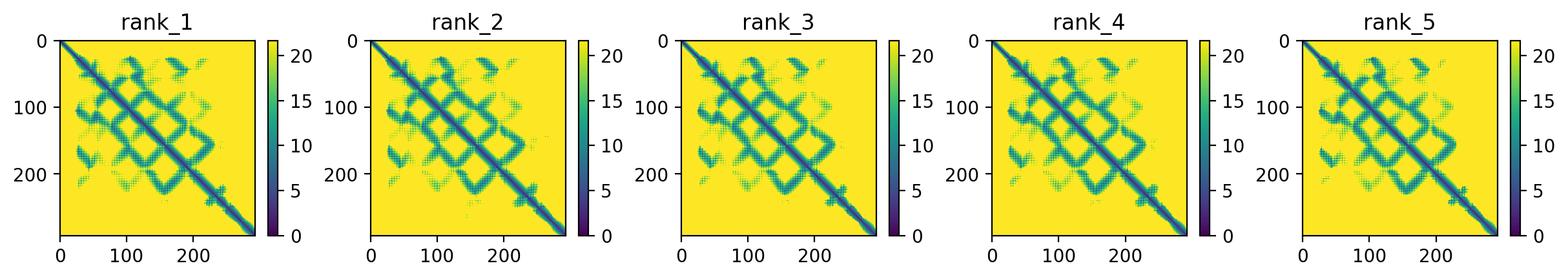

Supplement: S3 Data — Full AlphaFold2/ColabFold outputs. (GZ) [file pcbi.1010787.s010.tar.gz › RAK81296_1_292/predicted_distogram.png]

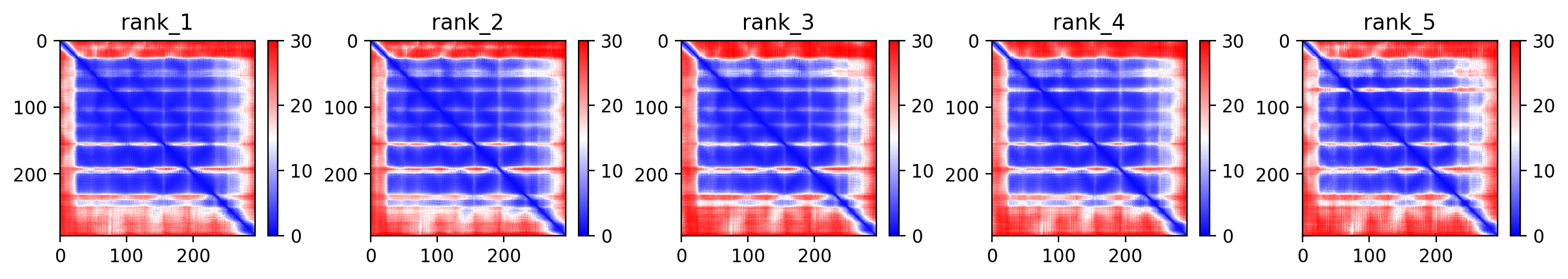

Supplement: S3 Data — Full AlphaFold2/ColabFold outputs. (GZ) [file pcbi.1010787.s010.tar.gz › RAK81296_1_292/predicted_alignment_error.png]

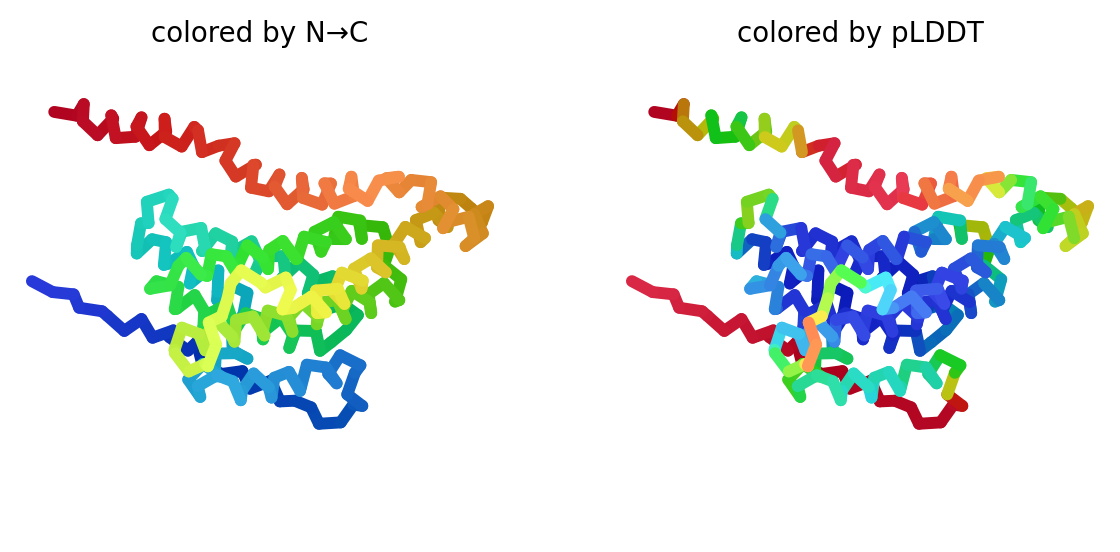

Supplement: S3 Data — Full AlphaFold2/ColabFold outputs. (GZ) [file pcbi.1010787.s010.tar.gz › RAK81296_1_292/rank_5_model_1_ptm_seed_0.png]

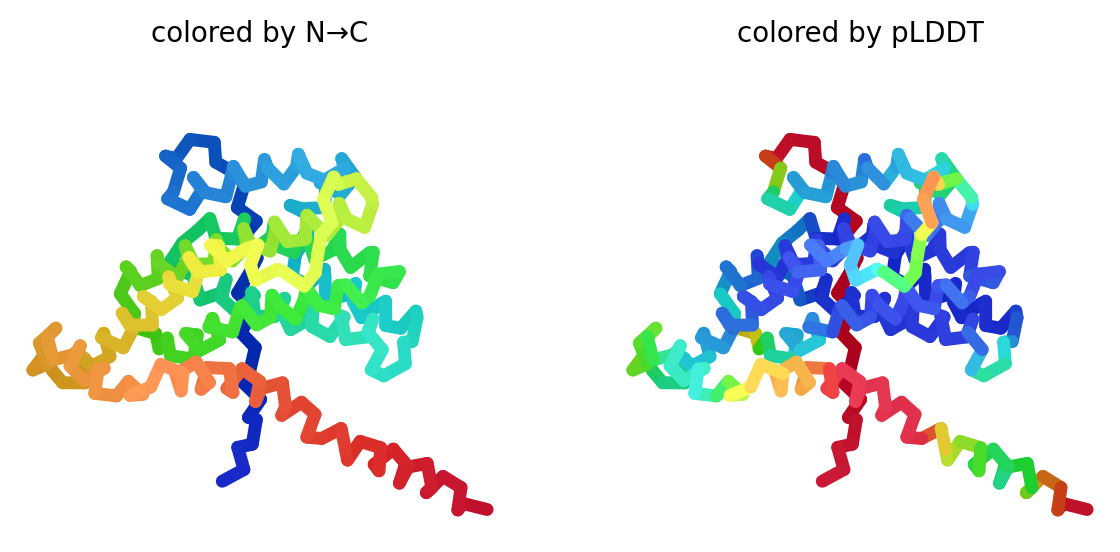

Supplement: S3 Data — Full AlphaFold2/ColabFold outputs. (GZ) [file pcbi.1010787.s010.tar.gz › RAK81296_1_292/rank_4_model_2_ptm_seed_0.png]

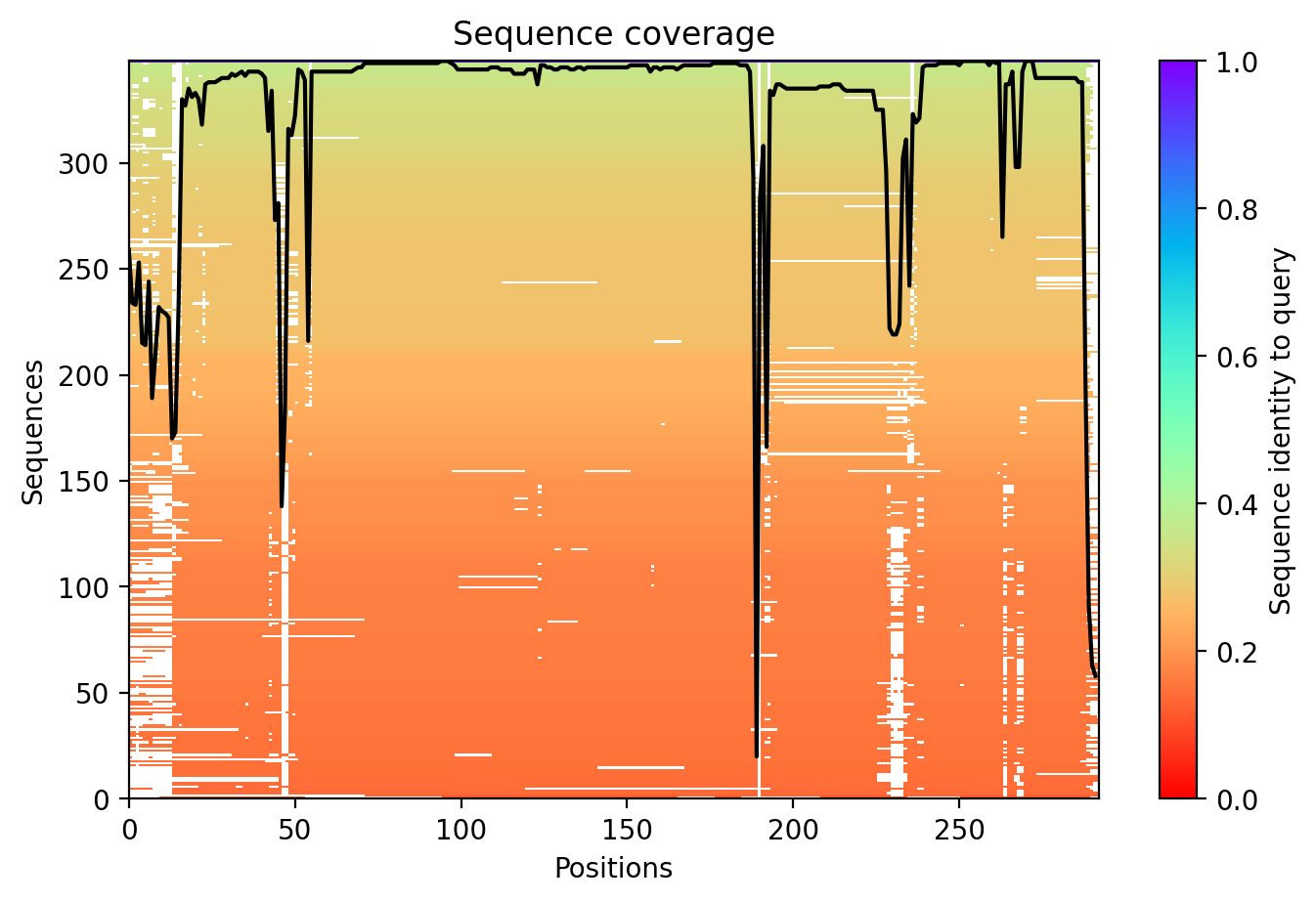

Supplement: S3 Data — Full AlphaFold2/ColabFold outputs. (GZ) [file pcbi.1010787.s010.tar.gz › RAK81296_1_292/msa_coverage.png]

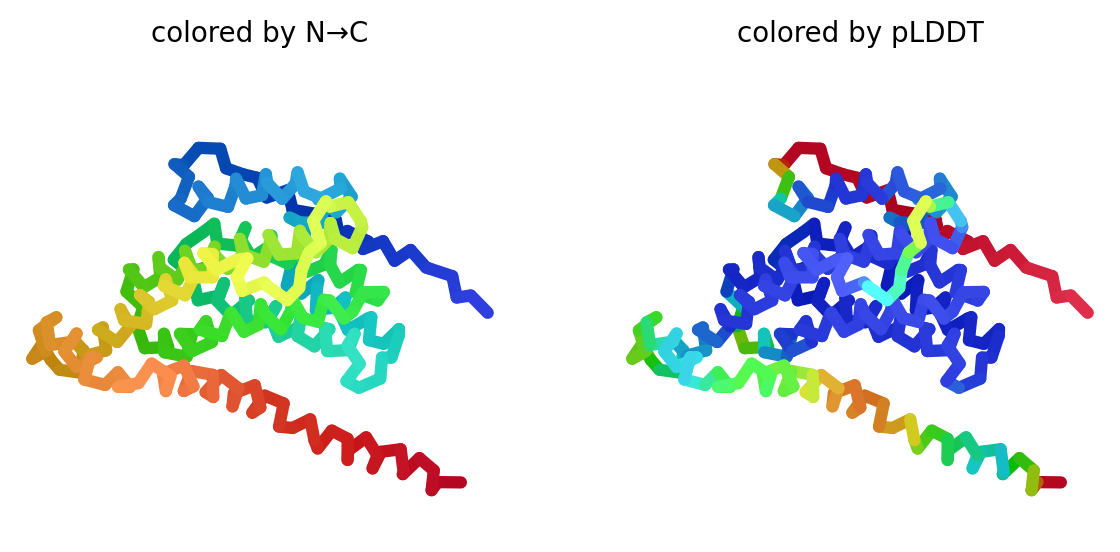

Supplement: S3 Data — Full AlphaFold2/ColabFold outputs. (GZ) [file pcbi.1010787.s010.tar.gz › RAK81296_1_292/rank_1_model_3_ptm_seed_0.png]

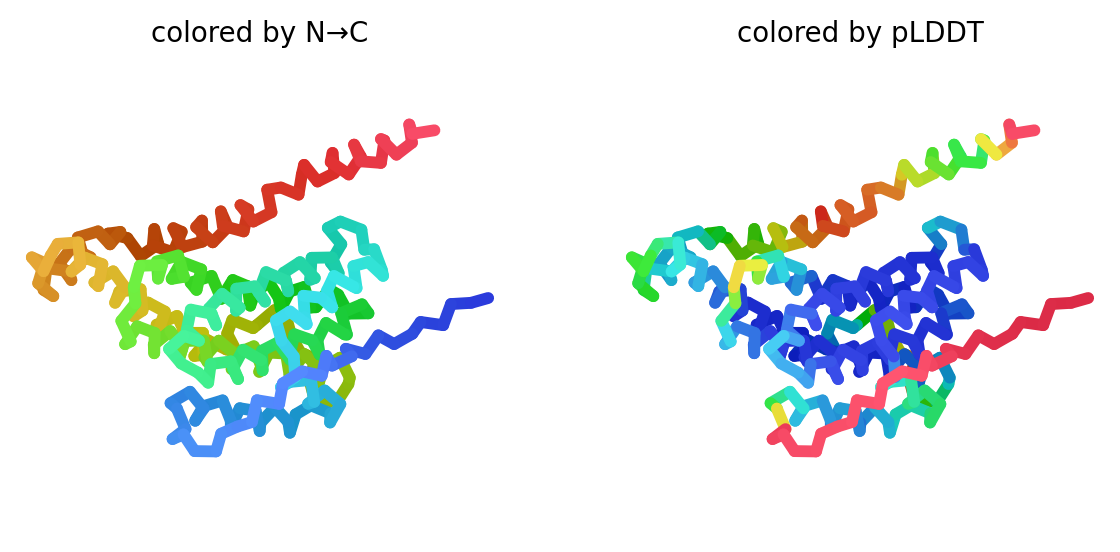

Supplement: S3 Data — Full AlphaFold2/ColabFold outputs. (GZ) [file pcbi.1010787.s010.tar.gz › RAK81296_1_292/rank_2_model_5_ptm_seed_0.png]

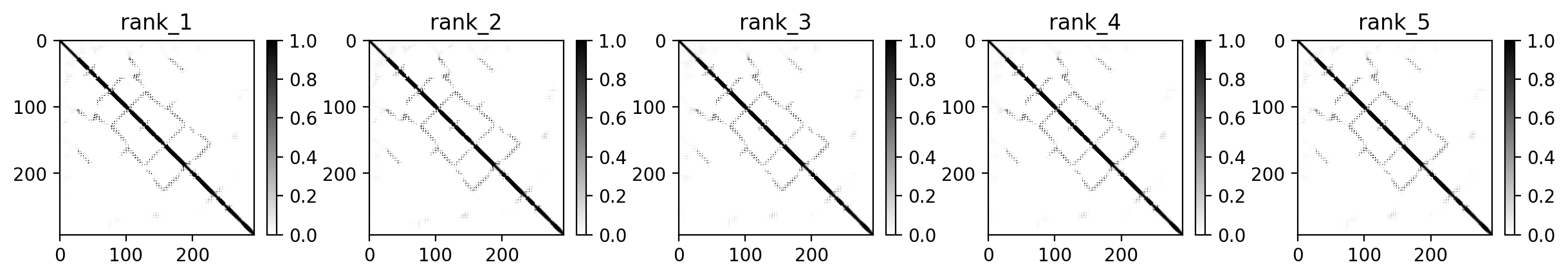

Supplement: S3 Data — Full AlphaFold2/ColabFold outputs. (GZ) [file pcbi.1010787.s010.tar.gz › RAK81296_1_292/predicted_contacts.png]

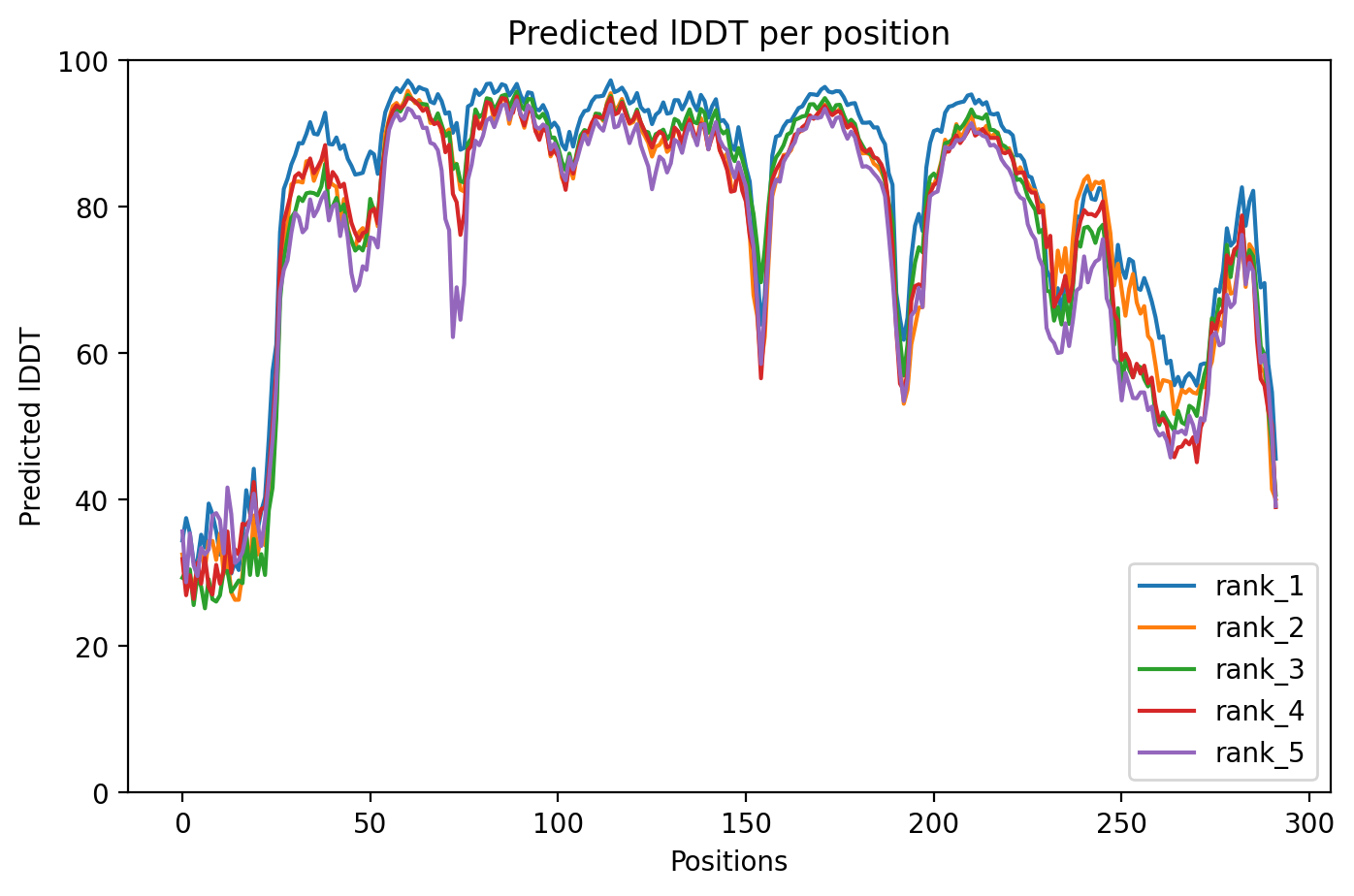

Supplement: S3 Data — Full AlphaFold2/ColabFold outputs. (GZ) [file pcbi.1010787.s010.tar.gz › RAK81296_1_292/predicted_LDDT.png]

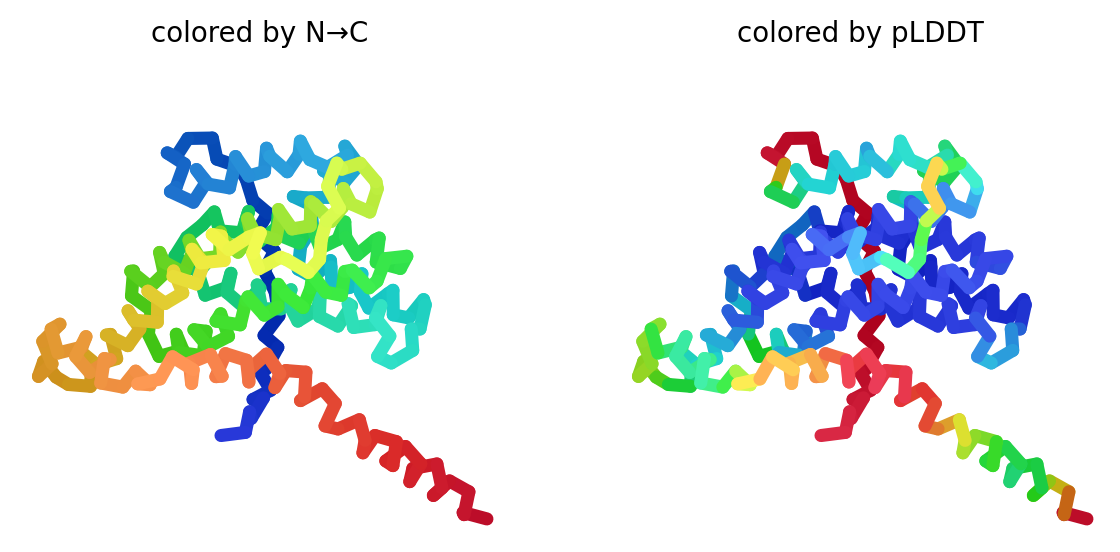

Supplement: S3 Data — Full AlphaFold2/ColabFold outputs. (GZ) [file pcbi.1010787.s010.tar.gz › RAK81296_1_292/rank_3_model_4_ptm_seed_0.png]

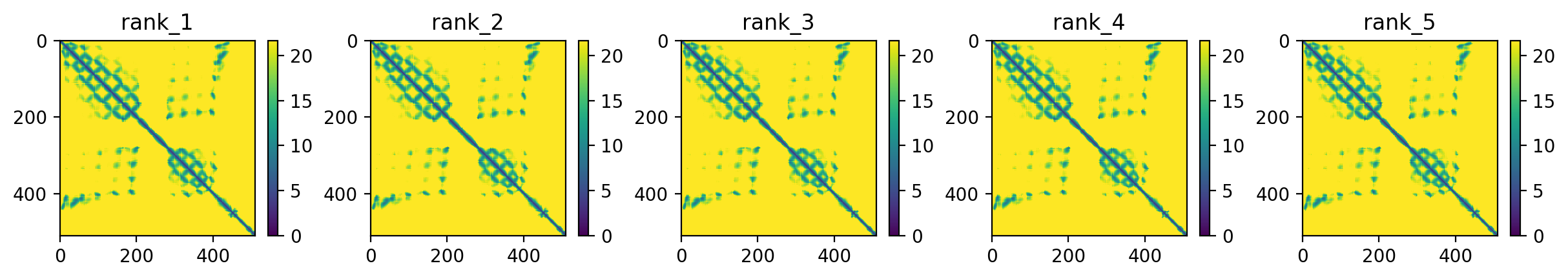

Supplement: S4 Data — Full AlphaFold2/ColabFold outputs. (GZ) [file pcbi.1010787.s011.tar.gz › KEY84097_1_509/predicted_distogram.png]

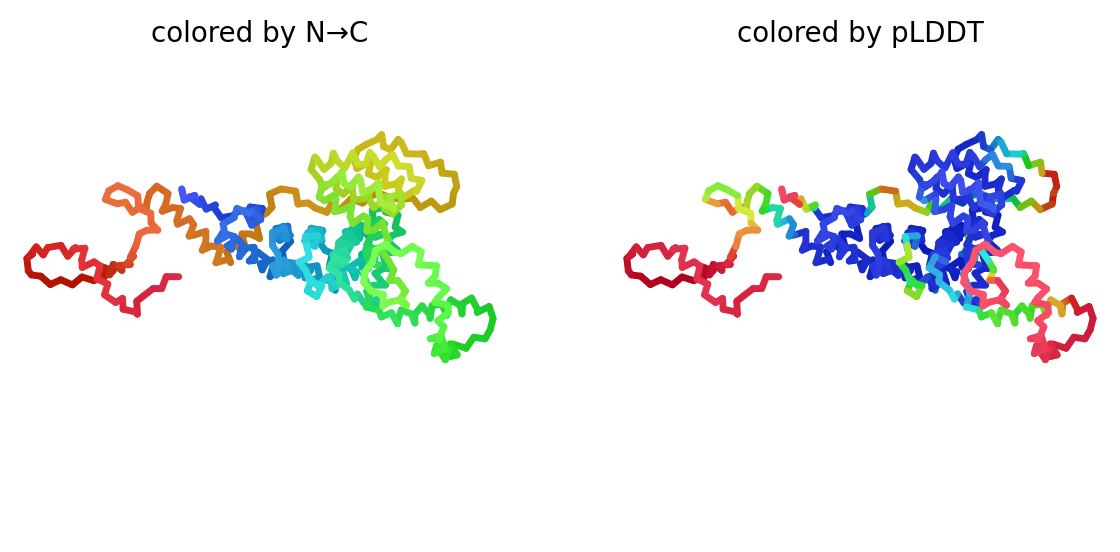

Supplement: S4 Data — Full AlphaFold2/ColabFold outputs. (GZ) [file pcbi.1010787.s011.tar.gz › KEY84097_1_509/rank_3_model_2_ptm_seed_0.png]

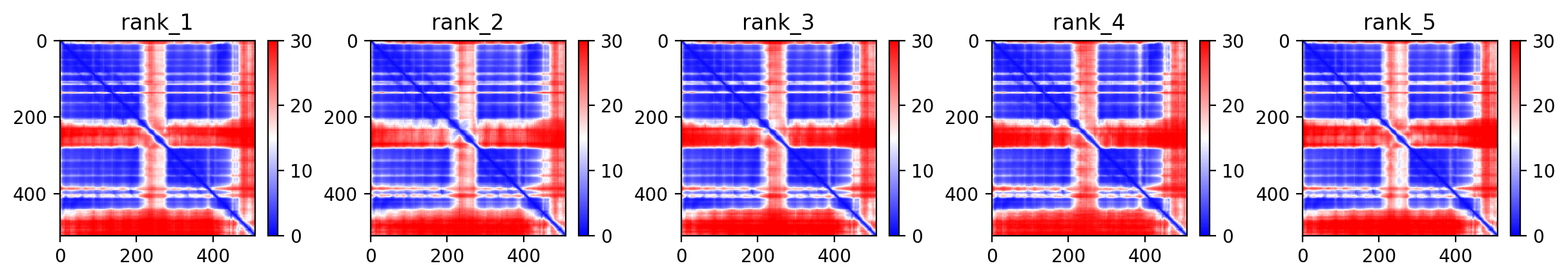

Supplement: S4 Data — Full AlphaFold2/ColabFold outputs. (GZ) [file pcbi.1010787.s011.tar.gz › KEY84097_1_509/predicted_alignment_error.png]

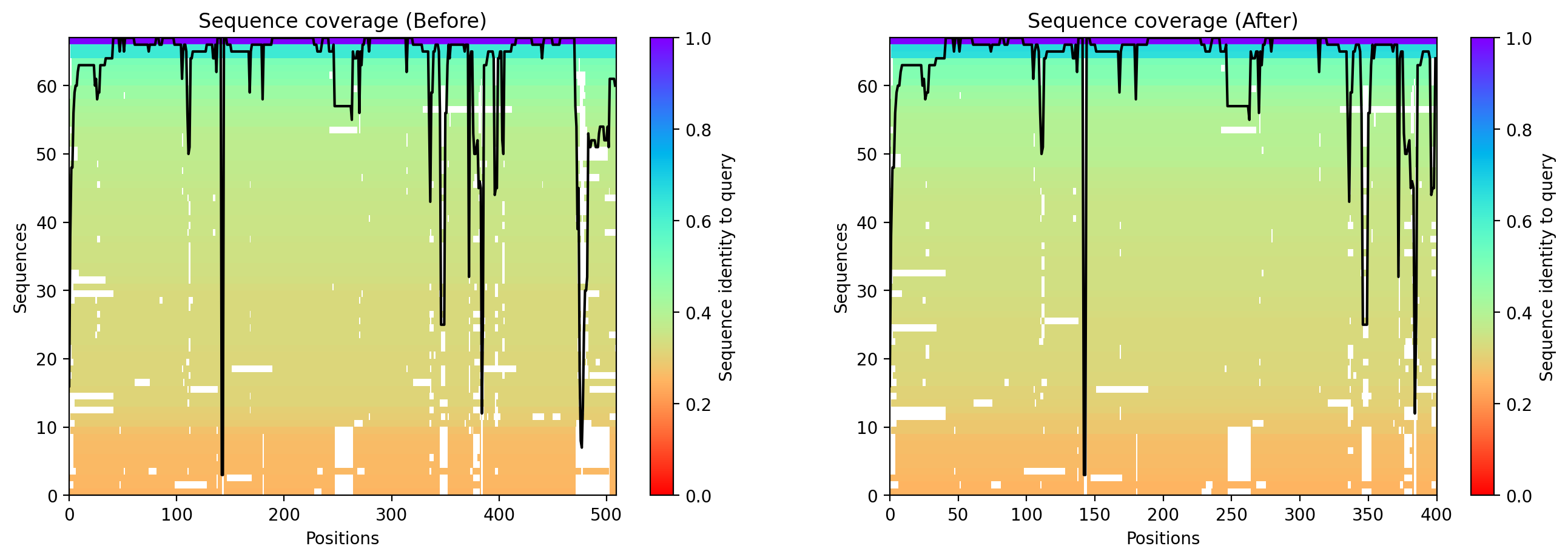

Supplement: S4 Data — Full AlphaFold2/ColabFold outputs. (GZ) [file pcbi.1010787.s011.tar.gz › KEY84097_1_509/msa_coverage.filtered.png]

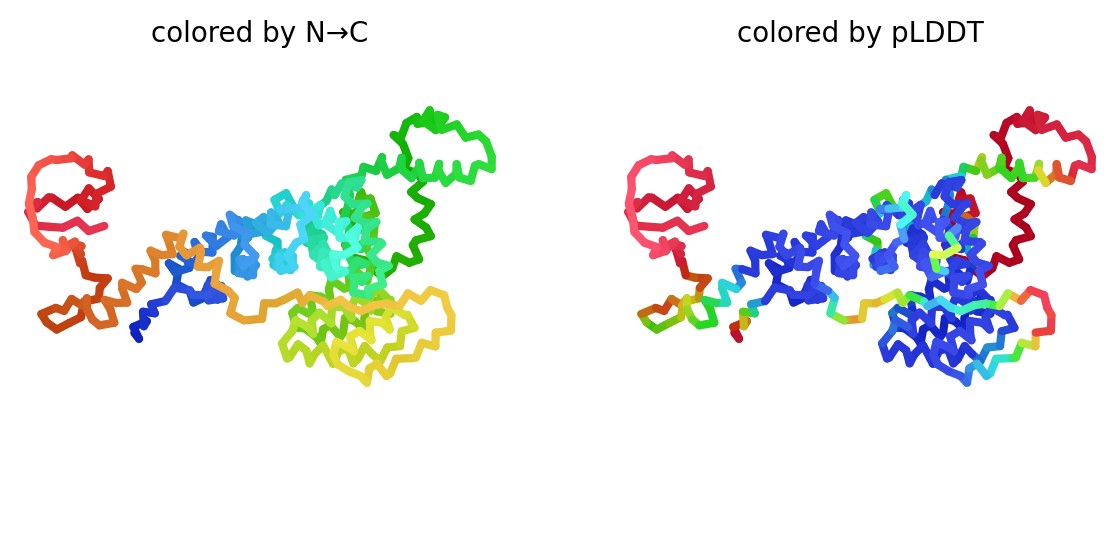

Supplement: S4 Data — Full AlphaFold2/ColabFold outputs. (GZ) [file pcbi.1010787.s011.tar.gz › KEY84097_1_509/rank_4_model_1_ptm_seed_0.png]

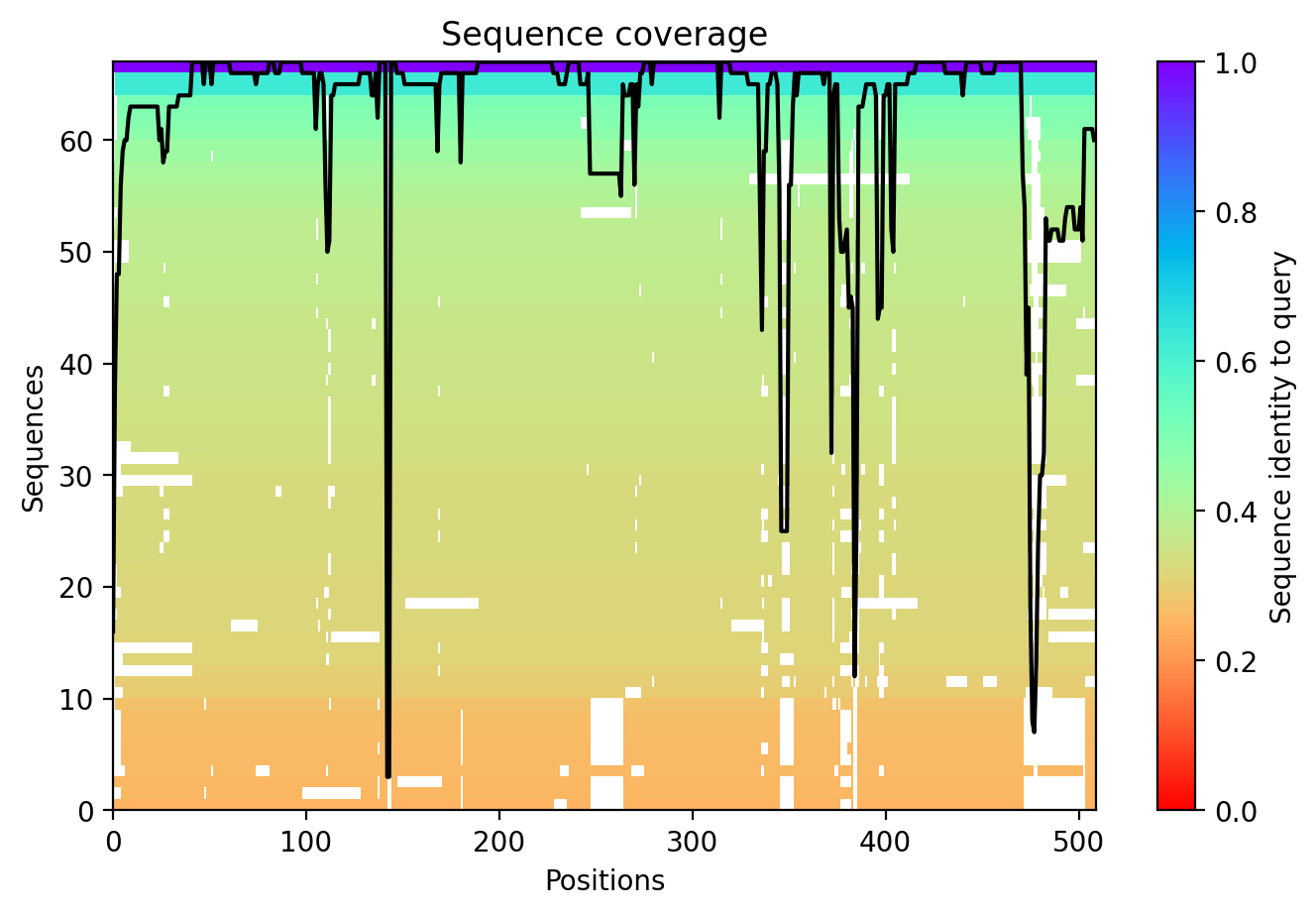

Supplement: S4 Data — Full AlphaFold2/ColabFold outputs. (GZ) [file pcbi.1010787.s011.tar.gz › KEY84097_1_509/msa_coverage.png]

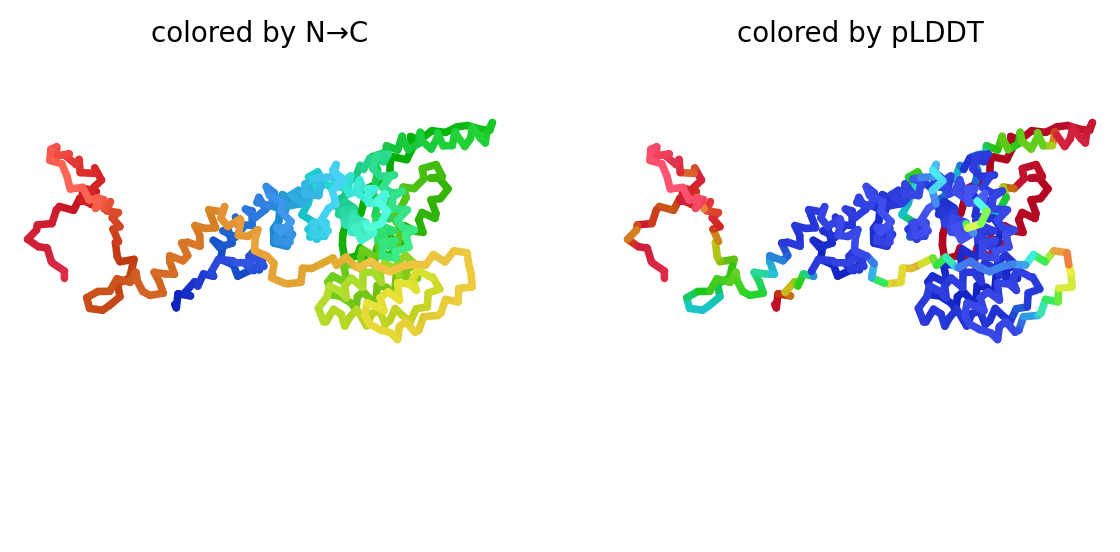

Supplement: S4 Data — Full AlphaFold2/ColabFold outputs. (GZ) [file pcbi.1010787.s011.tar.gz › KEY84097_1_509/rank_1_model_3_ptm_seed_0.png]

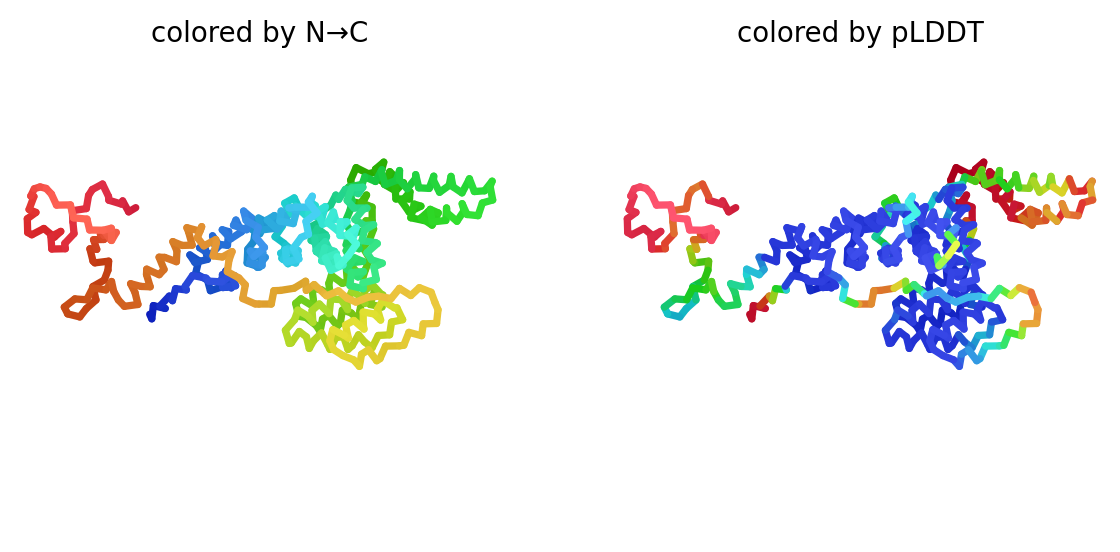

Supplement: S4 Data — Full AlphaFold2/ColabFold outputs. (GZ) [file pcbi.1010787.s011.tar.gz › KEY84097_1_509/rank_2_model_4_ptm_seed_0.png]

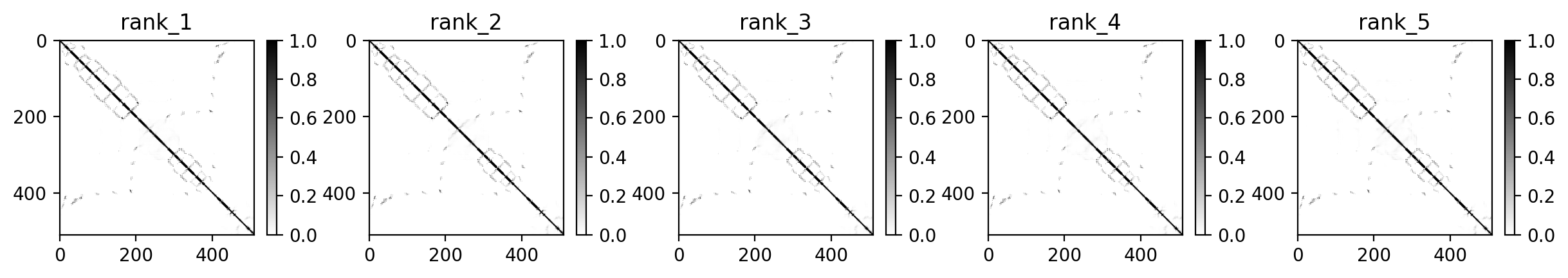

Supplement: S4 Data — Full AlphaFold2/ColabFold outputs. (GZ) [file pcbi.1010787.s011.tar.gz › KEY84097_1_509/predicted_contacts.png]

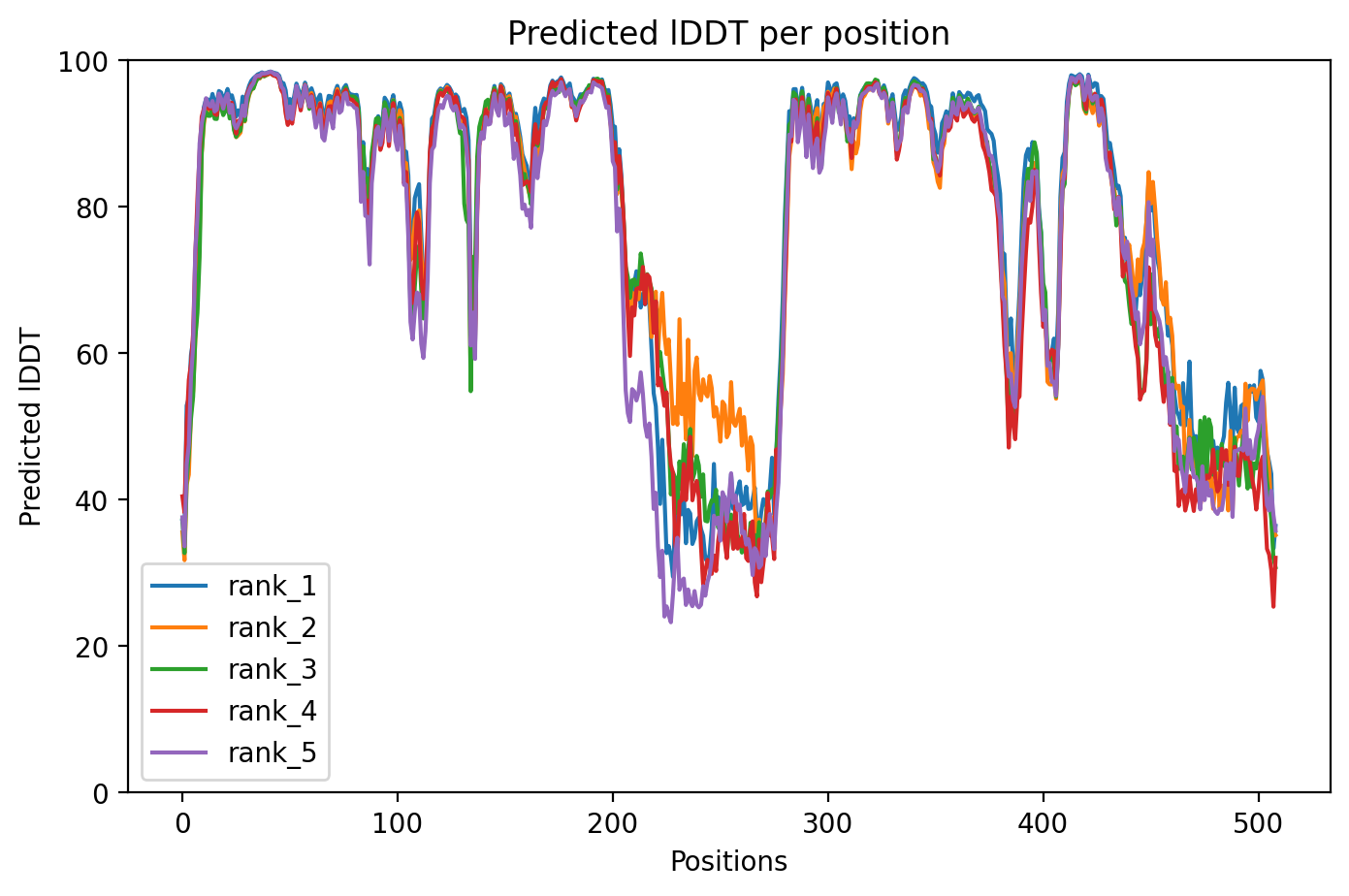

Supplement: S4 Data — Full AlphaFold2/ColabFold outputs. (GZ) [file pcbi.1010787.s011.tar.gz › KEY84097_1_509/predicted_LDDT.png]

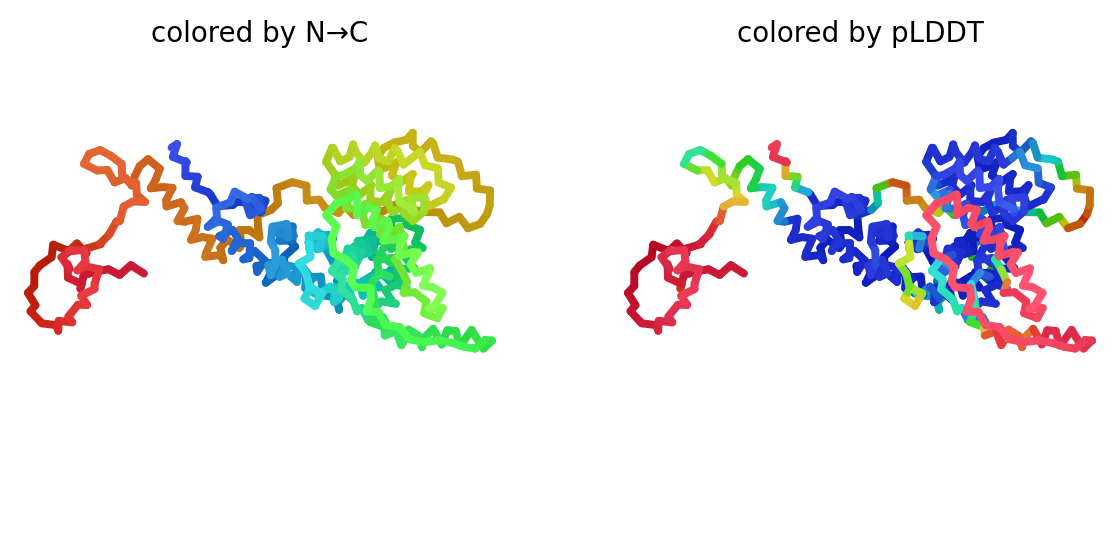

Supplement: S4 Data — Full AlphaFold2/ColabFold outputs. (GZ) [file pcbi.1010787.s011.tar.gz › KEY84097_1_509/rank_5_model_5_ptm_seed_0.png]

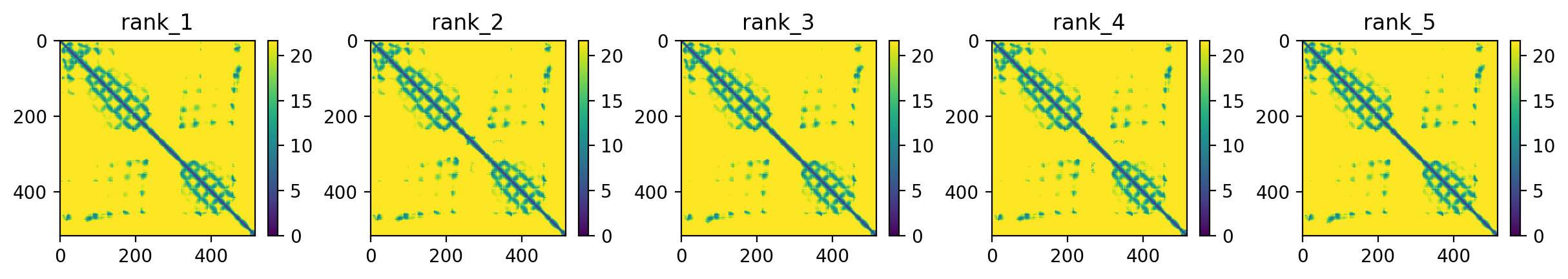

Supplement: S4 Data — Full AlphaFold2/ColabFold outputs. (GZ) [file pcbi.1010787.s011.tar.gz › KFH66451_1_515/predicted_distogram.png]

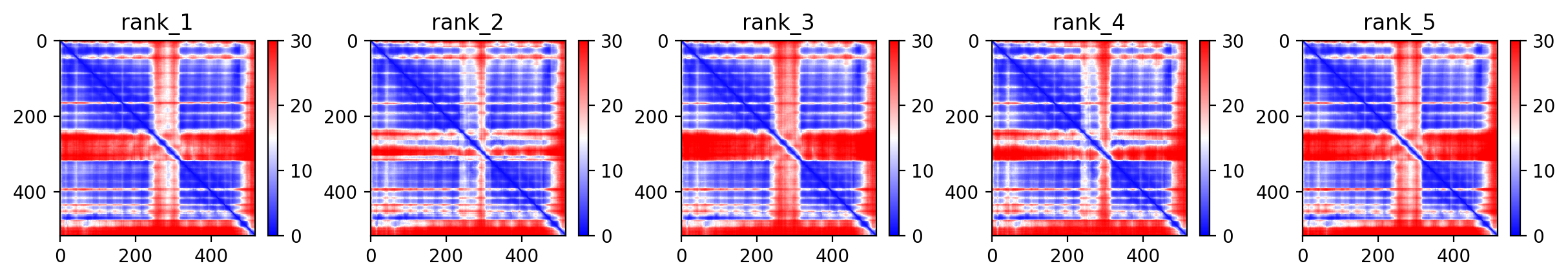

Supplement: S4 Data — Full AlphaFold2/ColabFold outputs. (GZ) [file pcbi.1010787.s011.tar.gz › KFH66451_1_515/predicted_alignment_error.png]

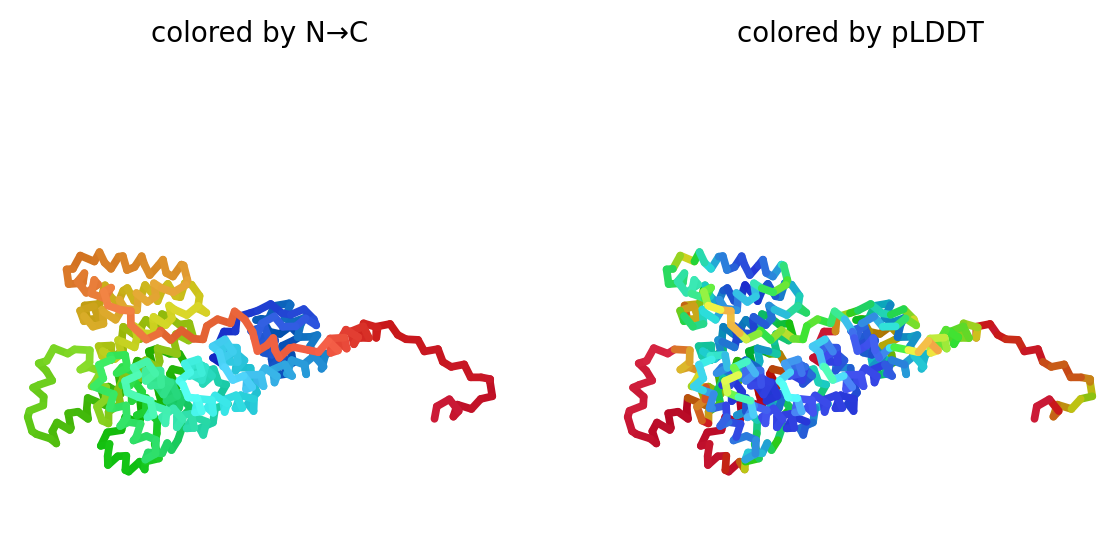

Supplement: S4 Data — Full AlphaFold2/ColabFold outputs. (GZ) [file pcbi.1010787.s011.tar.gz › KFH66451_1_515/rank_2_model_2_ptm_seed_0.png]

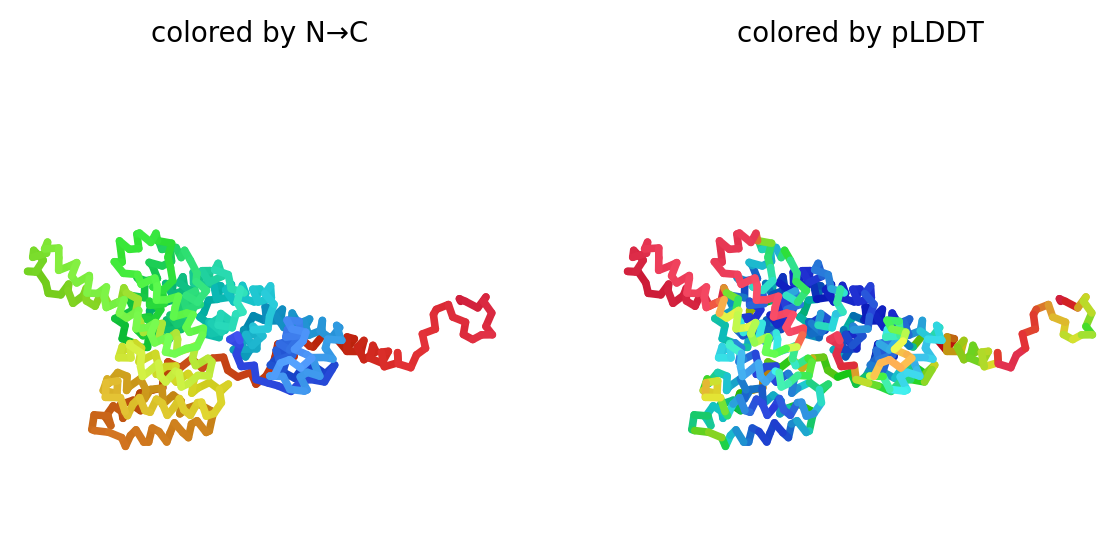

Supplement: S4 Data — Full AlphaFold2/ColabFold outputs. (GZ) [file pcbi.1010787.s011.tar.gz › KFH66451_1_515/rank_4_model_1_ptm_seed_0.png]

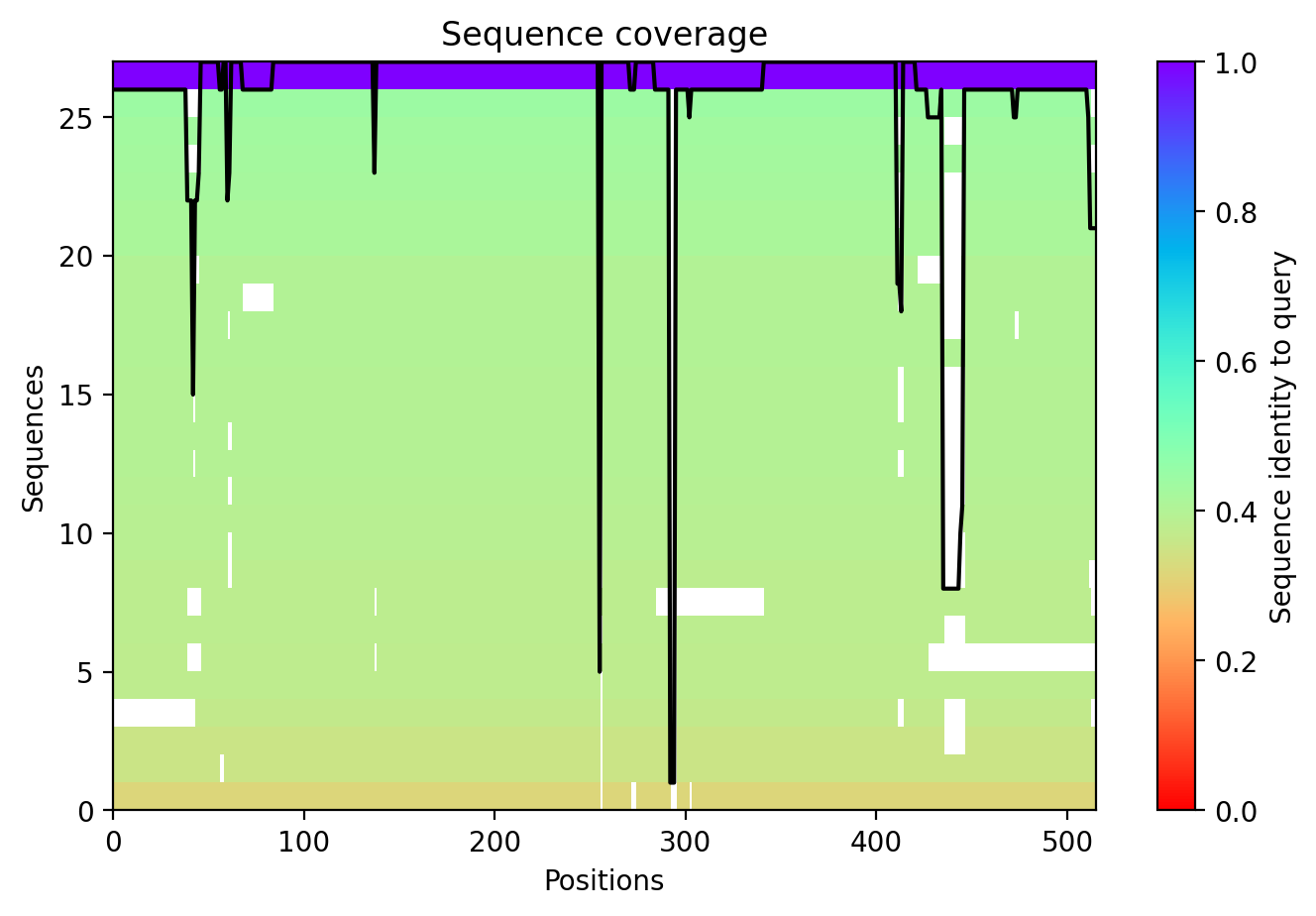

Supplement: S4 Data — Full AlphaFold2/ColabFold outputs. (GZ) [file pcbi.1010787.s011.tar.gz › KFH66451_1_515/msa_coverage.png]

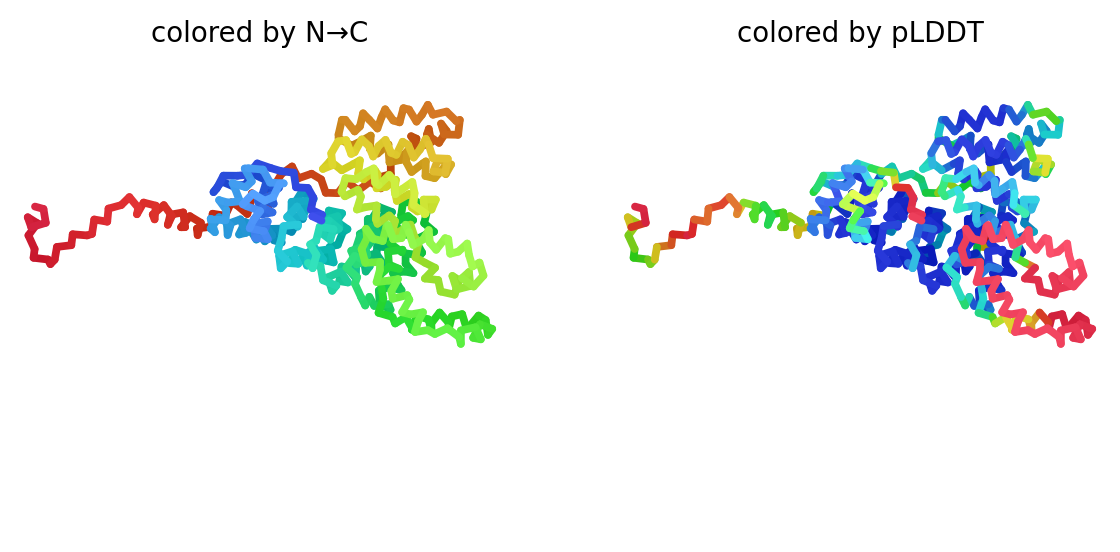

Supplement: S4 Data — Full AlphaFold2/ColabFold outputs. (GZ) [file pcbi.1010787.s011.tar.gz › KFH66451_1_515/rank_1_model_3_ptm_seed_0.png]

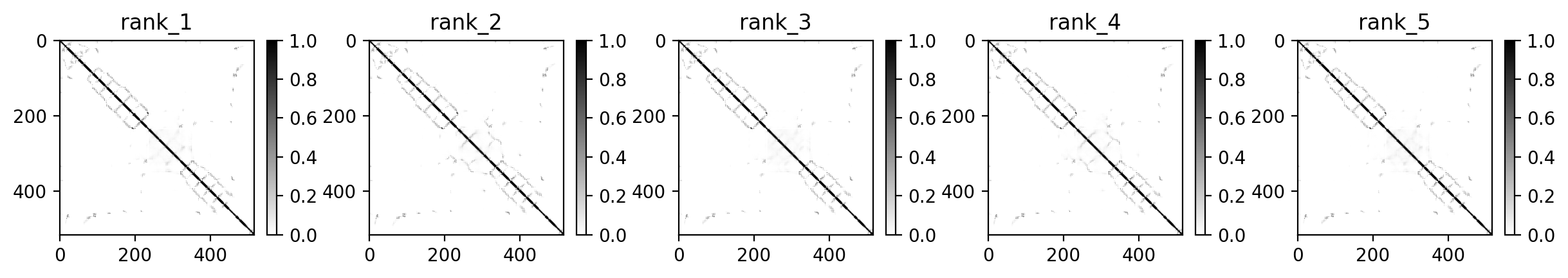

Supplement: S4 Data — Full AlphaFold2/ColabFold outputs. (GZ) [file pcbi.1010787.s011.tar.gz › KFH66451_1_515/predicted_contacts.png]

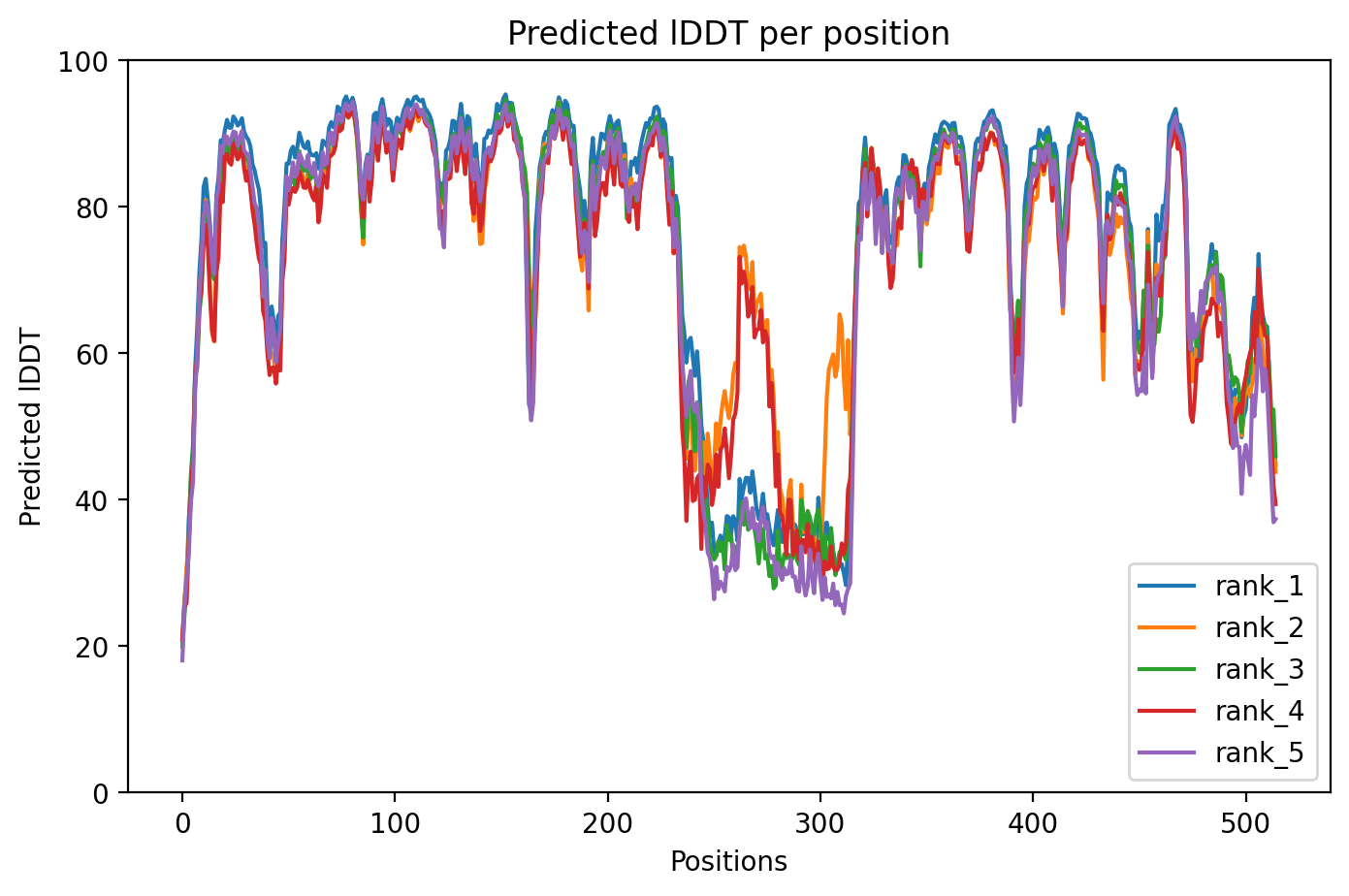

Supplement: S4 Data — Full AlphaFold2/ColabFold outputs. (GZ) [file pcbi.1010787.s011.tar.gz › KFH66451_1_515/predicted_LDDT.png]

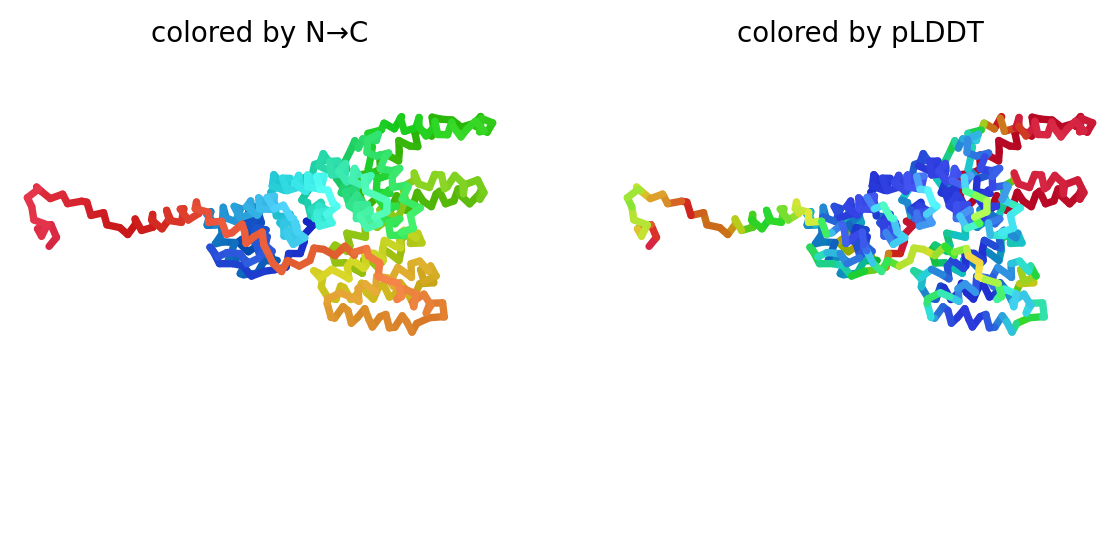

Supplement: S4 Data — Full AlphaFold2/ColabFold outputs. (GZ) [file pcbi.1010787.s011.tar.gz › KFH66451_1_515/rank_3_model_4_ptm_seed_0.png]

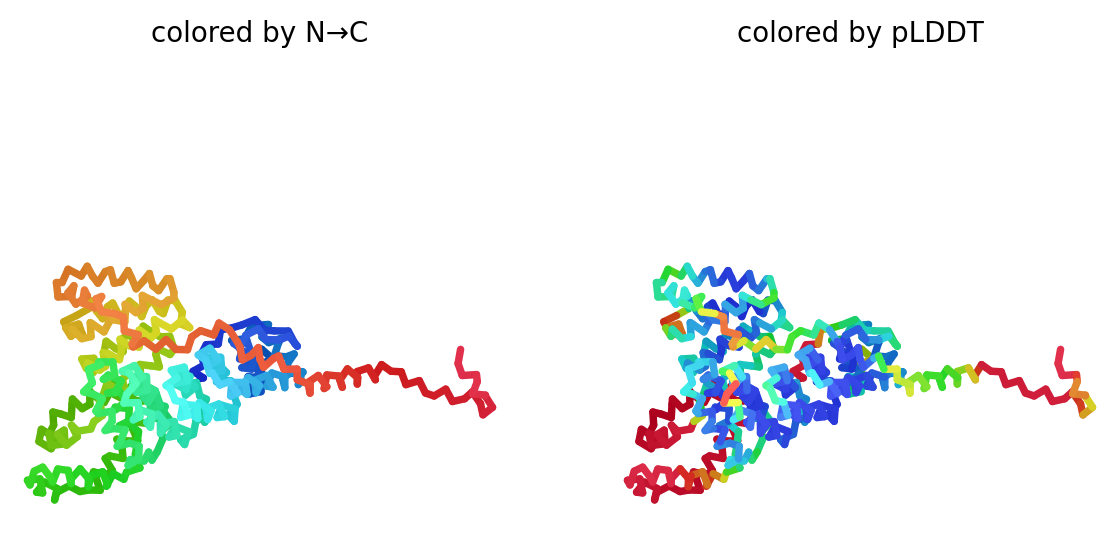

Supplement: S4 Data — Full AlphaFold2/ColabFold outputs. (GZ) [file pcbi.1010787.s011.tar.gz › KFH66451_1_515/rank_5_model_5_ptm_seed_0.png]

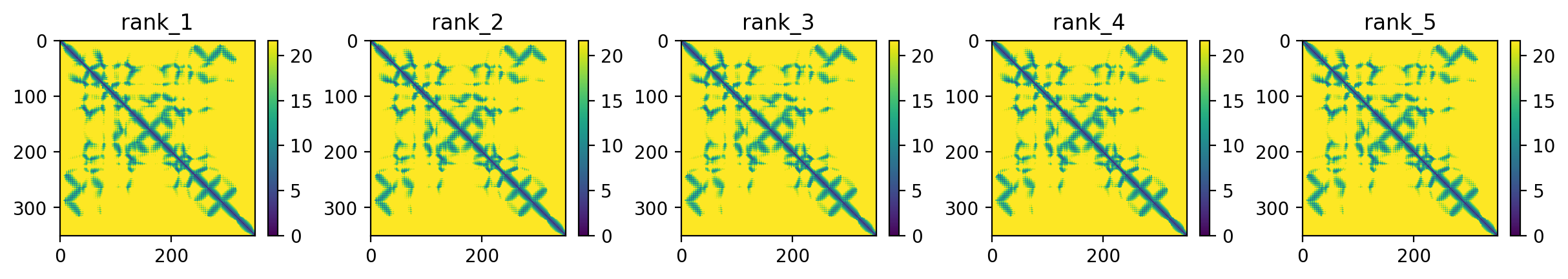

Supplement: S4 Data — Full AlphaFold2/ColabFold outputs. (GZ) [file pcbi.1010787.s011.tar.gz › PQE30996_1_350/predicted_distogram.png]

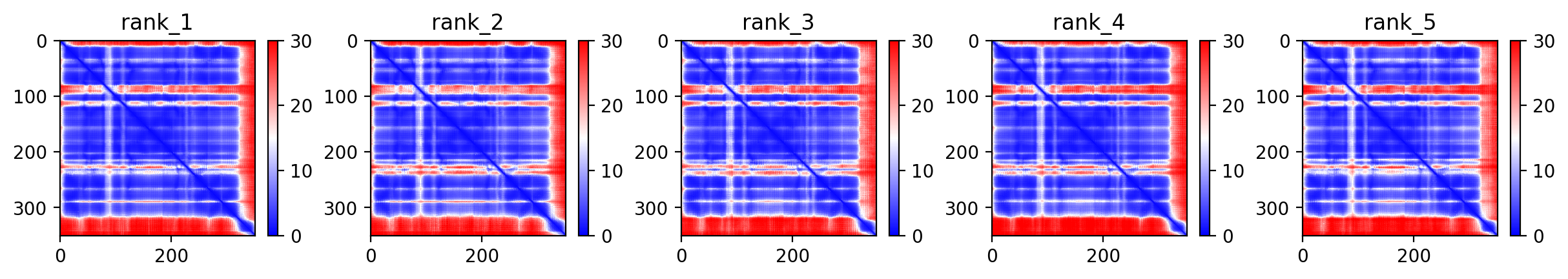

Supplement: S4 Data — Full AlphaFold2/ColabFold outputs. (GZ) [file pcbi.1010787.s011.tar.gz › PQE30996_1_350/predicted_alignment_error.png]

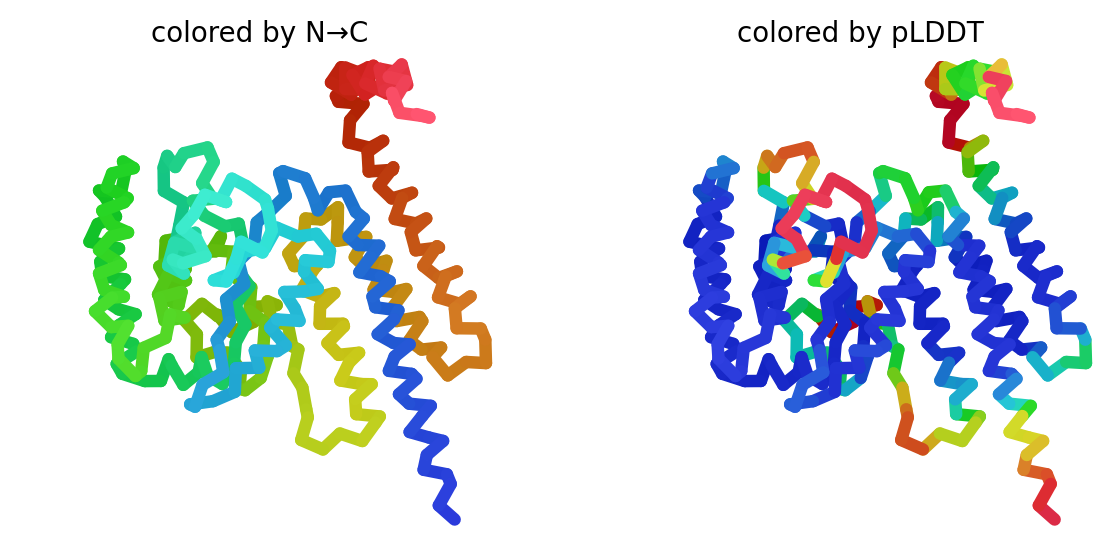

Supplement: S4 Data — Full AlphaFold2/ColabFold outputs. (GZ) [file pcbi.1010787.s011.tar.gz › PQE30996_1_350/rank_3_model_1_ptm_seed_0.png]

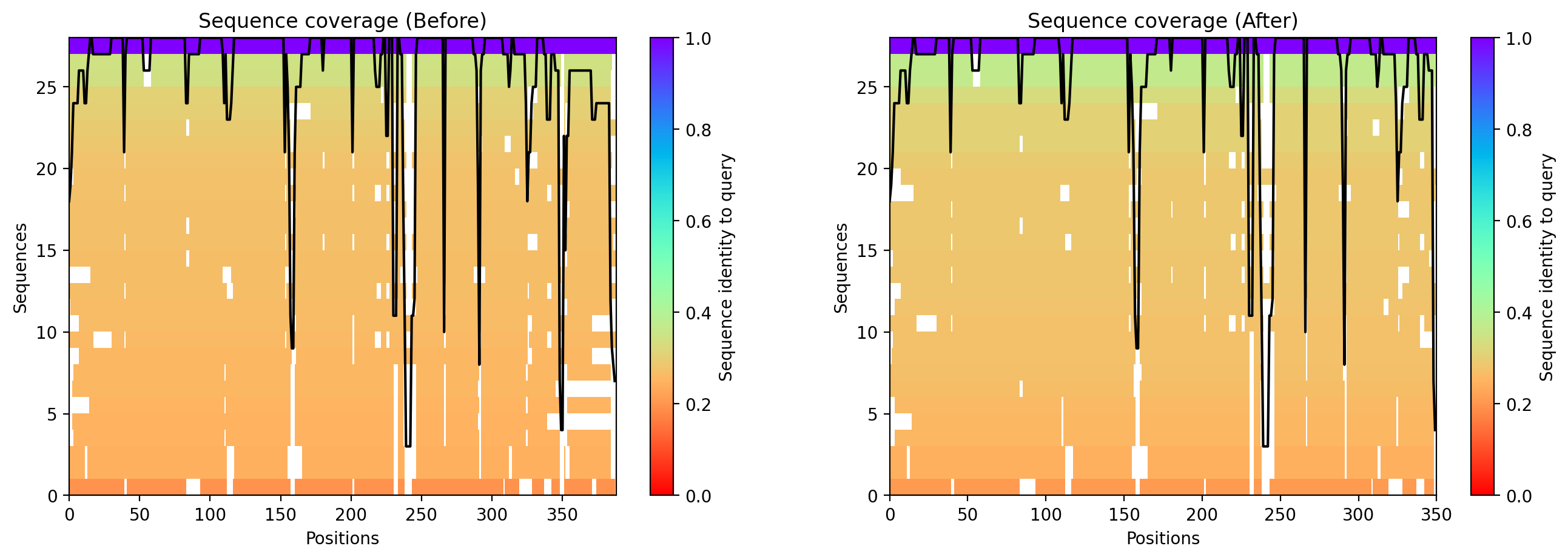

Supplement: S4 Data — Full AlphaFold2/ColabFold outputs. (GZ) [file pcbi.1010787.s011.tar.gz › PQE30996_1_350/msa_coverage.filtered.png]

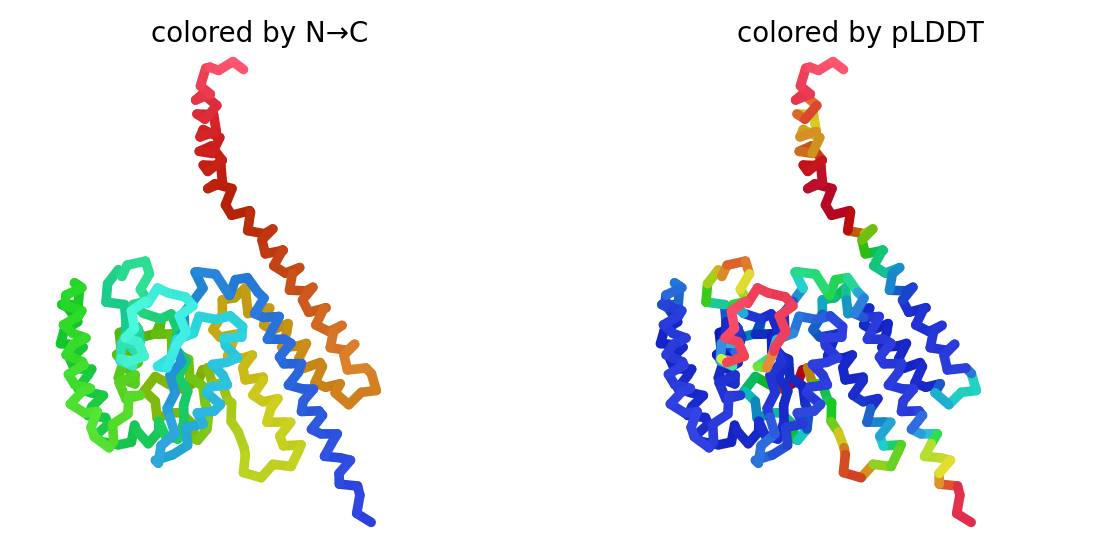

Supplement: S4 Data — Full AlphaFold2/ColabFold outputs. (GZ) [file pcbi.1010787.s011.tar.gz › PQE30996_1_350/rank_4_model_2_ptm_seed_0.png]

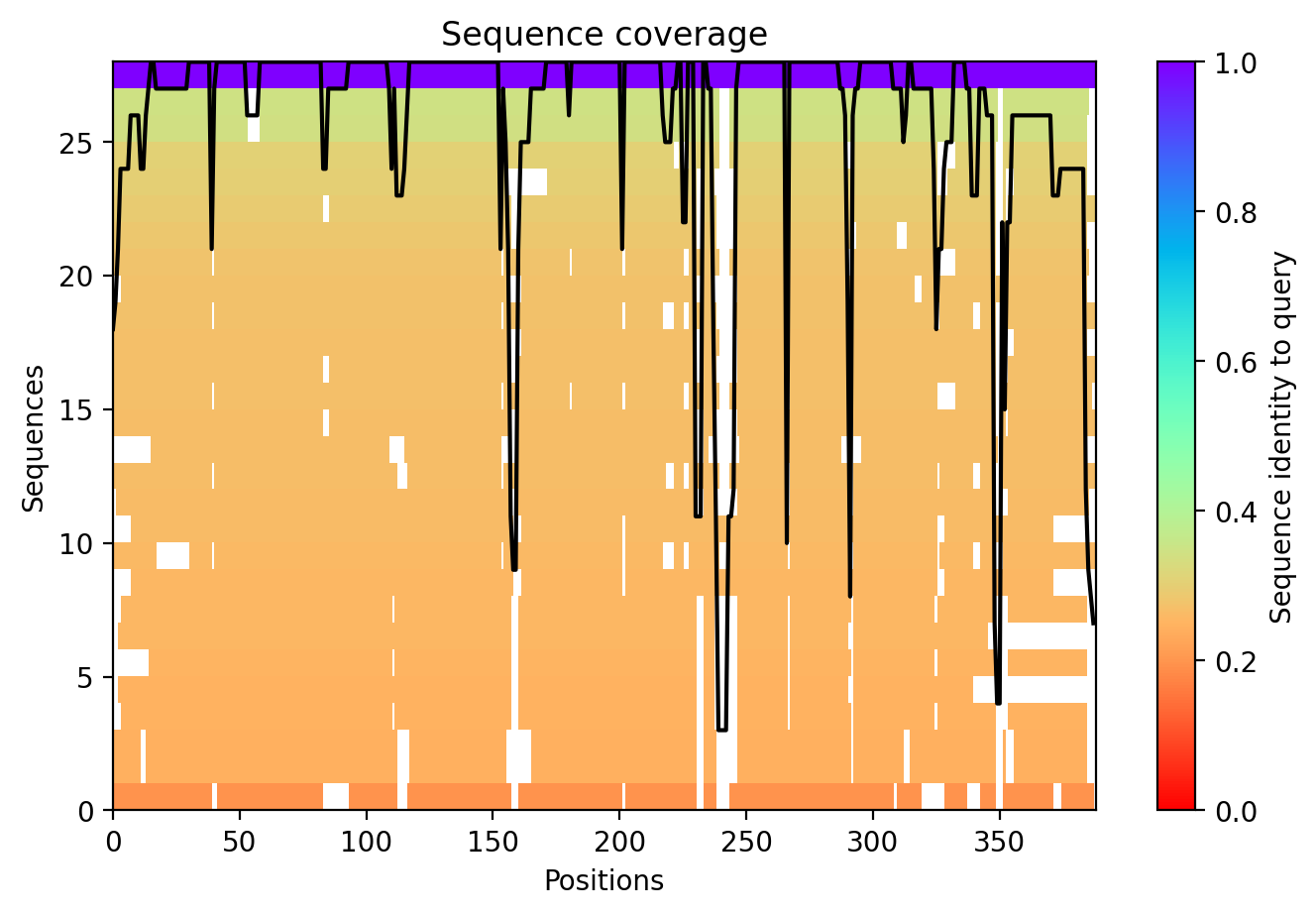

Supplement: S4 Data — Full AlphaFold2/ColabFold outputs. (GZ) [file pcbi.1010787.s011.tar.gz › PQE30996_1_350/msa_coverage.png]

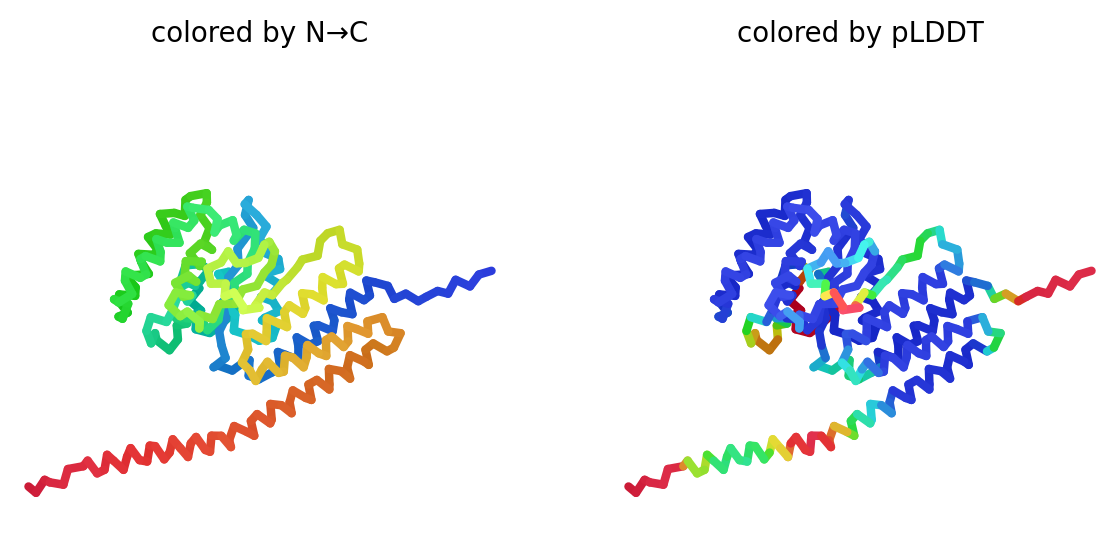

Supplement: S4 Data — Full AlphaFold2/ColabFold outputs. (GZ) [file pcbi.1010787.s011.tar.gz › PQE30996_1_350/rank_1_model_3_ptm_seed_0.png]

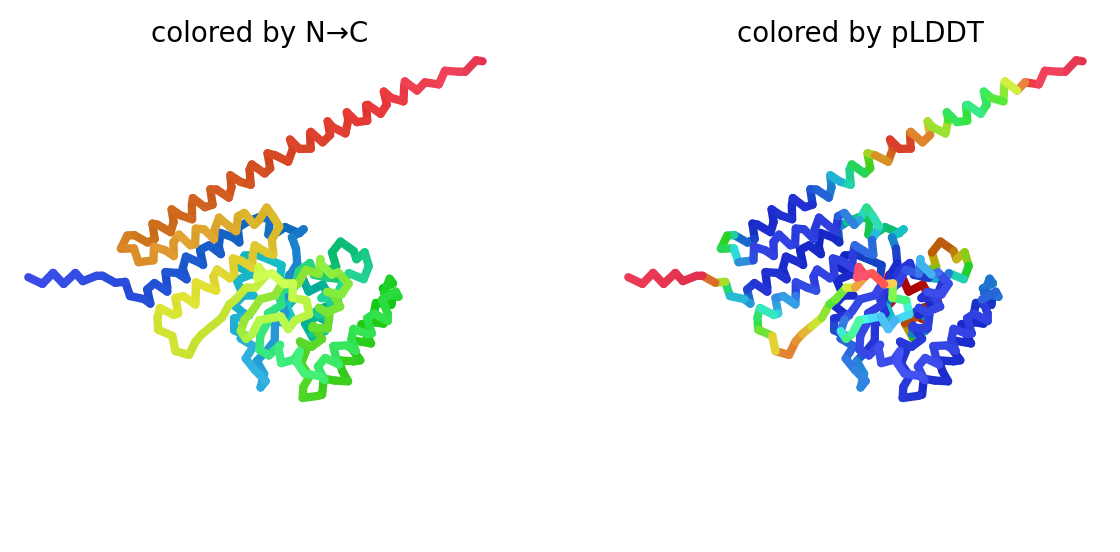

Supplement: S4 Data — Full AlphaFold2/ColabFold outputs. (GZ) [file pcbi.1010787.s011.tar.gz › PQE30996_1_350/rank_2_model_4_ptm_seed_0.png]

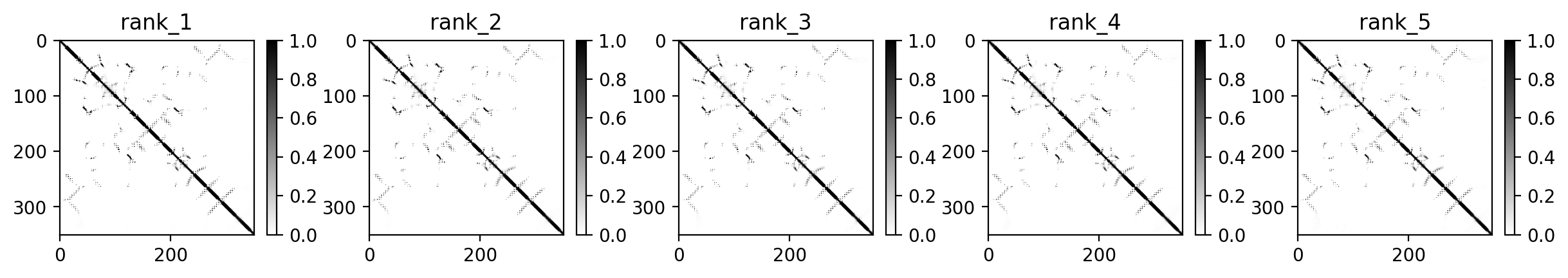

Supplement: S4 Data — Full AlphaFold2/ColabFold outputs. (GZ) [file pcbi.1010787.s011.tar.gz › PQE30996_1_350/predicted_contacts.png]

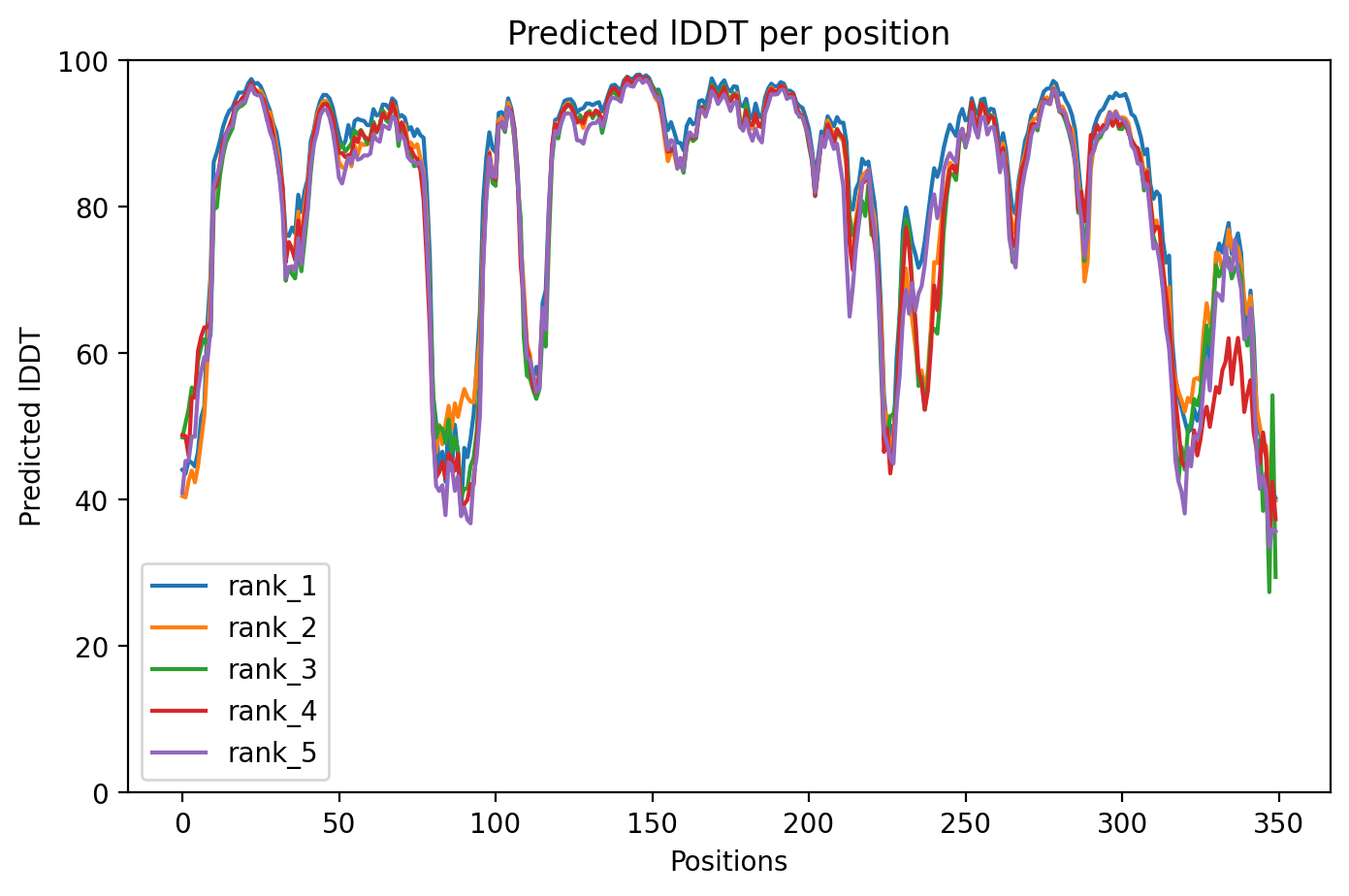

Supplement: S4 Data — Full AlphaFold2/ColabFold outputs. (GZ) [file pcbi.1010787.s011.tar.gz › PQE30996_1_350/predicted_LDDT.png]

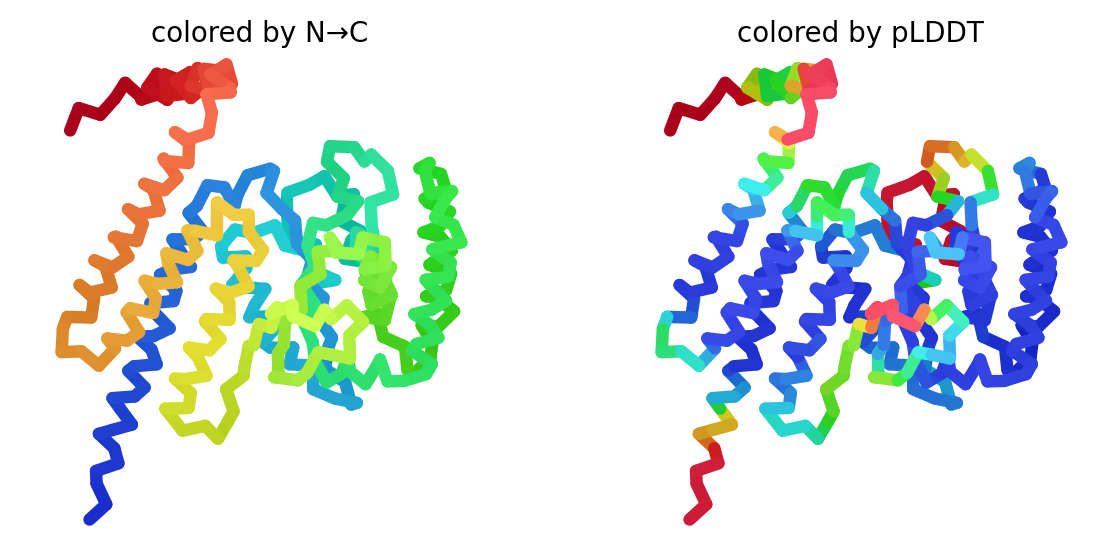

Supplement: S4 Data — Full AlphaFold2/ColabFold outputs. (GZ) [file pcbi.1010787.s011.tar.gz › PQE30996_1_350/rank_5_model_5_ptm_seed_0.png]
